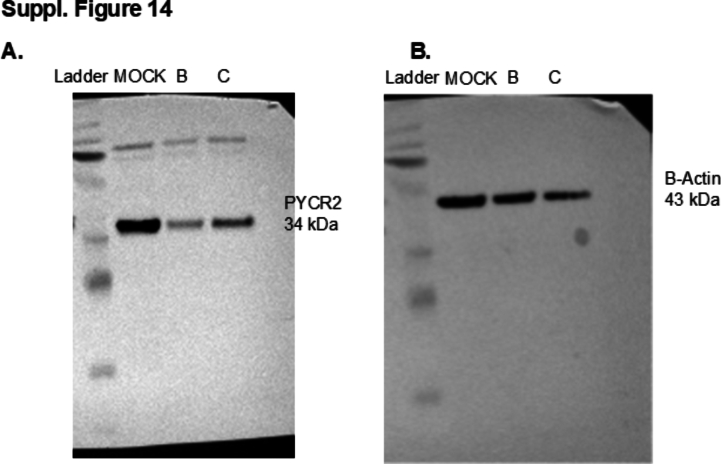# Metabolomic liquid biopsy dynamics predict early-stage HCC and actionable candidates of human hepatocarcinogenesis

**DOI:** 10.1016/j.jhepr.2025.101340

**Published:** 2025-01-30

**Authors:** Kornelius Schulze, Tim Daniel Rose, Lorenz Adlung, Manuela Peschka, Francesca Pagani, Joao Gorgulho, Thorben W. Fründt, Ismail Labgaa, Philipp K. Haber, Carolin Zimpel, Darko Castven, Arndt Weinmann, Teresa Garzia-Lezana, Moritz Waldmann, Thomas Renné, Hannah Voß, Manuela Moritz, Dorian Orlikowski, Hartmut Schlüter, Jan Baumbach, Myron Schwartz, Ansgar W. Lohse, Samuel Huber, Bruno Sangro, Rocio I.R. Macias, Laura Izquierdo-Sanchez, Jesus M. Banales, Henning Wege, Jens U. Marquardt, Augusto Villanueva, Josch Konstantin Pauling, Johann von Felden

**Affiliations:** 1 I. Department of Medicine, University Medical Center Hamburg-Eppendorf, Hamburg, Germany; 2 ERN-RARE-LIVER, Hamburg, Germany; 3 LipiTUM, Chair of Experimental Bioinformatics, TUM School of Life Sciences, Technical University of Munich, Munich, Germany; 4 Structural and Computational Biology Unit, European Molecular Biology Laboratory, Heidelberg, Germany; 5 Hamburg Center for Translational Immunology (HCTI), and Center for Biomedical AI (bAIome), Hamburg, Germany; 6 Mildred Scheel Cancer Career Center HaTriCS4, University Medical Center Hamburg-Eppendorf, Hamburg, Germany; 7 Institute of Clinical Chemistry and Laboratory Medicine, University Medical Center Hamburg-Eppendorf, Hamburg, Germany; 8 Newborn Screening and Metabolic Laboratory, Department of Pediatrics, University Medical Center Hamburg-Eppendorf, Hamburg, Germany; 9 Department of Oncology, Hematology and Bone Marrow Transplantation with Section of Pneumology, University Medical Centre Hamburg-Eppendorf, Hamburg, Germany; 10 University Cancer Center Hamburg–Hubertus Wald Tumorzentrum, University Medical Centre Hamburg-Eppendorf, Hamburg, Germany; 11 Division of Liver Diseases, Liver Cancer Program, Tisch Cancer Institute, Department of Medicine, Icahn School of Medicine at Mount Sinai, New York, NY, USA; 12 Department of Visceral Surgery, Lausanne University Hospital (CHUV), University of Lausanne (UNIL), Lausanne, Switzerland; 13 Department of Medicine I, University Medical Center Schleswig-Holstein-Campus Lübeck, Germany; 14 I. Department of Medicine, University Medical Center Mainz, Germany; 15 Irish Centre for Vascular Biology, School of Pharmacy and Biomolecular Sciences, Royal College of Surgeons in Ireland, Dublin, Ireland; 16 Center for Thrombosis and Hemostasis (CTH), Johannes Gutenberg University Medical Center, Mainz, Germany; 17 Chair of Computational Systems Biology, University of Hamburg, 22607 Hamburg, Germany; 18 Recanati Miller Transplant Institute, The Icahn School of Medicine at Mount Sinai Hospital, New York, NY, USA; 19 Liver Unit, Clinica Universidad de Navarra-IDISNA and CIBEREHD, Pamplona, Spain; 20 Center for the Study of Liver and Gastrointestinal Diseases (CIBEREHD), Carlos III National Institute of Health, Madrid, Spain; 21 Experimental Hepatology and Drug Targeting (HEVEPHARM), University of Salamanca, IBSAL, Salamanca, Spain; 22 Department of Liver and Gastrointestinal Diseases, Biogipuzkoa Health Research Institute–Donostia University Hospital, University of the Basque Country (UPV/EHU), CIBEREHD, Donostia-San Sebastian, Spain; 23 IKERBASQUE, Basque Foundation for Science, Bilbao, Spain; 24 Department of Biochemistry and Genetics, School of Sciences, University of Navarra, Pamplona, Spain; 25 Division of Hematology and Medical Oncology, Department of Medicine, Icahn School of Medicine at Mount Sinai, New York, NY, USA; 26 Institute for Clinical Chemistry and Laboratory Medicine, University Hospital and Faculty of Medicine Carl Gustav Carus, Dresden University of Technology, Dresden, Germany

**Keywords:** Liver cancer, Tumorigenesis, Prevention, Early detection, Surveillance, Metabolism

## Abstract

**Background & Aims:**

Actionable candidates of hepatocarcinogenesis remain elusive, and tools for early detection are suboptimal. Our aim was to demonstrate that serum metabolome profiles reflect the initiation of hepatocellular carcinoma (HCC) and enable the identification of biomarkers for early HCC detection and actionable candidates for chemoprevention.

**Methods:**

This global cohort study included 654 patients and 801 biospecimens. Following serum metabolome profiling across the spectrum of hepatocarcinogenesis, we conducted a phase II biomarker case–control study for early HCC detection. Findings were independently validated through *in silico* analysis, mRNA sequencing, and proteome profiling of primary HCC and non-tumoral tissue, and *in vitro* experiments.

**Results:**

Aspartic acid, glutamic acid, taurine, and hypoxanthine were differentially abundant in the serum across chronic liver disease, cirrhosis, initial HCC, and progressed HCC, independent of sex, age, and etiology. In a phase II biomarker case–control study, a blood-based metabolite signature yielded an AUC of 94% to discriminate between patients with early-stage HCC and controls with cirrhosis, including independent validation. Unsupervised biclustering (MoSBi), lipid network analysis (LINEX^2^), and pathway enrichment analysis confirmed alterations in amino acid-, lipid-, and nucleotide-related pathways. In tumor tissue, these pathways were significantly deregulated regarding gene and protein expression in two independent datasets, including actionable targets RRM2, GMPS, BCAT1, PYCR2, and NEU1. *In vitro* knockdown confirmed a functional role in proliferation and migration, as exemplified for PYCR2.

**Conclusions:**

These findings demonstrate that serum metabolome profiling indicates deregulated metabolites and pathways during hepatocarcinogenesis. Our liquid biopsy approach accurately detects early-stage HCC outperforming currently recommended surveillance tools and facilitates identification of actionable candidates for chemoprevention.

**Impact and implications:**

Deregulated cellular metabolism is a hallmark of cancer. In smaller studies, circulating metabolite profiles have been associated with HCC, although mainly in the context of fatty liver disease. Translation strategies for primary prevention or early detection are lacking. In this global study, we present an unsupervised landscape of the altered serum metabolome profile during hepatocarcinogenesis, independent of age, sex, and etiology. We provide a blood-based metabolite signature that accurately identifies early-stage HCC in a phase II biomarker study including independent validation. Further RRM2, GMPS, BCAT1, PYCR2, and NEU1 are identified in tumor tissue as actionable candidates for prevention. Our data provide the rationale for clinical trials testing liquid biopsy metabolome-based signatures for early HCC detection and the development of chemoprevention strategies.

## Highlights

• The serum metabolome indicates significant alterations during hepatocarcinogenesis.
• Amino acid-, lipid-, and nucleotide-related pathways are significantly deregulated.
• A blood-based metabolite signature from serum accurately identifies early-stage HCC.
• RRM2, GMPS, BCAT1, PYCR2, and NEU1 represent actionable candidates for prevention.

## Graphical abstract

**Figure:**
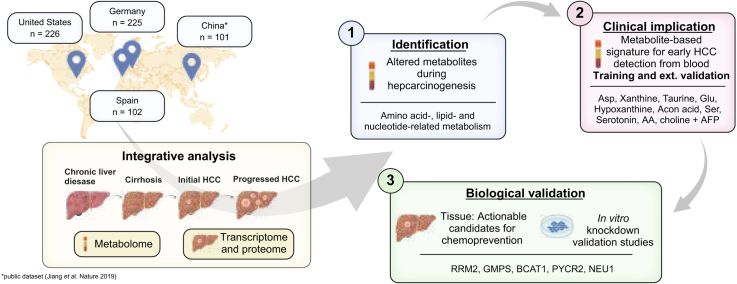

## Introduction

Liver cancer mortality and incidence are steadily increasing.^1^ Its most frequent form is hepatocellular carcinoma (HCC) at approximately 90%,^2^ which typically arises in patients with chronic liver disease (CLD), particularly in the context of cirrhosis.^2^^,^^3^ This stepwise process is mainly induced by chronic inflammation.^4^ Despite this well-defined population at risk, recommended tools for early detection are suboptimal,^5^^,^^6^ and mechanisms of hepatocarcinogenesis remain poorly understood.^4^ Apart from TERT promoter mutations^4^ and DNA methylation changes,^7^ there is limited knowledge regarding early events and mechanisms that drive hepatocarcinogenesis.

Deregulated cellular metabolism is a hallmark of cancer,^8^ and the liver functions as the major metabolic organ in humans. Therefore, aberrations of metabolic pathways in CLD and during HCC evolution are obvious. In fact, multiple alterations in liver cancer metabolism have been reported, and their clinical relevance has been emphasized.^9^ Circulating metabolites have previously been identified to discriminate between HCC and controls; however, cohorts were small and/or limited to the context of fatty liver disease.[10], [11], [12]

This study addresses the two aforementioned clinical needs to develop more accurate tools for early HCC detection and to identify actionable targets during hepatocarcinogenesis. We hypothesize that serum metabolome profiles directly reflect the initiation of HCC and thus enable the identification of biomarkers for early HCC detection and actionable candidates for chemoprevention.

To test our hypothesis, we mapped alterations in serum metabolome profiles across the progressing stages of human hepatocarcinogenesis, including patients with CLD with and without cirrhosis, initial HCC, and progressed HCC. We identified significantly altered metabolites and associated pathways (*i.e.* amino acid-, lipid-, and nucleotide-related pathways) linked to stepwise transformation towards HCC. We developed a metabolite-based signature from blood, which accurately identified early-stage HCC. Finally, gene expression and protein abundance of key metabolic enzymes in primary HCC identified actionable candidates during cancer initiation, including functional knockdown experiments.

Altogether, we provide novel insights into metabolic deregulation of HCC initiation and introduce its clinical implications, such as early detection and chemoprevention.

## Patients and methods

### Patient enrollment and specimen collection

**Fig. 1:**
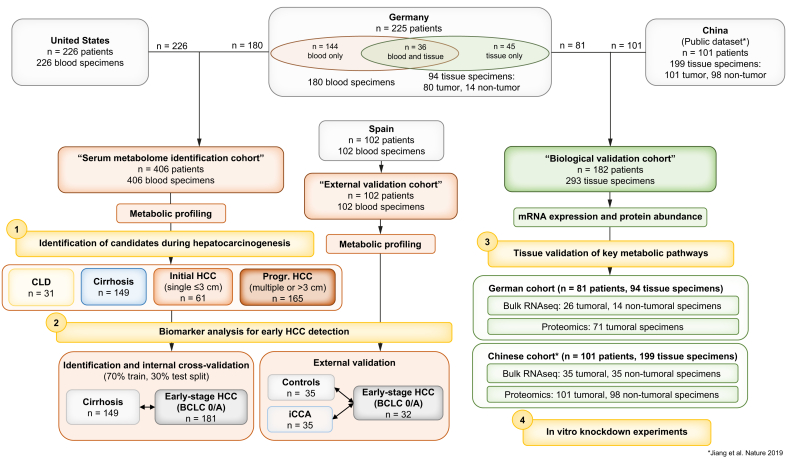
Study overview. Outline of cohort distribution and experimental workflow. A total of 406 blood specimens from the USA and Germany were combined into the “serum metabolome identification cohort” for the identification of candidates during hepatocarcinogenesis (1) and a subset used for the biomarker analysis for early HCC detection (2). In addition, 102 blood specimens from Spain were used as an “external validation cohort” for the biomarker analysis (2). Biological validation of key metabolic pathways was conducted in 182 patients with 293 available tissue specimens (not all patients had paired tumor and non-tumoral tissue available) from the German (internal cohort) and Chinese cohorts by bulk mRNA-sequencing and proteomics analysis (3), alongside with *in vitro* studies (4). Some patients from the German cohort were included in both the “serum metabolome identification cohort” and the “biological validation cohort,” according to availability of specimens. BCLC, Barcelona Clinic for Liver Cancer; CLD, chronic liver disease; HCC, hepatocellular carcinoma; iCCA, intrahepatic cholangiocarcinoma; RNAseq, RNA sequencing.

In this multicenter, global study, 654 patients and 801 biospecimens were analyzed (Fig. 1). A total of 553 patients were actively enrolled from six different centers across three countries: USA: Mount Sinai Hospital in New York City, NY, n = 226 (serum only); Germany: University Medical Center Hamburg-Eppendorf n = 187 (n = 106 serum only, n = 36 paired serum and tissue, and n = 45 tissue only) and University Medical Center Mainz n = 38 (serum only); and Spain (all serum only): University Medical Center San Sebastian n = 52, University Medical Center Salamanca (National DNA Bank-Carlos III) n = 28, and University Medical Center Pamplona n = 23. In addition, 101 patients were accessed from an online dataset from China (tissue data only).^13^ The study was conducted in accordance with both the Declarations of Helsinki and Istanbul. All research was approved by the local ethics committee (New York: HS-15-00540, Hamburg: PV-3578, Mainz: 837.199.10, San Sebastian: PI2019116, Salamanca: 21102016, and Pamplona: 2017.012), and written consent was given in writing by all participants.

Diagnosis of cirrhosis and/or HCC was made according to clinical guidelines.[14], [15], [16] Patients with concurrent malignancies were excluded.

Serum samples from Germany and the USA were combined into the “serum metabolome identification cohort” (N = 406) and grouped into patients with (i) CLD without cirrhosis (n = 31), (ii) CLD with cirrhosis (n = 149), (iii) initial HCC (*i.e.* single HCC nodule ≤3 cm in diameter, no extrahepatic disease) (n = 61), and (iv) progressed HCC (*i.e.* multiple HCC nodules or single nodule >3 cm in diameter) (n = 165) (for detailed clinical characteristics, see Table 1). The threshold of 3 cm was chosen to balance between biological (risk of occult metastasis^17^^,^^18^) and statistical (to obtain more equal group sizes) concerns. All blood specimens were collected during routine clinical management of patients and stored at -80 °C until further analysis.

**Table 1:** Clinical characteristics of “serum metabolome identification cohort” (N = 406).

Characteristic	Overall (N = 406)∗	CLD (n = 31)∗	Cirrhosis (n = 149)∗	Initial HCC (n= 61)∗	Progressed HCC (n = 165)∗	*p* value†
Sex						<0.001
Female	108 (30)	11 (35)	61 (41)	10 (16)	26 (21)	
Male	257 (70)	20 (65)	88 (59)	51 (84)	98 (79)	
Age (years)						0.056
<60	123 (34)	12 (39)	61 (41)	16 (26)	34 (27)	
≥60	242 (66)	19 (61)	88 (59)	45 (74)	90 (73)	
Diabetes	97 (37)	4 (22)	17 (38)	21 (36)	55 (39)	0.6
BMI (kg/m^2^)	26.4 (23.1–30.9)	NA	26.6 (23.7–30.8)	26.0 (23.3–29.4)	26.4 (23.1–30.9)	>0.9
Etiology						
Alcohol	81 (20)	1 (3.2)	45 (30)	7 (11)	28 (17)	
NASH	52 (13)	0 (0)	17 (11)	13 (21)	22 (13)	
Viral	183 (45)	29 (94)	49 (33)	31 (51)	74 (45)	
Other	90 (22)	1 (3.2)	38 (26)	10 (16)	41 (25)	
Cirrhosis	290 (72)	0 (0)	149 (100)	47 (77)	94 (58)	<0.001
Child–Pugh stage‡						0.3
A	159 (71)	NA	90 (67)	26 (70)	43 (83)	
B	62 (28)	NA	42 (31)	11 (30)	9 (17)	
C	2 (0.9)	NA	2 (1.5)	0 (0)	0 (0)	
BCLC stage						<0.001
0	27 (12)	NA	NA	27 (44)	0 (0)	
A	154 (68)	NA	NA	34 (56)	120 (73)	
B	19 (8.4)	NA	NA	0 (0)	19 (12)	
C	25 (11)	NA	NA	0 (0)	25 (15)	
D	1 (0.4)	NA	NA	0 (0)	1 (0.6)	
AFP (ng/ml)	5 (3–25)	3 (2–4)	4 (2–5)	6 (4–27)	16 (5–304)	<0.001

AFP, alpha fetoprotein; BCLC, Barcelona Clinic for Liver Cancer; CLD, chronic liver disease; HCC, hepatocellular carcinoma; NA, not applicable; NASH, non-alcoholic steatohepatitis.

∗ Statistics are presented as n (%) or median (IQR).

† Statistical tests performed: Chi-square test of independence, Kruskal–Wallis test, and Fisher’s exact test.

‡ Only calculated for patients with cirrhosis.

For RNA and proteome analysis from tissue, 81 patients from the Hamburg cohort with 94 available tissue specimens (80 HCC tumoral tissue and 14 adjacent non-tumoral tissue specimens) were included. Of these patients, 36 were part of the serum metabolomics analysis with paired serum specimens (see below for details on sample collection and processing).

In addition, a publicly available RNA sequencing (RNAseq) and proteome dataset,^13^ including 101 patients with 101 HCC tissue specimens and 98 paired adjacent non-tumoral tissue specimens, was used for external validation of our findings. Clinical data for the overall population of these patients were retrieved from the respective publication: 85% were male patients, predominantly with hepatitis B infection (98%). Furthermore, 82% had cirrhosis, and tumors were all stage 0 or A according to the Barcelona Clinic for Liver Cancer (BCLC) staging.^13^

### Metabolomic profiling from serum specimens and pathway analysis

US and German serum samples were processed using the MxP® Quant 500 Kit (BIOCRATES Life Sciences AG, Innsbruck, Austria) according to the manufacturer’s instructions. Spanish samples (external validation cohort) were analyzed in two ultra-high performance liquid chromatography (UHPLC)–time of flight–MS-based platforms, as previously described.^11^

### Bulk mRNA sequencing from tissue specimen

For the Hamburg cohort, fresh tumoral and adjacent non-tumoral tissue specimens were collected during liver resection surgery for HCC. Total RNA was extracted from tissue using the Rneasy® Plus Mini Kit (Qiagen, Hilden, Germany) according to the manufacturer’s instructions. After library construction, sequencing was performed using Illumina Novaseq 6000 (Illumina, Inc., San Diego, CA, USA).

### Proteome profiling from tissue specimen

For the Hamburg cohort, protein was extracted from formalin-fixed paraffin-embedded (FFPE) tissue specimens with tryptic digestion as previously described.^19^ Subsequently, 1 μg of peptides was subjected to liquid chromatography–tandem mass spectrometry (LC-MS/MS) measurements. Raw LC-MS/MS spectra were searched using the Sequest algorithm integrated into the Proteome Discoverer software (version 2.41.15, Thermo Fisher Scientific) against a reviewed human Swissprot database. Protein quantification was carried out using the Minora algorithm, implemented in Proteome Discoverer.^20^

### Data analysis

Metabolomics data was log_2_ transformed, and the limma R package^21^ was used to remove cohort batch effects while preserving patient groups (Fig. S1). Biclustering analysis was performed using the Molecular Signature identification using Biclustering (MoSBi) ensemble approach.^22^

For the phase II biomarker analysis, classifications were performed using random forest models. The data were randomly separated into training (70%) and test data (30%). This was repeated 1,000 times for each scenario. Average performance metrics (sensitivity, specificity, and AUC for receiver operating characteristic [ROC], and precision–recall [PR]) are reported for the test data. Single candidate analysis was conducted using R package cutpointr.

The LipidNetworkExplorer LINEX^2^ software^23^ was used for the network analysis of lipids from the metabolome profiling. For this analysis, all lipids that were part of the metabolomics panel were included (Table S1).

Differentially expressed genes from bulk mRNA sequencing data were identified using normalized counts processed with the DESeq2 package. The Wilcoxon rank-sum test with *post hoc* correction for multiple testing was applied, similar to the dataset from Jiang *et al.*^13^ Protein abundance was determined using within-gene and within-sample normalized values obtained by proteomics measurements (see Supplementary material). Comparison on a gene-by-gene level was performed against the dataset from Jiang *et al.*^13^

For descriptive statistics, continuous variables are reported as median and IQRs, and categorical variables are presented as counts and percentages. We used Fisher’s exact test and Student’s *t* test, the Kruskal–Wallis test, or ANOVA to compare differences between categorical and continuous variables. For correlation analysis, Pearson’s correlation coefficient was used for continuous variables. Ordinal logistic regression was performed using the MASS R package. A *p* value less than 0.05 was considered statistically significant, and corrections for multiple comparisons were carried out where needed (false discovery rate [FDR] approach). All statistical and bioinformatic analyses were calculated in R studio (version 4.2).

Detailed information regarding metabolome profiling, mRNA sequencing, proteome profiling, and *in vitro* knockdown experiments is reported in the Supplementary material and Supplementary CTAT table.

## Results

The study design is summarized in Fig. 1. Overall, we analyzed 654 patients and 801 biospecimens across four countries, including an external, publicly available dataset from China.^13^

### Discriminative capacity of serum metabolome profiling during hepatocarcinogenesis

**Fig. 2:**
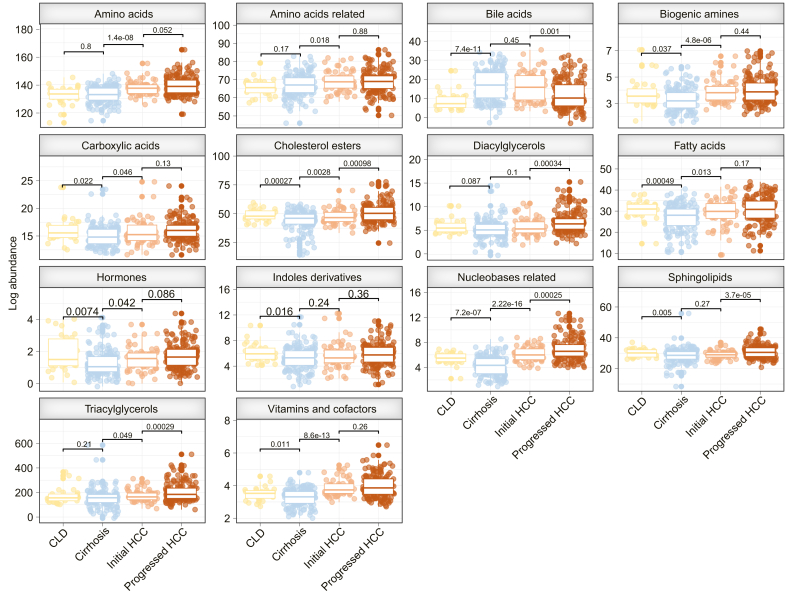
Dynamic changes in serum metabolome. Abundance of significantly altered metabolite classes in sera across patients with CLD, cirrhosis, initial HCC, and progressed HCC. Display limited to metabolite classes with at least one significantly different group comparison for CLD *vs*. cirrhosis, cirrhosis *vs*. initial HCC, and initial HCC *vs*. progressed HCC. Student’s *t* test. CLD, chronic liver disease; HCC, hepatocellular carcinoma.

Serum samples from Germany and USA were combined into the “serum metabolome identification cohort” and grouped into patients with (i) CLD without cirrhosis (n = 31), (ii) cirrhosis (n = 149), (iii) initial HCC (*i.e.* single HCC nodule ≤3 cm in diameter, no extrahepatic disease) (n = 61), and (iv) progressed HCC (*i.e.* multiple HCC nodules or single nodule >3 cm in diameter) (n = 165) (for detailed clinical characteristics see Table 1 and the Supplemental material). To demonstrate the discriminative capacity of serum metabolome profiling during hepatocarcinogenesis, we first aimed at mapping alterations in serum metabolome profiles (623 metabolites) across CLD, cirrhosis, initial HCC, and progressed HCC (N = 406). We found significant deregulation of several metabolite classes across groups (Fig. 2 and Fig. S2), such as amino acids, cholesterol esters (CE), fatty acids, nucleobase-related metabolites, sphingolipids, vitamins, and cofactors. Findings were independent of liver function, sex, age, and etiology (see Supplementary results and Figs. S3 and S4 for more details). Regarding individual metabolites from top altered classes, aspartic acid, glutamic acid, several choline esters, xanthine, and hypoxanthine showed the most significantly altered abundance between the four groups (Fig. S5).

**Fig. 3:**
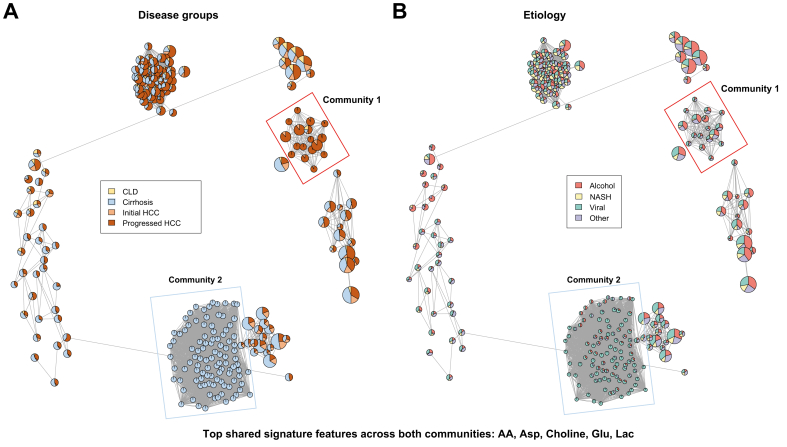
MoSBi analysis. Resulting bicluster network from biclustering analysis on metabolomics data. Biclusters (nodes) are colored by (A) disease group or (B) etiology. Network communities of interest are highlighted. AA, arachidonic acid; CLD, chronic liver disease; HCC, hepatocellular carcinoma; MoSBi, Molecular Signature identification using Biclustering; NASH, non-alcoholic steatohepatitis.

We next sought to validate deregulated metabolites by applying an unsupervised approach, specifically our ensemble approach “Molecular Signatures with Biclustering” (MoSBi)^22^ (Fig. 3). In contrast to conventional clustering, this unsupervised biclustering identifies clusters of samples and their characteristic metabolite signatures simultaneously. Two communities stood out specifically, as they were highly enriched with patients with either progressed HCC (community 1) or cirrhosis without HCC (community 2) (Fig. 3A). The progressed HCC community included all etiologies, whereas the cirrhosis-specific communities were predominantly of viral and alcohol origin (Fig. 3B). Looking at the molecular signature that each bicluster of the two selected communities contains, we found that arachidonic acid (AA), aspartic acid, glutamic acid, lactic acid, and choline were shared top features across both communities, independently validating our previous, supervised analysis.

Together, these findings indicate that specific metabolome alterations, which occur during the transition from CLD to HCC, are detectable in the blood of patients. Importantly, findings are independent of liver function and etiology of liver disease.

### Aspartic acid, glutamic acid, taurine, and hypoxanthine are altered during malignant transformation

**Fig. 4:**
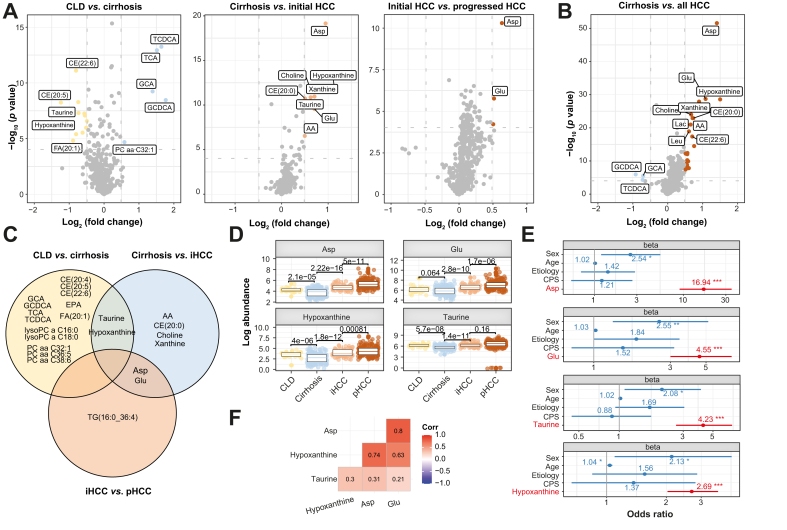
Differentially abundant metabolites across spectra of hepatocarcinogenesis. (A) Volcano plots displaying differential abundant metabolites between CLD (yellow) and cirrhosis (blue) (left panel), cirrhosis (blue) and initial HCC (light red) (middle panel), and initial HCC (light red) and progressed HCC (dark red) (right panel). (B) Volcano plot displaying differential abundant metabolites between cirrhosis (blue) and all HCC (dark red). (C) Venn diagram with differentially abundant metabolites by comparison. (D) Box plot for aspartic acid, glutamic acid, hypoxanthine, and taurine. (E) Ordinal logistic regression model, including clinical variables sex, age, etiology, and candidate metabolites. (F) Correlation matrix for top metabolites with Pearson correlation coefficient (all *p* <0.05). Full annotation for volcano plots is provided in Fig. S6. ∗*p* <0.05, ∗∗*p* <0.01, ∗∗∗*p* <0.001. Student’s *t* test, Wald test. AA, arachidonic acid; CE, cholesterol esters; CLD, chronic liver disease; CPS, Child-Pugh Score; GCA, glycocholic acid; GCDCA, glycochenodeoxycholic acid; HCC, hepatocellular carcinoma; iCCA, intrahepatic cholangiocarcinoma; PC, phosphatidylcholines; pHCC, progressed HCC; TCA, trichloroacetic acid; TCDCA, taurochenodeoxycholic acid; TG, triacylglycerols.

To obtain a more detailed understanding of which individual metabolites are most significantly deregulated during each step of hepatocarcinogenesis, we computed the differential abundance of all metabolites across the four groups (CLD, cirrhosis, initial HCC, and progressed HCC) (Fig. 4A and B, Figs. S6A–C, and Supplementary results). A total of 23 unique metabolites were significantly altered across all comparisons (all with at least 40% differential abundance and FDR <0.05). Of these, some were repeatedly significantly abundant between comparisons, namely, hypoxanthine and taurine between CLD *vs*. cirrhosis and cirrhosis *vs*. initial HCC, and aspartic acid and glutamic acid between cirrhosis *vs*. initial HCC and initial HCC *vs*. progressed HCC (Fig. 4C and D).

Next, we built an ordinal logistic regression model including clinical variables and the repeatedly differentially abundant metabolites aspartic acid, glutamic acid, taurine, and hypoxanthine to determine the predictive power of our candidates and to rule out relevant confounding by clinical characteristics across groups (Table 1). Multivariate models including sex, age, etiology, and liver function according to Child–Pugh stage, each with one of the metabolites, revealed significant predictive power for all four candidates (odds ratios [OR] between 2.69 and 16.94, all *p* <10^-8^) and sex (OR between 2.08 and 2.55, all *p* <0.05) to discriminate the four groups (Fig. 4E and Fig. S7). We observed a strong correlation between the amino acids aspartic acid and glutamic acid (Pearson’s correlation coefficient r = 0.8, *p* <0.05) and aspartic acid and hypoxanthine (r = 0.74, *p* < 0.05) (Fig. 4F).

Taken together, we identified aspartic acid, glutamic acid, hypoxanthine, and taurine as individual differentially abundant metabolites during different steps of HCC initiation, all independent of sex, age, and liver function.

### Ten-metabolite blood-based signature for early HCC detection

Based on these previous findings that significant alterations of individual serum metabolites during hepatocarcinogenesis are readily detectable in the serum, we next sought to test the predictive capacity of serum metabolomic profiling to discriminate between patients with and without HCC. For this, we performed a phase II biomarker case–control study following the recommendations for biomarker development of early cancer detection.^6^^,^^24^ We included 149 patients with cirrhosis without HCC and 226 patients with HCC of our “serum metabolome identification cohort,” including 181 patients with early-stage BCLC 0/A-HCC (80% of cases), representing the ideal target population. The clinical characteristics of the groups are presented in Tables S2 and S3. The individual performance of our four candidates (aspartic acid, glutamic acid, hypoxanthine, and taurine) to identify BCLC 0/A-HCC yielded an ROC–AUC between 80.5% and 90.8%, whereas serum alpha fetoprotein (AFP) alone yielded only an AUC of 77.2% (Table S4).

**Fig. 5:**
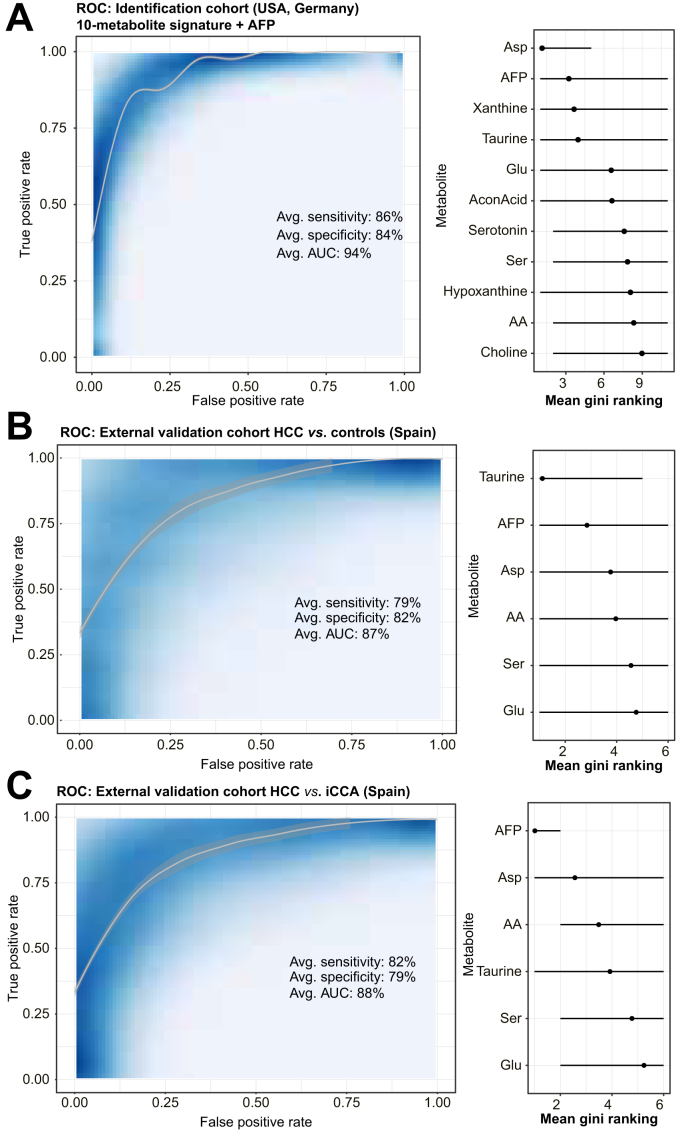
Biomarker analysis. Average area under the ROC curve (AUC, left panel) with indicated AUC, sensitivity, and specificity for a random forest classification model (internal cross-validation with 1,000 iterations) including top 10 metabolites plus AFP with respective candidates based on mean gini ranking (right panel). (A) “Serum metabolome identification cohort”: cirrhosis (n = 149) *vs*. early-stage HCC (BCLC 0/A, n = 181). (B) Spanish external validation cohort: controls (n = 35) *vs*. HCC (n = 32). (C) Spanish external validation cohort: HCC (n = 32) *vs*. iCCA (n = 35). (B and C: signature limited to five of 10 available metabolites in the Spanish dataset plus AFP). AA, arachidonic acid; AFP, alpha fetoprotein; BCLC, Barcelona Clinic for Liver Cancer; HCC, hepatocellular carcinoma; iCCA, intrahepatic cholangiocarcinoma; ROC, receiver operating characteristic.

To build a composite model with the most significant features to discriminate cases and controls, we created a random forest classifier using all metabolites and performed internal cross-validation with 1,000 iterations, randomly splitting the cohort into 70% training and 30% testing (Fig. S8). Importantly, the top 10 candidates based on mean gini ranking (aspartic acid, xanthine, taurine, glutamic acid, acon acid, serotonin, serine, hypoxanthine, AA, and choline) resembled similar features that were previously identified by unsupervised biclustering using our MoSBi approach (Fig. 3) and differential abundance analysis (Fig. 4C). Moreover, abundance of candidate metabolites hardly correlated with serum AFP levels (Fig. S9). We therefore built a composite model around the top 10 candidate metabolites plus AFP and trained it only on cases with BCLC 0/A-HCC (n = 181) and controls with cirrhosis (n = 149), yielding an average AUC of 94% with a sensitivity of 86% and a specificity of 84% in the testing dataset after internal cross-validation (data randomly split 1,000 times into 70/30 training and testing sets) (Fig. 5A and Fig. S10). Performance of the metabolite-based signature with and without AFP for different subgroups (sex, etiology, and cirrhotic/non-cirrhotic background) is displayed in Tables S5 and S6.

In an independent external validation cohort (n = 102; Table S7), where fewer metabolites were tested, a signature comprising five out of our 10 available metabolite candidates (aspartic acid, glutamic acid, taurine, serine, and AA) plus AFP, achieved an AUC of 87%, with a sensitivity of 79% and a specificity of 82% to discriminate HCC and controls (Fig. 5B and Fig. S10). In this cohort, the signature was also able to differentiate between intrahepatic cholangiocarcinoma (iCCA) and HCC (AUC 88%, sensitivity 82%, specificity 79%; Fig. 5C and Fig. S10).

These findings suggest serum metabolomic profiling as a novel liquid biopsy strategy for early HCC detection.

### Key altered metabolomic pathways during hepatocarcinogenesis

Given the clinical utility of our previous findings, we sought to better understand systematic changes in metabolomic pathways during hepatocarcinogenesis and ultimately facilitate biological validation of our blood-based findings. For this, we performed a network analysis and an integrated pathway enrichment analysis of our serum metabolomic data. First, we created a functional lipid network using our LINEX^2^ approach.^23^ By visualizing metabolomic reactions between observed serum lipids of patients with HCC (n = 226) and cirrhosis (n = 149), the network highlights the already mentioned alterations of CE, triacylglycerol (TG), and phosphatidylcholine (PC) lipid classes (Fig. S11). For example, it also shows that strongly connected and polyunsaturated TG (*e.g.* TG(52:6) FDR = 0.0008, TG(55:6) FDR = 4.06 × 10^-6^) are highly increased in HCC (also see Supplementary results). This indicates the incorporation of polyunsaturated fatty acids, such as AA, in the lipidome. Altogether, we found alterations in the glycerolipid and glycerophospholipid metabolism, ether lipid metabolism, and fatty acid metabolism, specifically AA.

**Fig. 6:**
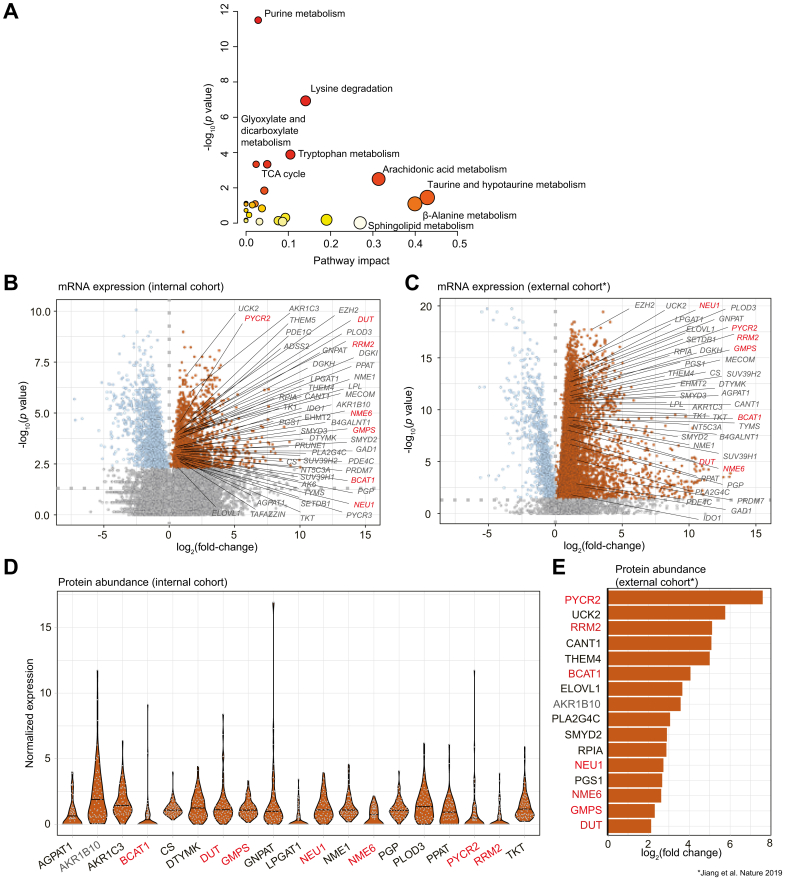
Metabolic pathway analysis. (A) Metabolic pathway analysis for “serum metabolome identification cohort” profiling indicating highly impacted pathways between cirrhosis (n = 149) and HCC (n = 226). (B, C) Differential gene expression analysis between primary HCC tumor and non-tumoral adjacent tissue from the internal (B) and public cohort^13^ (C). (D) Protein abundance in HCC tissue of the internal cohort. (E) Differentially abundant proteins in the public cohort.^13^ In (B–E), labeled are genes/proteins from mostly altered metabolic pathways identified in previous analysis (red: final candidates with differential gene expression and protein abundance across both cohorts; gray: remaining). Wilcoxon rank-sum test. HCC, hepatocellular carcinoma TCA, tricarboxylic acid.

In addition, we computed an integrated pathway enrichment analysis including pathway topology features, comparing cirrhosis and HCC serum metabolomes of our patients by applying the MetaboAnalyst 5.0 tool (developed by Xia Lab, Quebec, Canada).^25^^,^^26^ In fact, very strong pathway impact was observed in AA metabolism (impact 0.31, FDR = 1.43 × 10^-2^) (Fig. 6A), confirming results from our lipid network analysis (LINEX^2^),^23^ as well as in taurine and hypotaurine metabolism (impact 0.43, FDR = 1.19 × 10^-1^) and beta-alanine metabolism (impact 0.40, FDR = 1.53 × 10^-1^), confirming our other previous analysis. Moreover, lysine degradation and tryptophan metabolism were moderately affected (impact 0.14, FDR = 1.58 × 10^-6^, and impact 0.14, FDR = 1.17 × 10^-3^, respectively), whereas citrate cycle (tricarboxylic acid [TCA] cycle), purine metabolism, and glyoxylate and dicarboxylate metabolism were significantly less affected (impact 0.05, FDR = 2.50 × 10^-3^; impact 0.03, FDR = 8.44 × 10^-11^; and impact 0.02, FDR = 2.50 × 10^-3^, respectively) (Fig. 6A).

This is in line with our previous analysis, indicating alterations in global pathways of amino acid biosynthesis and nucleotide metabolism (Fig. 4).

### Biological validation of candidate pathways identifies actionable alterations

Finally, we sought to test whether alterations in candidate pathways (*e.g.* amino acid biosynthesis and nucleotide metabolism), which we repeatedly identified through various analysis of our serum metabolome datasets, could be orthogonally validated on gene expression and protein abundance levels in primary HCC tissue specimens from two independent datasets (for clinical data, see Table S8 and Jiang *et al.*^13^). In fact, differential gene expression analysis between HCC tumor and adjacent non-tumoral tissue in our internal German cohort (RNAseq available for n = 40 specimens) revealed significant enrichment (hypergeometric test) of genes associated with metabolic pathways that were identified by our serum metabolome analysis, that is, nucleotide metabolism (FDR = 7.0 × 10^-9^), including purine metabolism (FDR <0.05) and lysine degradation (*p* = 2.2 × 10^-4^). Biosynthesis of amino acids, glycerolipid and glycerophospholipid metabolism, and fatty acid elongation showed a trend towards significance (FDR <0.1). Among these pathways, 52 genes were significantly upregulated in tumor tissue on gene expression level (Fig. 6B), of which 19 genes were also detectable at the protein level in HCC tissue in our internal German cohort (proteomics available for n = 71 specimens) (Fig. 6D). Validation analysis in an external Chinese dataset^13^ confirmed 44 of our initial 52 upregulated genes in tumor tissue (RNAseq available for n = 70 specimens) (Fig. 6C), of which 16 were differentially upregulated in tumor tissue at the protein level (proteomics available for 199 specimens) (Fig. 6E).

Interestingly, seven candidate genes were upregulated in tumor tissue at both the gene and protein levels throughout both datasets, highlighting significant deregulation of their respective metabolic pathways in HCC. The candidates were DUT, GMPS, NME6, and RRM2 (all purine metabolism and/or nucleotide metabolism), BCAT1 and PYCR2 (both biosynthesis of amino acids), and NEU1 (sphingolipid metabolism).

**Fig. 7:**
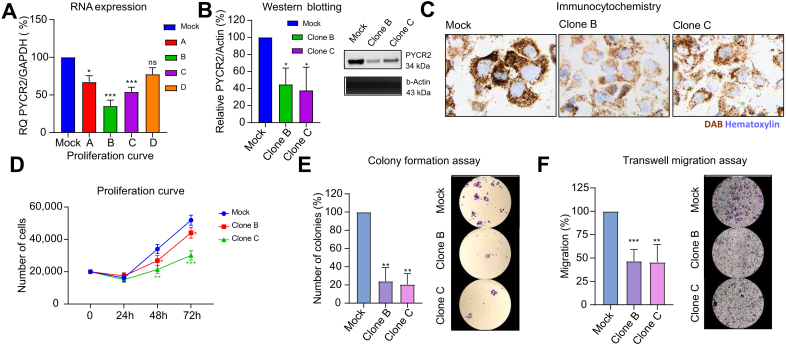
*In vitro* sh-RNA knockdown experiments targeting PYCR2. (A) RNA expression by RT-qPCR for clone selection. Protein expression of PYCR2 and housekeeper beta-actin by (B) Western blotting and (C) immunocytochemistry. (D) Proliferation curve. (E) Colony formation assay. (F) Transwell migration assay. Data are expressed as mean ± SD of at least three experiments. Representative images acquired at 4 × magnification. ∗*p* <0.05; ∗∗*p* <0.01; ∗∗∗*p* <0.001. Student’s *t* test. PYCR2, pyrroline-5-carboxylate reductase 2; sh-RNA, short-harpin RNA.

As an example, we further investigated the functional role of PYCR2 with short-harpin RNA (sh-RNA) knockdown experiments *in vitro*. Interference with PYCR2 expression, confirmed by RT-PCR, Western blotting, and immunocytochemistry, led to significantly decreased proliferation (-32% at 72 h), colony formation (-77%), and migration (-46%) in HCC cells (all *p* <0.01) (Fig. 7).

Together, these findings confirm a significant role of amino acid-, lipid- and nucleotide-related pathways during HCC initiation, including the identification of actionable candidates, such as PYCR2. As these were identified through our serum metabolomic profiling, the results underscore that alterations in the serum metabolome indicate deregulated pathways of hepatocarcinogenesis.

## Discussion

We conducted a global, multicenter analysis with 654 patients and 801 biospecimens from the USA, Germany, Spain, and China, including a publicly available dataset.^13^ By performing serum metabolomic profiling, we found alterations in amino acid-, lipid-, and nucleotide-related metabolism in patients across the spectrum of human hepatocarcinogenesis. A phase II biomarker case–control study yielded high accuracy for a metabolite-based signature from blood for early HCC detection. Finally, we validated key altered pathways at the gene expression and protein levels in primary HCC tissue and identified actionable candidates. These results demonstrate that serum metabolomic profiling captures significantly deregulated metabolism of HCC with direct clinical implications such as early detection and chemoprevention strategies.

The main clinical implication of our findings is our metabolite-based signature for early HCC detection from blood, which yielded an AUC of 94% with a sensitivity of 86% and a specificity of 84% to discriminate between patients with early-stage HCC (*i.e.* BCLC 0/A) and controls with cirrhosis. Of note, our identification cohort represents the ideal population for a case–control setting, which has been a frequent limitation for several studies in this field.^27^ In fact, the majority of HCC arises in patients with cirrhosis, with an annual incidence of 2–8%, rendering biannual ultrasound surveillance cost-effective in these patients.^6^^,^^14^ However, detection rates for early-stage HCC are only moderate according to a recent meta-analysis with >10,000 patients, with an average sensitivity as low as 63% and an average specificity of 84%.^5^ Moreover, ultrasound-based surveillance has several other limitations, including inter-operator variability and poor adherence.^28^^,^^29^ Several blood-based approaches have been investigated to date, including conventional tumor markers alone or in combination (*e.g.* AFP, AFP-L3, and DCP), algorithms (*e.g.* GALAD score), and liquid biopsy approaches, such as DNA methylation markers,^27^^,^^28^ small RNAs,^30^ and other metabolite-based tests.[10], [11], [12] Although current tumor marker-based tests or algorithms seem insufficient for HCC surveillance, DNA methylation profiling seems to have promising accuracy, and prospective clinical trials comparing different combinations against conventional ultrasound surveillance are currently ongoing.^28^ Considering its strong performance, our blood-based signature represents another promising approach for wide and easy implementation as an accurate tool for HCC surveillance pending further validation in prospective settings.

In the first part of our study, we found aspartic acid, glutamic acid, taurine (amino acids or amino acid-related metabolism), and hypoxanthine (nucleobase-related metabolism) among the most significantly deregulated individual serum metabolites across the spectrum of hepatocarcinogenesis. Deregulated uptake of amino acids, particularly glutamine, has been described as a hallmark of cancer metabolism^31^ via the TCA cycle, and nucleotide, and fatty acid biosynthesis.^32^ A recent study demonstrated a metabolic crosstalk between cancer-associated fibroblasts and cancer cells.^33^ In addition, genome instability and mutations are another hallmark of cancer.^8^ Tumor-promoting inflammation can lead to DNA damage via reactive oxidative and nitrogen species.^34^ Hypoxanthine, a purine derivative of deaminated adenine, indicates DNA damage and can lead to mutations,^35^ which supports our findings of increased levels during cancer evolution. In line with this, increased levels have also been observed in murine models of inflammatory colon cancer.^36^ In addition to amino acid and nucleotide-related metabolism, we observed significant changes in lipid metabolism during human hepatocarcinogenesis. We identified increased abundances of PC membrane lipids, with polyunsaturated PCs being most significantly altered, which is in accordance with a previous HCC study,^37^ suggesting an increased lipid remodeling and *de novo* synthesis. This is supported by a significant increase in choline in our patients with HCC.

Our results were confirmed using publicly available tools: our MoSBi algorithm^22^ for unsupervised biclustering, our LINEX^2^ software^23^ for lipid network analysis, and pathway enrichment analysis^26^ underscored that alterations in amino acid-, lipid-, and nucleotide-related pathways are systematically deregulated.

To orthogonally validate our initial findings regarding the most significantly altered serum metabolites and pathways, we evaluated mRNA expression and protein abundance in primary HCC tissue in two independent cohorts. Seven candidates were found to be upregulated in these pathways in primary HCC tissue at the mRNA and protein levels across both datasets (DUT, GMPS, NME6, RRM2, BCAT1, PYCR2, and NEU1). As an example, we selected PYCR2 (pyrroline-5-carboxylate reductase 2, biosynthesis of amino acids) for further functional experiments, as limited data were available in HCC. Our knockdown studies confirmed the relevance of PYCR2 in proliferation, colony formation, and migration, all key cancer features, underscoring its role as an actionable candidate during hepatocarcinogenesis. In addition, RRM2, GMPS (both purine metabolism and nucleotide metabolism), BCAT1 (biosynthesis of amino acids), and NEU1 (sphingolipid metabolism) have been previously reported in this context, for example, as an oncogene (RRM2^38^) or as modulators of cellular senescence (GMPS^39^) or carcinogenesis (BCAT1^40^ and NEU1^41^^,^^42^). Taken together, there is strong evidence that the majority of our candidates critically function in tumorigenesis and progression of HCC, thus underscoring their potential as drug targets, such as in the context of chemoprevention.

Our study has some limitations. First, the external validation cohort from Spain contained only five out of our 10 candidate metabolites. Despite this, the performance for HCC detection only slightly decreased and was still better than the recommended gold standard with ultrasound and AFP.^5^ Second, controls in the external validation cohort do not have cirrhosis, which does not represent the ideal target population. Nevertheless, given our multifaceted efforts to avoid overfitting in the “serum metabolome identification cohort,” such as large sample size, random forest models, and internal cross-validation, we certainly conclude that the reported performance in this cohort (representing an ideal population to test biomarkers for early HCC detection) is robust and valid, particularly considering that reported metrics (AUC 94%) were averaged from the test split dataset after internal cross-validation across 1,000 iterations of randomly splitting the data into training and test sets.

In summary, we identified significantly deregulated metabolites and metabolic pathways (amino acid-, lipid-, and nucleotide-related) by analyzing the serum metabolome of a large multicenter, global cohort and derived a highly accurate metabolite-based signature for early HCC detection from blood. Findings were independently validated, including primary HCC tissue and functional *in vitro* studies, confirming potentially actionable candidates during hepatocarcinogenesis (RRM2, GMPS, BCAT1, PYCR2, and NEU1) that might be useful for the design of personalized chemopreventive strategies in the future.

## Abbreviations

AA, arachidonic acid; AFP, alpha fetoprotein; BCAT1, branched-chain aminotransferases 1; BCLC, Barcelona Clinic for Liver Cancer; CE, cholesterol esters; CLD, chronic liver disease; DCP, des-γ-carboxy prothrombin; DUT, deoxyuridine triphosphatase; FDR, false discovery rate; FFPE, formalin-fixed paraffin-embedded; GMPS, guanine monophosphate synthase; HCC, hepatocellular carcinoma; iCCA, intrahepatic cholangiocarcinoma; LC-MS/MS, liquid chromatography–tandem mass spectrometry; LINEX, LipidNetworkExplorer; MoSBi, Molecular Signature identification using Biclustering; NEU1, neuraminidase 1; NME6, nucleoside diphosphate kinase 6; OR, odds ratio; PC, phosphatidylcholines; PR, precision–recall; PYCR2, pyrroline-5-carboxylate reductase 2; RNAseq, RNA sequencing; ROC, receiver operating characteristic; RRM2, ribonucleotide reductase regulatory subunit M2; TCA, tricarboxylic acid; TERT, telomerase reverse transcriptase; TG, triacylglycerols.

## Financial support

10.13039/100016285TDR and JKP are funded by the Bavarian State Ministry of Science and the Arts in the framework of the 10.13039/100024171Bavarian Research Institute for Digital Transformation, Germany (bidt, grant: LipiTUM). This publication is supported through state funds approved by the State Parliament of Baden-Württemberg for the Innovation Campus 10.13039/100018696Health + Life Science 10.13039/100027925Alliance Heidelberg Mannheim (10.13039/100016285TDR). TR is supported by the 10.13039/501100001659German Research Foundation, Germany (DFG, P06/KFO306 and INST 152/876-1 FUGG). JMB received funds from the European Union’s 10.13039/501100007601Horizon 2020 Research and Innovation Program (grant number 825510, ESCALON) Instituto de Salud Carlos III (10.13039/501100004587ISCIII), Spain (FORT23/00026, FIS PI18/01075, PI21/00922, and Miguel Servet Program CPII19/00008) co-funded by the 10.13039/501100000780European Union, “Fundación Científica de la Asociación Española Contra el Cáncer” (AECC Scientific Foundation; “Rare Cancer” grant 2017), 10.13039/100008538PSC Partners US, PSC Supports 10.13039/100007472UK (06119JB), and AMMF–The Cholangiocarcinoma Charity (EU/2019/AMMFt/001). JvF is supported by DFG, German 10.13039/501100002347Federal Ministry of Education and Research (10.13039/501100002347BMBF, 01EO2106, Germany), 10.13039/501100005972German Cancer Aid (10.13039/501100005972Deutsche Krebshilfe), Germany, and 10.13039/100008672Wilhelm Sander Foundation, Germany.

## Authors’ contributions

Study concept, design, and supervision: KS, JvF. Acquisition of funding: TR, AWL, SH, JMB, HW, JUM, AV, JKP, JvF. Acquisition of data: KS, MP, FP, JG, TWF, IL, PKH, CZ, DC, AW, TGL, MW, TR, HV, MM, DO, HS, MS, BS, RIRM, LI-S, JMB, HW, JUM, AV, JvF. Analysis and interpretation of data: KS, MP, TDR, LA, JB, HV, HW, JUM, AV, JKP, JvF. Drafting of the manuscript: KS, TDR, LA, DC, HV, JKP, JvF. Critical revision of the manuscript for important intellectual content: all authors.

## Data availability statement

RNAseq, proteomic, and metabolomic data are publicly available at EMBL-EBI BioStudies under accession number S-BSST1808.

## Conflicts of interest

KS has received advisory board fees from Roche, AstraZeneca, and MSD. CZ has received advisory board fees from Roche, MSD, and AstraZeneca. AW has received travel grants, honoraria, and/or advisory board fees from Bayer BMS, Sanofi, Roche, AstraZeneca, MSD, Merck KGaG, and Eisai. SH has received honoraria and/or consulting fees from Janssen Cilag, Ferring, AbbVie, Falk, Galapagos, Lilly, and BMS. BS has received financial support, fees, and/or grants from Astra Zeneca, BMS, Boston Scientific, Eisai, Incyte, MSD, Roche, Sanofi, and Sirtex Medical. JMB has received financial support, fees and/or grants from Albireo, Ipsen, Cymabay, AstraZeneca, Jazz Pharmaceuticals, Servier, Ikan Biotech, OWL Metabolomics, Incyte, Intercept, Advance, and Eisai. JUM has received grants, fees, and/or honoraria from AstraZeneca, MSD, Eisai, Ipsen, BMS, Incyte, and Roche. AV has received consulting fees from FirstWorld, Pioneering Medicine, and Genentech; and advisory board fees from BMS, Roche, Astra Zeneca, Eisai, and NGM Pharmaceuticals. He has stock options from Espervita and Atzeyo. JvF has received honoraria from Roche and AstraZeneca.

Please refer to the accompanying ICMJE disclosure forms for further details.

## Figures and Tables

**figs1:**