# Supplementary material for: Metabolomic liquid biopsy dynamics predict early-stage HCC and actionable candidates of human hepatocarcinogenesis
Source: JHEP Rep. 2025 Jan 30;7(5):101340. doi: 10.1016/j.jhepr.2025.101340 (PMC12023797; doi:10.1016/j.jhepr.2025.101340)
Supplement: Multimedia component 1 [file mmc1.pdf]

Metabolomic liquid biopsy dynamics predict early-stage HCC and actionable candidates of human hepatocarcinogenesis

Kornelius Schulze[1,2], Tim Daniel Rose[3,4], Lorenz Adlung[1,5,6], Manuela Peschka[7,8], Francesca Pagani[1], Joao Gorgulho[9,10], Thorben W. Fründt[1,2], Ismail Labgaa[11,12], Philipp K. Haber[11], Carolin Zimpel[13], Darko Castven[13], Arndt Weinmann[14], Teresa Garzia-Lezana[11], Moritz Waldmann[7], Thomas Renné[7,15,16], Hannah Voß[7], Manuela Moritz[7], Dorian Orlikowski[7], Hartmut Schlüter[7], Jan Baumbach[17], Myron Schwartz[18], Ansgar W. Lohse[1,2], Samuel Huber[1,2], Bruno Sangro[19,20], Rocio I.R. Macias[20,21], Laura Izquierdo-Sanchez[22], Jesus M. Banales[22,23,24], Henning Wege[1,2], Jens U. Marquardt[13,14], Augusto Villanueva[11,25], Josch Konstantin Pauling[3,26], Johann von Felden[1,2,*]

JHEP Reports **2025**. vol. 7 | 1–12

Check for updates

Background & Aims: Actionable candidates of hepatocarcinogenesis remain elusive, and tools for early detection are suboptimal. Our aim was to demonstrate that serum metabolome profiles reflect the initiation of hepatocellular carcinoma (HCC) and enable the identification of biomarkers for early HCC detection and actionable candidates for chemoprevention.

Methods: This global cohort study included 654 patients and 801 biospecimens. Following serum metabolome profiling across the spectrum of hepatocarcinogenesis, we conducted a phase II biomarker case–control study for early HCC detection. Findings were independently validated through *in silico* analysis, mRNA sequencing, and proteome profiling of primary HCC and non-tumoral tissue, and *in vitro* experiments.

Results: Aspartic acid, glutamic acid, taurine, and hypoxanthine were differentially abundant in the serum across chronic liver disease, cirrhosis, initial HCC, and progressed HCC, independent of sex, age, and etiology. In a phase II biomarker case–control study, a blood-based metabolite signature yielded an AUC of 94% to discriminate between patients with early-stage HCC and controls with cirrhosis, including independent validation. Unsupervised biclustering (MoSBi), lipid network analysis (LINEX^2), and pathway enrichment analysis confirmed alterations in amino acid-, lipid-, and nucleotide-related pathways. In tumor tissue, these pathways were significantly deregulated regarding gene and protein expression in two independent datasets, including actionable targets RRM2, GMPS, BCAT1, PYCR2, and NEU1. *In vitro* knockdown confirmed a functional role in proliferation and migration, as exemplified for PYCR2.

Conclusions: These findings demonstrate that serum metabolome profiling indicates deregulated metabolites and pathways during hepatocarcinogenesis. Our liquid biopsy approach accurately detects early-stage HCC outperforming currently recommended surveillance tools and facilitates identification of actionable candidates for chemoprevention.

© 2025 The Author(s). Published by Elsevier B.V. on behalf of European Association for the Study of the Liver (EASL). This is an open access article under the CC BY license (http://creativecommons.org/licenses/by/4.0/).

Introduction

Liver cancer mortality and incidence are steadily increasing.[1] Its most frequent form is hepatocellular carcinoma (HCC) at approximately 90%,[2] which typically arises in patients with chronic liver disease (CLD), particularly in the context of cirrhosis.[2,3] This stepwise process is mainly induced by chronic inflammation.[4] Despite this well-defined population at risk, recommended tools for early detection are suboptimal,[5,6] and mechanisms of hepatocarcinogenesis remain poorly understood.[4] Apart from TERT promoter mutations[4] and DNA methylation changes,[7] there is limited knowledge regarding early events and mechanisms that drive hepatocarcinogenesis.

Deregulated cellular metabolism is a hallmark of cancer,[8] and the liver functions as the major metabolic organ in humans. Therefore, aberrations of metabolic pathways in CLD and during HCC evolution are obvious. In fact, multiple alterations in liver cancer metabolism have been reported, and their clinical relevance has been emphasized.[9] Circulating metabolites have previously been identified to discriminate between HCC and controls; however, cohorts were small and/or limited to the context of fatty liver disease.[10–12]

This study addresses the two aforementioned clinical needs to develop more accurate tools for early HCC detection and to identify actionable targets during hepatocarcinogenesis. We hypothesize that serum metabolome profiles directly reflect the initiation of HCC and thus enable the identification of biomarkers for early HCC detection and actionable candidates for chemoprevention.

To test our hypothesis, we mapped alterations in serum metabolome profiles across the progressing stages of human hepatocarcinogenesis, including patients with CLD with and

* Corresponding author. Address: I. Department of Medicine, University Medical Center Hamburg-Eppendorf, Martinistr. 52, 20246 Hamburg, Germany. Phone: +49 40 7410-53910
E-mail address: j.von-felden@uke.de (J. von Felden).
https://doi.org/10.1016/j.jhepr.2025.101340

without cirrhosis, initial HCC, and progressed HCC. We identified significantly altered metabolites and associated pathways (*i.e.* amino acid-, lipid-, and nucleotide-related pathways) linked to stepwise transformation towards HCC. We developed a metabolite-based signature from blood, which accurately identified early-stage HCC. Finally, gene expression and protein abundance of key metabolic enzymes in primary HCC identified actionable candidates during cancer initiation, including functional knockdown experiments.

Altogether, we provide novel insights into metabolic deregulation of HCC initiation and introduce its clinical implications, such as early detection and chemoprevention.

Patients and methods

Patient enrollment and specimen collection

In this multicenter, global study, 654 patients and 801 biospecimens were analyzed (Fig. 1). A total of 553 patients were actively enrolled from six different centers across three countries: USA: Mount Sinai Hospital in New York City, NY, n = 226 (serum only); Germany: University Medical Center Hamburg-Eppendorf n = 187 (n = 106 serum only, n = 36 paired serum and tissue, and n = 45 tissue only) and University Medical Center Mainz n = 38 (serum only); and Spain (all serum only): University Medical Center San Sebastian n = 52, University Medical Center Salamanca (National DNA Bank-Carlos III) n = 28, and University Medical Center Pamplona n = 23. In addition, 101 patients were accessed from an online dataset from China (tissue data only).[13] The study was conducted in accordance with both the Declarations of Helsinki and Istanbul. All research was approved by the local ethics committee (New York: HS-15-00540, Hamburg: PV-3578, Mainz: 837.199.10, San Sebastian: PI2019116, Salamanca: 21102016, and Pamplona: 2017.012), and written consent was given in writing by all participants.

Diagnosis of cirrhosis and/or HCC was made according to clinical guidelines.[14–16] Patients with concurrent malignancies were excluded.

Serum samples from Germany and the USA were combined into the "serum metabolome identification cohort" (N = 406) and grouped into patients with (i) CLD without cirrhosis (n = 31), (ii) CLD with cirrhosis (n = 149), (iii) initial HCC (*i.e.* single HCC nodule ≤3 cm in diameter, no extrahepatic disease) (n = 61), and (iv) progressed HCC (*i.e.* multiple HCC nodules or single nodule >3 cm in diameter) (n = 165) (for detailed clinical characteristics, see Table 1). The threshold of 3 cm was chosen to balance between biological (risk of occult metastasis[17,18]) and statistical (to obtain more equal group sizes) concerns. All

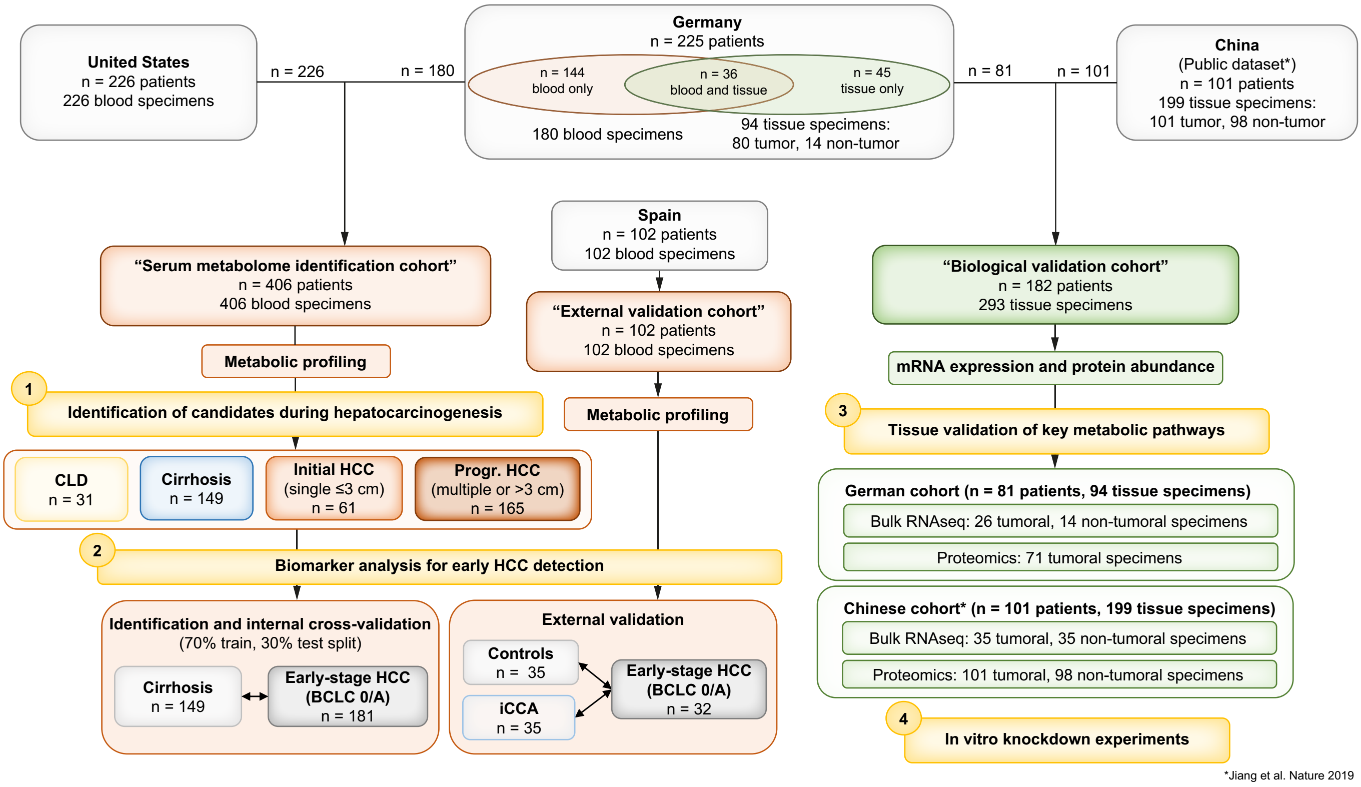

Fig. 1. Study overview. Outline of cohort distribution and experimental workflow. A total of 406 blood specimens from the USA and Germany were combined into the "serum metabolome identification cohort" for the identification of candidates during hepatocarcinogenesis (1) and a subset used for the biomarker analysis for early HCC detection (2). In addition, 102 blood specimens from Spain were used as an "external validation cohort" for the biomarker analysis (2). Biological validation of key metabolic pathways was conducted in 182 patients with 293 available tissue specimens (not all patients had paired tumor and non-tumoral tissue available) from the German (internal cohort) and Chinese cohorts by bulk mRNA-sequencing and proteomics analysis (3), alongside with *in vitro* studies (4). Some patients from the German cohort were included in both the "serum metabolome identification cohort" and the "biological validation cohort," according to availability of specimens. BCLC, Barcelona Clinic for Liver Cancer; CLD, chronic liver disease; HCC, hepatocellular carcinoma; iCCA, intrahepatic cholangiocarcinoma; RNAseq, RNA sequencing.

Table 1. Clinical characteristics of "serum metabolome identification cohort" (N = 406).

Characteristic	Overall (N = 406)*	CLD (n = 31)*	Cirrhosis (n = 149)*	Initial HCC (n= 61)*	Progressed HCC (n = 165)*	*p* value†
Sex						<0.001
Female	108 (30)	11 (35)	61 (41)	10 (16)	26 (21)	
Male	257 (70)	20 (65)	88 (59)	51 (84)	98 (79)	
Age (years)						0.056
<60	123 (34)	12 (39)	61 (41)	16 (26)	34 (27)	
≥60	242 (66)	19 (61)	88 (59)	45 (74)	90 (73)	
Diabetes	97 (37)	4 (22)	17 (38)	21 (36)	55 (39)	0.6
BMI (kg/m^2)	26.4 (23.1–30.9)	NA	26.6 (23.7–30.8)	26.0 (23.3–29.4)	26.4 (23.1–30.9)	>0.9
Etiology						
Alcohol	81 (20)	1 (3.2)	45 (30)	7 (11)	28 (17)	
NASH	52 (13)	0 (0)	17 (11)	13 (21)	22 (13)	
Viral	183 (45)	29 (94)	49 (33)	31 (51)	74 (45)	
Other	90 (22)	1 (3.2)	38 (26)	10 (16)	41 (25)	
Cirrhosis	290 (72)	0 (0)	149 (100)	47 (77)	94 (58)	<0.001
Child–Pugh stage‡						0.3
A	159 (71)	NA	90 (67)	26 (70)	43 (83)	
B	62 (28)	NA	42 (31)	11 (30)	9 (17)	
C	2 (0.9)	NA	2 (1.5)	0 (0)	0 (0)	
BCLC stage						<0.001
0	27 (12)	NA	NA	27 (44)	0 (0)	
A	154 (68)	NA	NA	34 (56)	120 (73)	
B	19 (8.4)	NA	NA	0 (0)	19 (12)	
C	25 (11)	NA	NA	0 (0)	25 (15)	
D	1 (0.4)	NA	NA	0 (0)	1 (0.6)	
AFP (ng/ml)	5 (3–25)	3 (2–4)	4 (2–5)	6 (4–27)	16 (5–304)	<0.001

AFP, alpha fetoprotein; BCLC, Barcelona Clinic for Liver Cancer; CLD, chronic liver disease; HCC, hepatocellular carcinoma; NA, not applicable; NASH, non-alcoholic steatohepatitis.
*Statistics are presented as n (%) or median (IQR).
†Statistical tests performed: Chi-square test of independence, Kruskal–Wallis test, and Fisher's exact test.
‡Only calculated for patients with cirrhosis.

blood specimens were collected during routine clinical management of patients and stored at -80 °C until further analysis.

For RNA and proteome analysis from tissue, 81 patients from the Hamburg cohort with 94 available tissue specimens (80 HCC tumoral tissue and 14 adjacent non-tumoral tissue specimens) were included. Of these patients, 36 were part of the serum metabolomics analysis with paired serum specimens (see below for details on sample collection and processing).

In addition, a publicly available RNA sequencing (RNAseq) and proteome dataset,[13] including 101 patients with 101 HCC tissue specimens and 98 paired adjacent non-tumoral tissue specimens, was used for external validation of our findings. Clinical data for the overall population of these patients were retrieved from the respective publication: 85% were male patients, predominantly with hepatitis B infection (98%). Furthermore, 82% had cirrhosis, and tumors were all stage 0 or A according to the Barcelona Clinic for Liver Cancer (BCLC) staging.[13]

Metabolomic profiling from serum specimens and pathway analysis

US and German serum samples were processed using the MxP® Quant 500 Kit (BIOCRATES Life Sciences AG, Innsbruck, Austria) according to the manufacturer's instructions. Spanish samples (external validation cohort) were analyzed in two ultra-high performance liquid chromatography (UHPLC)–time of flight–MS-based platforms, as previously described.[11]

Bulk mRNA sequencing from tissue specimen

For the Hamburg cohort, fresh tumoral and adjacent non-tumoral tissue specimens were collected during liver resection surgery for HCC. Total RNA was extracted from tissue using the Rneasy® Plus Mini Kit (Qiagen, Hilden, Germany) according to the manufacturer's instructions. After library construction, sequencing was performed using Illumina Novaseq 6000 (Illumina, Inc., San Diego, CA, USA).

Proteome profiling from tissue specimen

For the Hamburg cohort, protein was extracted from formalin-fixed paraffin-embedded (FFPE) tissue specimens with tryptic digestion as previously described.[19] Subsequently, 1 μg of peptides was subjected to liquid chromatography–tandem mass spectrometry (LC-MS/MS) measurements. Raw LC-MS/MS spectra were searched using the Sequest algorithm integrated into the Proteome Discoverer software (version 2.41.15, Thermo Fisher Scientific) against a reviewed human Swissprot database. Protein quantification was carried out using the Minora algorithm, implemented in Proteome Discoverer.[20]

Data analysis

Metabolomics data was $\log_2$ transformed, and the limma R package[21] was used to remove cohort batch effects while preserving patient groups (Fig. S1). Biclustering analysis was performed using the Molecular Signature identification using Biclustering (MoSBi) ensemble approach.[22]

For the phase II biomarker analysis, classifications were performed using random forest models. The data were randomly separated into training (70%) and test data (30%). This was repeated 1,000 times for each scenario. Average performance metrics (sensitivity, specificity, and AUC for receiver operating characteristic [ROC], and precision–recall [PR]) are reported for

the test data. Single candidate analysis was conducted using R package cutpointr.

The LipidNetworkExplorer LINEX^2 software[23] was used for the network analysis of lipids from the metabolome profiling. For this analysis, all lipids that were part of the metabolomics panel were included (Table S1).

Differentially expressed genes from bulk mRNA sequencing data were identified using normalized counts processed with the DESeq2 package. The Wilcoxon rank-sum test with *post hoc* correction for multiple testing was applied, similar to the dataset from Jiang *et al.*[13] Protein abundance was determined using within-gene and within-sample normalized values obtained by proteomics measurements (see Supplementary material). Comparison on a gene-by-gene level was performed against the dataset from Jiang *et al.*[13]

For descriptive statistics, continuous variables are reported as median and IQRs, and categorical variables are presented as counts and percentages. We used Fisher's exact test and Student's *t* test, the Kruskal–Wallis test, or ANOVA to compare differences between categorical and continuous variables. For correlation analysis, Pearson's correlation coefficient was used for continuous variables. Ordinal logistic regression was performed using the MASS R package. A *p* value less than 0.05 was considered statistically significant, and corrections for multiple comparisons were carried out where needed (false discovery rate [FDR] approach). All statistical and bioinformatic analyses were calculated in R studio (version 4.2).

Detailed information regarding metabolome profiling, mRNA sequencing, proteome profiling, and *in vitro* knockdown experiments is reported in the Supplementary material and Supplementary CTAT table.

Results

The study design is summarized in Fig. 1. Overall, we analyzed 654 patients and 801 biospecimens across four countries, including an external, publicly available dataset from China.[13]

Discriminative capacity of serum metabolome profiling during hepatocarcinogenesis

Serum samples from Germany and USA were combined into the "serum metabolome identification cohort" and grouped into patients with (i) CLD without cirrhosis (n = 31), (ii) cirrhosis (n = 149), (iii) initial HCC (*i.e.* single HCC nodule ≤3 cm in diameter, no extrahepatic disease) (n = 61), and (iv) progressed HCC (*i.e.* multiple HCC nodules or single nodule >3 cm in diameter) (n = 165) (for detailed clinical characteristics see Table 1 and the Supplemental material). To demonstrate the discriminative capacity of serum metabolome profiling during hepatocarcinogenesis, we first aimed at mapping alterations in serum metabolome profiles (623 metabolites) across CLD, cirrhosis, initial HCC, and progressed HCC (N = 406). We found significant deregulation of several metabolite classes across groups (Fig. 2 and Fig. S2), such as amino acids, cholesterol esters (CE), fatty acids, nucleobase-related metabolites, sphingolipids, vitamins, and cofactors. Findings were independent of liver function, sex, age, and etiology (see Supplementary results and Figs. S3 and S4 for more details). Regarding individual metabolites from top altered classes, aspartic acid, glutamic acid, several choline esters, xanthine, and hypoxanthine showed the most significantly altered abundance between the four groups (Fig. S5).

We next sought to validate deregulated metabolites by applying an unsupervised approach, specifically our ensemble approach "Molecular Signatures with Biclustering" (MoSBi)[22] (Fig. 3). In contrast to conventional clustering, this unsupervised biclustering identifies clusters of samples and their characteristic metabolite signatures simultaneously. Two communities stood out specifically, as they were highly enriched with patients with either progressed HCC (community 1) or cirrhosis without HCC (community 2) (Fig. 3A). The progressed HCC community included all etiologies, whereas the cirrhosis-specific communities were predominantly of viral and alcohol origin (Fig. 3B). Looking at the molecular signature that each bicluster of the two selected communities contains, we found that arachidonic acid (AA), aspartic acid, glutamic acid, lactic acid, and choline were shared top features across both communities, independently validating our previous, supervised analysis.

Together, these findings indicate that specific metabolome alterations, which occur during the transition from CLD to HCC, are detectable in the blood of patients. Importantly, findings are independent of liver function and etiology of liver disease.

Aspartic acid, glutamic acid, taurine, and hypoxanthine are altered during malignant transformation

To obtain a more detailed understanding of which individual metabolites are most significantly deregulated during each step of hepatocarcinogenesis, we computed the differential abundance of all metabolites across the four groups (CLD, cirrhosis, initial HCC, and progressed HCC) (Fig. 4A and B, Figs. S6A–C, and Supplementary results). A total of 23 unique metabolites were significantly altered across all comparisons (all with at least 40% differential abundance and FDR <0.05). Of these, some were repeatedly significantly abundant between comparisons, namely, hypoxanthine and taurine between CLD *vs*. cirrhosis and cirrhosis *vs*. initial HCC, and aspartic acid and glutamic acid between cirrhosis *vs*. initial HCC and initial HCC *vs*. progressed HCC (Fig. 4C and D).

Next, we built an ordinal logistic regression model including clinical variables and the repeatedly differentially abundant metabolites aspartic acid, glutamic acid, taurine, and hypoxanthine to determine the predictive power of our candidates and to rule out relevant confounding by clinical characteristics across groups (Table 1). Multivariate models including sex, age, etiology, and liver function according to Child–Pugh stage, each with one of the metabolites, revealed significant predictive power for all four candidates (odds ratios [OR] between 2.69 and 16.94, all $p < 10^{-8}$) and sex (OR between 2.08 and 2.55, all $p < 0.05$) to discriminate the four groups (Fig. 4E and Fig. S7). We observed a strong correlation between the amino acids aspartic acid and glutamic acid (Pearson's correlation coefficient r = 0.8, $p < 0.05$) and aspartic acid and hypoxanthine (r = 0.74, $p < 0.05$) (Fig. 4F).

Taken together, we identified aspartic acid, glutamic acid, hypoxanthine, and taurine as individual differentially abundant metabolites during different steps of HCC initiation, all independent of sex, age, and liver function.

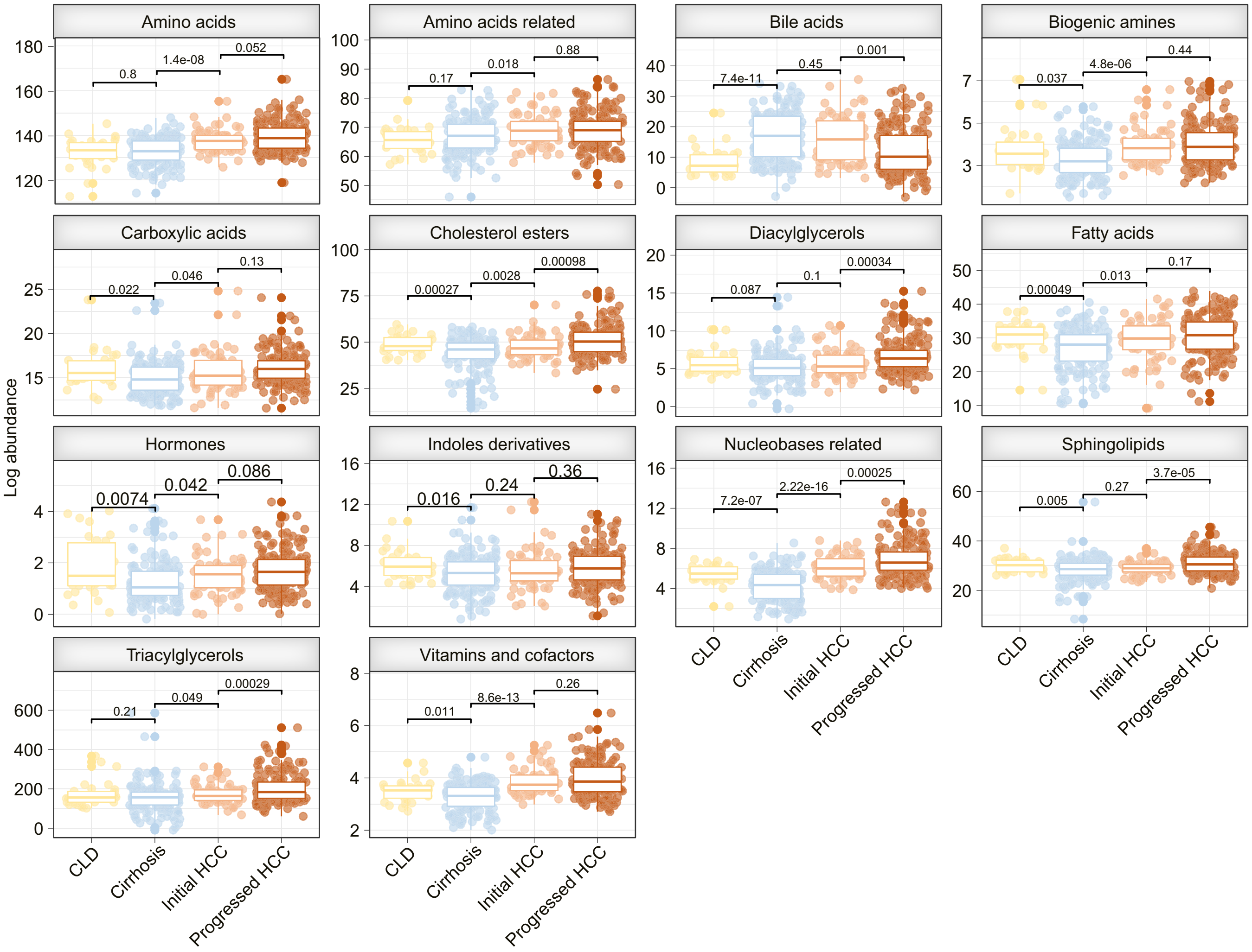

Fig. 2. Dynamic changes in serum metabolome. Abundance of significantly altered metabolite classes in sera across patients with CLD, cirrhosis, initial HCC, and progressed HCC. Display limited to metabolite classes with at least one significantly different group comparison for CLD *vs*. cirrhosis, cirrhosis *vs*. initial HCC, and initial HCC *vs*. progressed HCC. Student's *t* test. CLD, chronic liver disease; HCC, hepatocellular carcinoma.

Ten-metabolite blood-based signature for early HCC detection

Based on these previous findings that significant alterations of individual serum metabolites during hepatocarcinogenesis are readily detectable in the serum, we next sought to test the predictive capacity of serum metabolomic profiling to discriminate between patients with and without HCC. For this, we performed a phase II biomarker case–control study following the recommendations for biomarker development of early cancer detection.[6,24] We included 149 patients with cirrhosis without HCC and 226 patients with HCC of our "serum metabolome identification cohort," including 181 patients with early-stage BCLC 0/A-HCC (80% of cases), representing the ideal target population. The clinical characteristics of the groups are presented in Tables S2 and S3. The individual performance of our four candidates (aspartic acid, glutamic acid, hypoxanthine, and taurine) to identify BCLC 0/A-HCC yielded an ROC–AUC between 80.5% and 90.8%, whereas serum alpha fetoprotein (AFP) alone yielded only an AUC of 77.2% (Table S4).

To build a composite model with the most significant features to discriminate cases and controls, we created a random forest classifier using all metabolites and performed internal cross-validation with 1,000 iterations, randomly splitting the cohort into 70% training and 30% testing (Fig. S8). Importantly, the top 10 candidates based on mean gini ranking (aspartic acid, xanthine, taurine, glutamic acid, acon acid, serotonin, serine, hypoxanthine, AA, and choline) resembled similar features that were previously identified by unsupervised biclustering using our MoSBi approach (Fig. 3) and differential abundance analysis (Fig. 4C). Moreover, abundance of candidate metabolites hardly correlated with serum AFP levels (Fig. S9). We therefore built a composite model around the top 10 candidate metabolites plus AFP and trained it only on cases with BCLC 0/A-HCC (n = 181) and controls with cirrhosis (n = 149), yielding an average AUC of 94% with a sensitivity of 86% and a specificity of 84% in the testing dataset after internal cross-validation (data randomly split 1,000 times into 70/30 training and testing sets) (Fig. 5A and Fig. S10). Performance of the metabolite-based signature with and without AFP for

different subgroups (sex, etiology, and cirrhotic/non-cirrhotic background) is displayed in Tables S5 and S6.

In an independent external validation cohort (n = 102; Table S7), where fewer metabolites were tested, a signature comprising five out of our 10 available metabolite candidates (aspartic acid, glutamic acid, taurine, serine, and AA) plus AFP, achieved an AUC of 87%, with a sensitivity of 79% and a specificity of 82% to discriminate HCC and controls (Fig. 5B and Fig. S10). In this cohort, the signature was also able to differentiate between intrahepatic cholangiocarcinoma (iCCA) and HCC (AUC 88%, sensitivity 82%, specificity 79%; Fig. 5C and Fig. S10).

These findings suggest serum metabolomic profiling as a novel liquid biopsy strategy for early HCC detection.

Key altered metabolomic pathways during hepatocarcinogenesis

Given the clinical utility of our previous findings, we sought to better understand systematic changes in metabolomic pathways during hepatocarcinogenesis and ultimately facilitate biological validation of our blood-based findings. For this, we performed a network analysis and an integrated pathway enrichment analysis of our serum metabolomic data. First, we created a functional lipid network using our LINEX^2 approach.[23] By visualizing metabolomic reactions between observed serum lipids of patients with HCC (n = 226) and cirrhosis (n = 149), the network highlights the already mentioned alterations of CE, triacylglycerol (TG), and phosphatidylcholine (PC) lipid classes (Fig. S11). For example, it also shows that strongly connected and polyunsaturated TG (*e.g.* TG(52:6) FDR = 0.0008, TG(55:6) FDR = 4.06 × 10^{-6}) are highly increased in HCC (also see Supplementary results). This indicates the incorporation of polyunsaturated fatty acids, such as AA, in the lipidome. Altogether, we found alterations in the glycerolipid and glycerophospholipid metabolism, ether lipid metabolism, and fatty acid metabolism, specifically AA.

In addition, we computed an integrated pathway enrichment analysis including pathway topology features, comparing cirrhosis and HCC serum metabolomes of our patients by applying the MetaboAnalyst 5.0 tool (developed by Xia Lab, Quebec, Canada).[25,26] In fact, very strong pathway impact was observed in AA metabolism (impact 0.31, FDR = 1.43 × 10^{-2}) (Fig. 6A), confirming results from our lipid network analysis (LINEX^2),[23] as well as in taurine and hypotaurine metabolism (impact 0.43, FDR = 1.19 × 10^{-1}) and beta-alanine metabolism (impact 0.40, FDR = 1.53 × 10^{-1}), confirming our other previous analysis. Moreover, lysine degradation and tryptophan metabolism were moderately affected (impact 0.14, FDR = 1.58 × 10^{-6}, and impact 0.14, FDR = 1.17 × 10^{-3}, respectively), whereas citrate cycle (tricarboxylic acid [TCA] cycle), purine metabolism, and glyoxylate and dicarboxylate metabolism were significantly less affected (impact 0.05, FDR = 2.50 × 10^{-3}; impact 0.03, FDR = 8.44 × 10^{-11}; and impact 0.02, FDR = 2.50 × 10^{-3}, respectively) (Fig. 6A).

This is in line with our previous analysis, indicating alterations in global pathways of amino acid biosynthesis and nucleotide metabolism (Fig. 4).

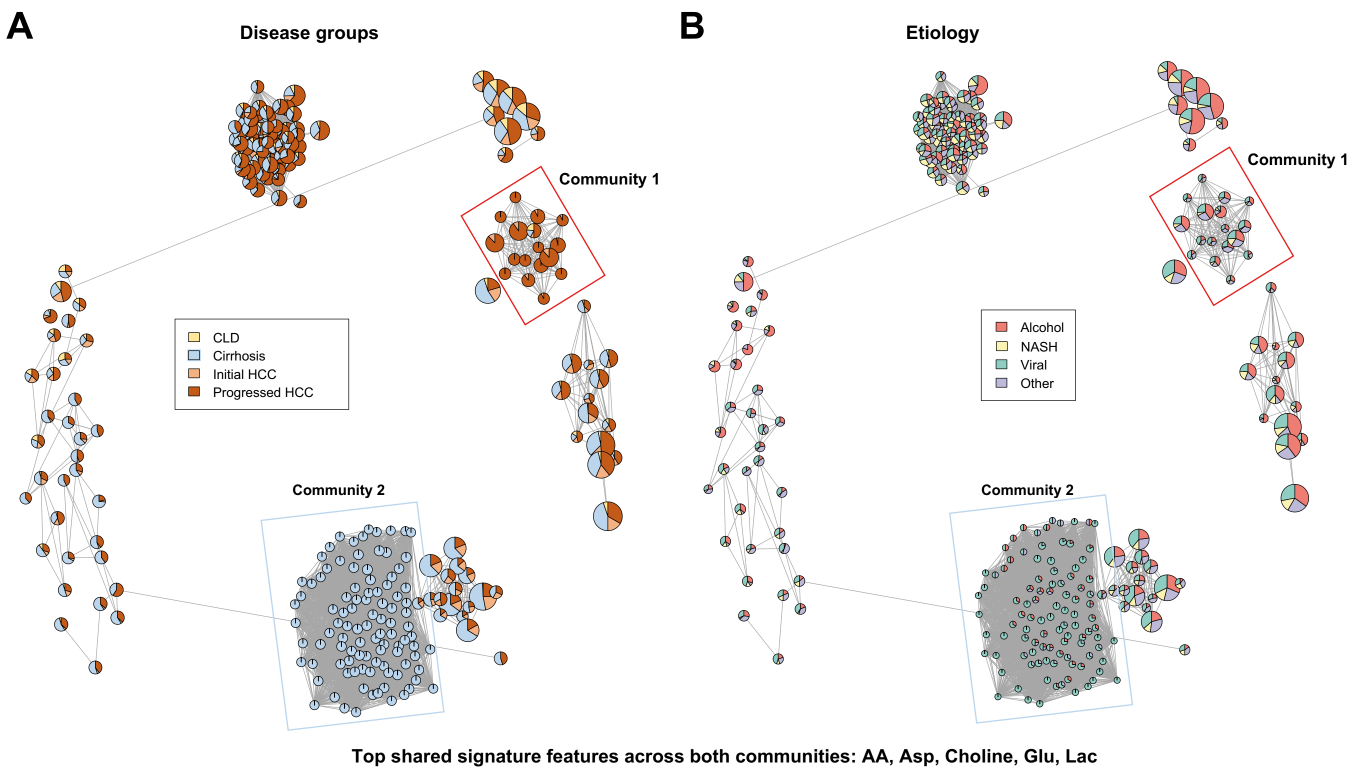

Fig. 3. MoSBi analysis. Resulting bicluster network from biclustering analysis on metabolomics data. Biclusters (nodes) are colored by (A) disease group or (B) etiology. Network communities of interest are highlighted. AA, arachidonic acid; CLD, chronic liver disease; HCC, hepatocellular carcinoma; MoSBi, Molecular Signature identification using Biclustering; NASH, non-alcoholic steatohepatitis.

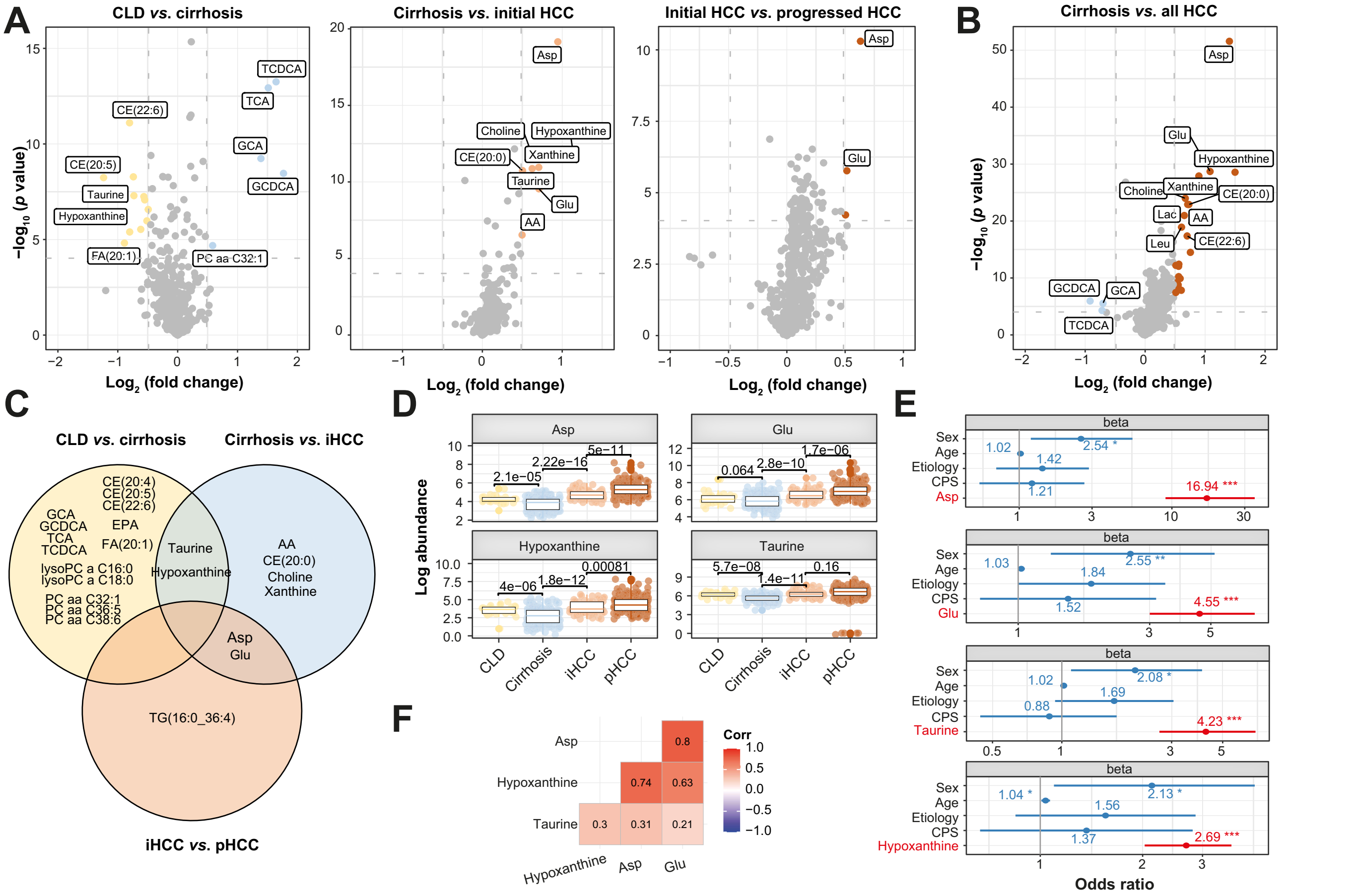

Fig. 4. Differentially abundant metabolites across spectra of hepatocarcinogenesis. (A) Volcano plots displaying differential abundant metabolites between CLD (yellow) and cirrhosis (blue) (left panel), cirrhosis (blue) and initial HCC (light red) (middle panel), and initial HCC (light red) and progressed HCC (dark red) (right panel). (B) Volcano plot displaying differential abundant metabolites between cirrhosis (blue) and all HCC (dark red). (C) Venn diagram with differentially abundant metabolites by comparison. (D) Box plot for aspartic acid, glutamic acid, hypoxanthine, and taurine. (E) Ordinal logistic regression model, including clinical variables sex, age, etiology, and candidate metabolites. (F) Correlation matrix for top metabolites with Pearson correlation coefficient (all p <0.05). Full annotation for volcano plots is provided in Fig. S6. *p <0.05, **p <0.01, ***p <0.001. Student's t test, Wald test. AA, arachidonic acid; CE, cholesterol esters; CLD, chronic liver disease; CPS, Child-Pugh Score; GCA, glycocholic acid; GCDCA, glycochenodeoxycholic acid; HCC, hepatocellular carcinoma; iCCA, intrahepatic cholangiocarcinoma; PC, phosphatidylcholines; pHCC, progressed HCC; TCA, trichloroacetic acid; TCDCA, taurochenodeoxycholic acid; TG, triacylglycerols.

Biological validation of candidate pathways identifies actionable alterations

Finally, we sought to test whether alterations in candidate pathways (*e.g.* amino acid biosynthesis and nucleotide metabolism), which we repeatedly identified through various analysis of our serum metabolome datasets, could be orthogonally validated on gene expression and protein abundance levels in primary HCC tissue specimens from two independent datasets (for clinical data, see Table S8 and Jiang *et al.*[13]). In fact, differential gene expression analysis between HCC tumor and adjacent non-tumoral tissue in our internal German cohort (RNAseq available for n = 40 specimens) revealed significant enrichment (hypergeometric test) of genes associated with metabolic pathways that were identified by our serum metabolome analysis, that is, nucleotide metabolism (FDR = 7.0×10^{-9}), including purine metabolism (FDR <0.05) and lysine degradation ($p = 2.2 \times 10^{-4}$). Biosynthesis of amino acids, glycerolipid and glycerophospholipid metabolism, and fatty acid elongation showed a trend towards significance (FDR <0.1). Among these pathways, 52 genes were significantly upregulated in tumor tissue on gene expression level (Fig. 6B), of which 19 genes were also detectable at the protein level in HCC tissue in our internal German cohort (proteomics available for n = 71 specimens) (Fig. 6D). Validation analysis in an external Chinese dataset[13] confirmed 44 of our initial 52 upregulated genes in tumor tissue (RNAseq available for n = 70 specimens) (Fig. 6C), of which 16 were differentially upregulated in tumor tissue at the protein level (proteomics available for 199 specimens) (Fig. 6E).

Interestingly, seven candidate genes were upregulated in tumor tissue at both the gene and protein levels throughout both datasets, highlighting significant deregulation of their respective metabolic pathways in HCC. The candidates were DUT, GMPS, NME6, and RRM2 (all purine metabolism and/or nucleotide metabolism), BCAT1 and PYCR2 (both biosynthesis of amino acids), and NEU1 (sphingolipid metabolism).

As an example, we further investigated the functional role of PYCR2 with short-harpin RNA (sh-RNA) knockdown experiments *in vitro*. Interference with PYCR2 expression, confirmed by RT-PCR, Western blotting, and immunocytochemistry, led

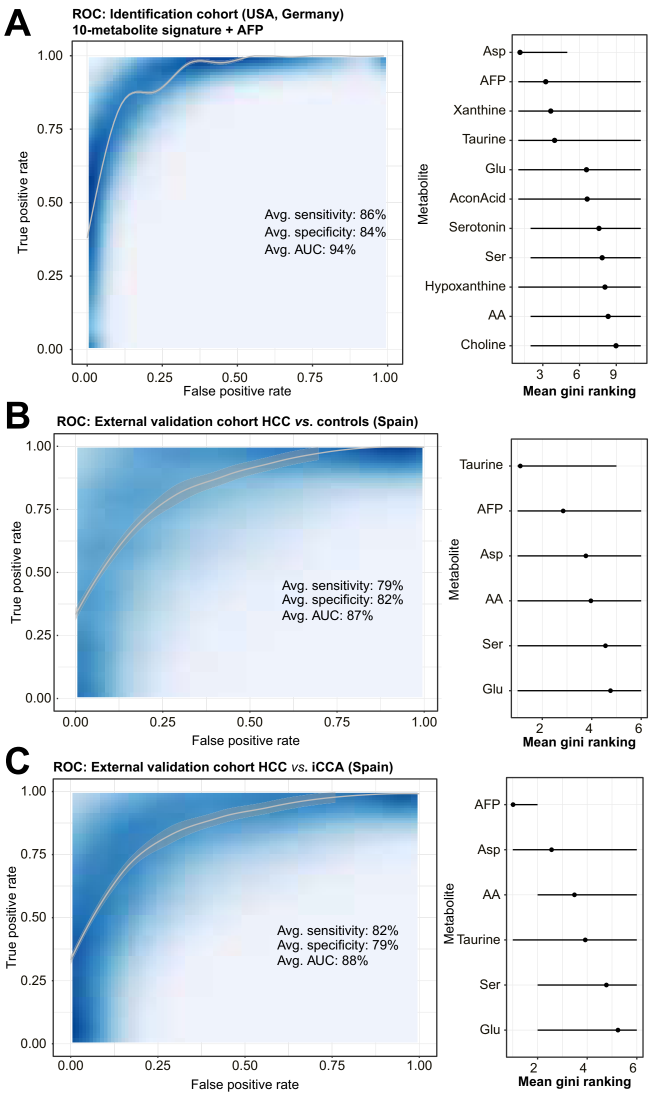

Fig. 5. Biomarker analysis. Average area under the ROC curve (AUC, left panel) with indicated AUC, sensitivity, and specificity for a random forest classification model (internal cross-validation with 1,000 iterations) including top 10 metabolites plus AFP with respective candidates based on mean gini ranking (right panel). (A) "Serum metabolome identification cohort": cirrhosis (n = 149) *vs*. early-stage HCC (BCLC 0/A, n = 181). (B) Spanish external validation cohort: controls (n = 35) *vs*. HCC (n = 32). (C) Spanish external validation cohort: HCC (n = 32) *vs*. iCCA (n = 35). (B and C: signature limited to five of 10 available metabolites in the Spanish dataset plus AFP). AA, arachidonic acid; AFP, alpha fetoprotein; BCLC, Barcelona Clinic for Liver Cancer; HCC, hepatocellular carcinoma; iCCA, intrahepatic cholangiocarcinoma; ROC, receiver operating characteristic.

to significantly decreased proliferation (-32% at 72 h), colony formation (-77%), and migration (-46%) in HCC cells (all p <0.01) (Fig. 7).

Together, these findings confirm a significant role of amino acid-, lipid- and nucleotide-related pathways during HCC initiation, including the identification of actionable candidates, such as PYCR2. As these were identified through our serum metabolomic profiling, the results underscore that alterations in the serum metabolome indicate deregulated pathways of hepatocarcinogenesis.

Discussion

We conducted a global, multicenter analysis with 654 patients and 801 biospecimens from the USA, Germany, Spain, and China, including a publicly available dataset.[13] By performing serum metabolomic profiling, we found alterations in amino acid-, lipid-, and nucleotide-related metabolism in patients across the spectrum of human hepatocarcinogenesis. A phase II biomarker case–control study yielded high accuracy for a metabolite-based signature from blood for early HCC detection. Finally, we validated key altered pathways at the gene expression and protein levels in primary HCC tissue and identified actionable candidates. These results demonstrate that serum metabolomic profiling captures significantly deregulated metabolism of HCC with direct clinical implications such as early detection and chemoprevention strategies.

The main clinical implication of our findings is our metabolite-based signature for early HCC detection from blood, which yielded an AUC of 94% with a sensitivity of 86% and a specificity of 84% to discriminate between patients with early-stage HCC (*i.e.* BCLC 0/A) and controls with cirrhosis. Of note, our identification cohort represents the ideal population for a case–control setting, which has been a frequent limitation for several studies in this field.[27] In fact, the majority of HCC arises in patients with cirrhosis, with an annual incidence of 2–8%, rendering biannual ultrasound surveillance cost-effective in these patients.[6,14] However, detection rates for early-stage HCC are only moderate according to a recent meta-analysis with >10,000 patients, with an average sensitivity as low as 63% and an average specificity of 84%.[5] Moreover, ultrasound-based surveillance has several other limitations, including inter-operator variability and poor adherence.[28,29] Several blood-based approaches have been investigated to date, including conventional tumor markers alone or in combination (*e.g.* AFP, AFP-L3, and DCP), algorithms (*e.g.* GALAD score), and liquid biopsy approaches, such as DNA methylation markers,[27,28] small RNAs,[30] and other metabolite-based tests.[10–12] Although current tumor marker-based tests or algorithms seem insufficient for HCC surveillance, DNA methylation profiling seems to have promising accuracy, and prospective clinical trials comparing different combinations against conventional ultrasound surveillance are currently ongoing.[28] Considering its strong performance, our blood-based signature represents another promising approach for wide and easy implementation as an accurate tool for HCC surveillance pending further validation in prospective settings.

In the first part of our study, we found aspartic acid, glutamic acid, taurine (amino acids or amino acid-related metabolism), and hypoxanthine (nucleobase-related metabolism) among the most significantly deregulated individual serum metabolites across the spectrum of hepatocarcinogenesis. Deregulated uptake of amino acids, particularly glutamine, has been described as a hallmark of cancer metabolism[31] via the TCA cycle, and nucleotide, and fatty acid biosynthesis.[32] A recent study demonstrated a metabolic crosstalk between cancer-associated fibroblasts and cancer cells.[33] In addition, genome instability and mutations are another hallmark of cancer.[8] Tumor-promoting inflammation can lead to DNA damage via reactive oxidative and nitrogen species.[34] Hypoxanthine, a purine derivative of deaminated adenine, indicates

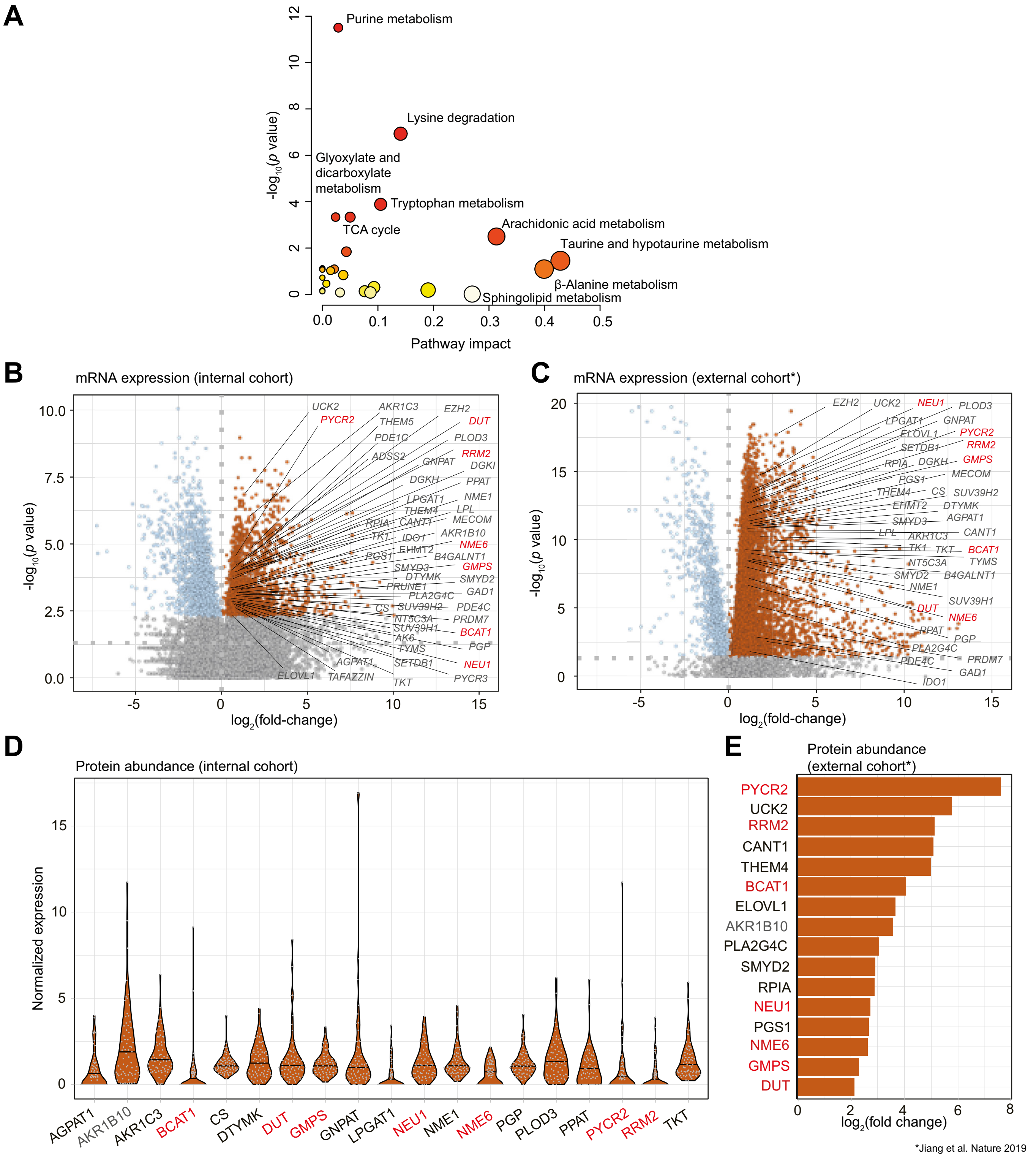

Fig. 6. Metabolic pathway analysis. (A) Metabolic pathway analysis for "serum metabolome identification cohort" profiling indicating highly impacted pathways between cirrhosis (n = 149) and HCC (n = 226). (B, C) Differential gene expression analysis between primary HCC tumor and non-tumoral adjacent tissue from the internal (B) and public cohort[13] (C). (D) Protein abundance in HCC tissue of the internal cohort. (E) Differentially abundant proteins in the public cohort.[13] In (B–E), labeled are genes/proteins from mostly altered metabolic pathways identified in previous analysis (red: final candidates with differential gene expression and protein abundance across both cohorts; gray: remaining). Wilcoxon rank-sum test. HCC, hepatocellular carcinoma TCA, tricarboxylic acid.

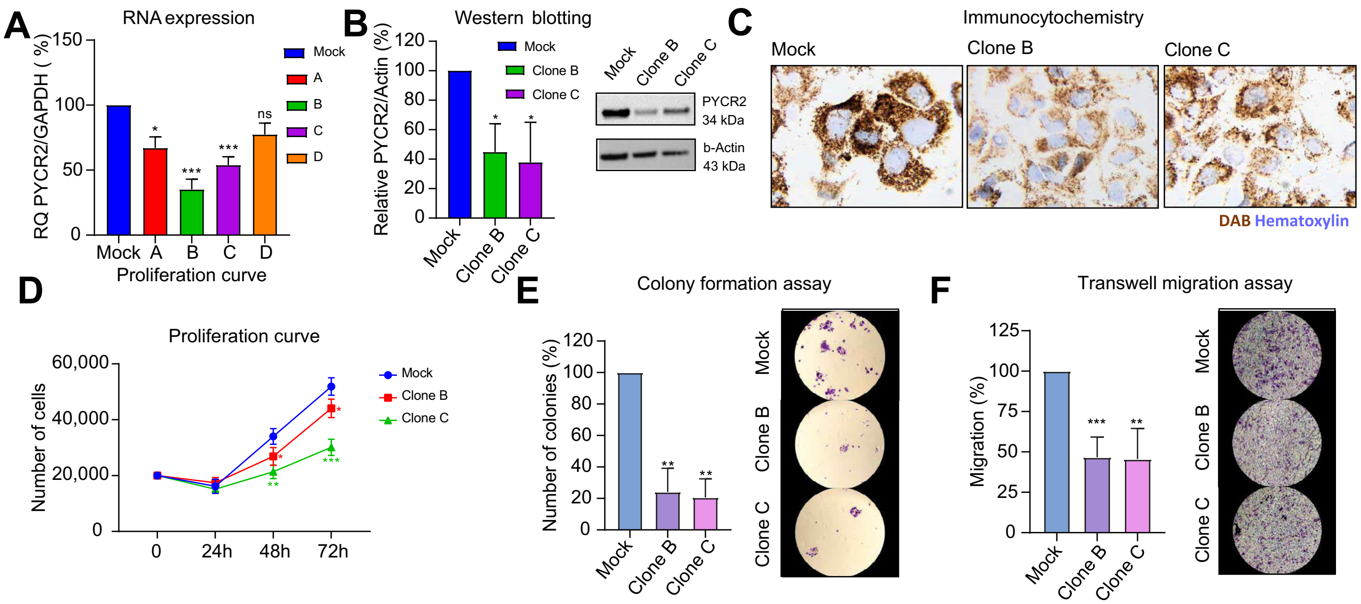

Fig. 7. *In vitro* sh-RNA knockdown experiments targeting PYCR2. (A) RNA expression by RT-qPCR for clone selection. Protein expression of PYCR2 and housekeeper beta-actin by (B) Western blotting and (C) immunocytochemistry. (D) Proliferation curve. (E) Colony formation assay. (F) Transwell migration assay. Data are expressed as mean ± SD of at least three experiments. Representative images acquired at 4 × magnification. **p* <0.05; ***p* <0.01; ****p* <0.001. Student's *t* test. PYCR2, pyrroline-5-carboxylate reductase 2; sh-RNA, short-harpin RNA.

DNA damage and can lead to mutations,[35] which supports our findings of increased levels during cancer evolution. In line with this, increased levels have also been observed in murine models of inflammatory colon cancer.[36] In addition to amino acid and nucleotide-related metabolism, we observed significant changes in lipid metabolism during human hepatocarcinogenesis. We identified increased abundances of PC membrane lipids, with polyunsaturated PCs being most significantly altered, which is in accordance with a previous HCC study,[37] suggesting an increased lipid remodeling and *de novo* synthesis. This is supported by a significant increase in choline in our patients with HCC.

Our results were confirmed using publicly available tools: our MoSBi algorithm[22] for unsupervised biclustering, our LINEX^2 software[23] for lipid network analysis, and pathway enrichment analysis[26] underscored that alterations in amino acid-, lipid-, and nucleotide-related pathways are systematically deregulated.

To orthogonally validate our initial findings regarding the most significantly altered serum metabolites and pathways, we evaluated mRNA expression and protein abundance in primary HCC tissue in two independent cohorts. Seven candidates were found to be upregulated in these pathways in primary HCC tissue at the mRNA and protein levels across both datasets (DUT, GMPS, NME6, RRM2, BCAT1, PYCR2, and NEU1). As an example, we selected PYCR2 (pyrroline-5-carboxylate reductase 2, biosynthesis of amino acids) for further functional experiments, as limited data were available in HCC. Our knockdown studies confirmed the relevance of PYCR2 in proliferation, colony formation, and migration, all key cancer features, underscoring its role as an actionable candidate during hepatocarcinogenesis. In addition, RRM2, GMPS (both purine metabolism and nucleotide metabolism), BCAT1 (biosynthesis of amino acids), and NEU1 (sphingolipid metabolism) have been previously reported in this context, for example, as an oncogene (RRM2[38]) or as modulators of cellular senescence (GMPS[39]) or carcinogenesis (BCAT1[40] and NEU1[41,42]). Taken together, there is strong evidence that the majority of our candidates critically function in tumorigenesis and progression of HCC, thus underscoring their potential as drug targets, such as in the context of chemoprevention.

Our study has some limitations. First, the external validation cohort from Spain contained only five out of our 10 candidate metabolites. Despite this, the performance for HCC detection only slightly decreased and was still better than the recommended gold standard with ultrasound and AFP.[5] Second, controls in the external validation cohort do not have cirrhosis, which does not represent the ideal target population. Nevertheless, given our multifaceted efforts to avoid overfitting in the "serum metabolome identification cohort," such as large sample size, random forest models, and internal cross-validation, we certainly conclude that the reported performance in this cohort (representing an ideal population to test biomarkers for early HCC detection) is robust and valid, particularly considering that reported metrics (AUC 94%) were averaged from the test split dataset after internal cross-validation across 1,000 iterations of randomly splitting the data into training and test sets.

In summary, we identified significantly deregulated metabolites and metabolic pathways (amino acid-, lipid-, and nucleotide-related) by analyzing the serum metabolome of a large multicenter, global cohort and derived a highly accurate metabolite-based signature for early HCC detection from blood. Findings were independently validated, including primary HCC tissue and functional *in vitro* studies, confirming potentially actionable candidates during hepatocarcinogenesis (RRM2, GMPS, BCAT1, PYCR2, and NEU1) that might be useful for the design of personalized chemopreventive strategies in the future.

Affiliations

[1]I. Department of Medicine, University Medical Center Hamburg-Eppendorf, Hamburg, Germany; [2]ERN-RARE-LIVER, Hamburg, Germany; [3]LipiTUM, Chair of Experimental Bioinformatics, TUM School of Life Sciences, Technical University of Munich, Munich, Germany; [4]Structural and Computational Biology Unit, European Molecular Biology Laboratory, Heidelberg, Germany; [5]Hamburg Center for Translational Immunology (HCTI), and Center for Biomedical AI (bAIome), Hamburg, Germany; [6]Mildred Scheel Cancer Career Center HaTriCS4, University Medical Center Hamburg-Eppendorf, Hamburg, Germany; [7]Institute of Clinical Chemistry and Laboratory Medicine, University Medical Center Hamburg-Eppendorf, Hamburg, Germany; [8]Newborn Screening and Metabolic Laboratory, Department of Pediatrics, University Medical Center Hamburg-Eppendorf, Hamburg, Germany; [9]Department of Oncology, Hematology and Bone Marrow Transplantation with Section of Pneumology, University Medical Centre Hamburg-Eppendorf, Hamburg, Germany; [10]University Cancer Center Hamburg–Hubertus Wald Tumorzentrum, University Medical Centre Hamburg-Eppendorf, Hamburg, Germany; [11]Division of Liver Diseases, Liver Cancer Program, Tisch Cancer Institute, Department of Medicine, Icahn School of Medicine at Mount Sinai, New York, NY, USA; [12]Department of Visceral Surgery, Lausanne University Hospital (CHUV), University of Lausanne (UNIL), Lausanne, Switzerland; [13]Department of Medicine I, University Medical Center Schleswig-Holstein-Campus Lübeck, Germany; [14]I. Department of Medicine, University Medical Center Mainz, Germany; [15]Irish Centre for Vascular Biology, School of Pharmacy and Biomolecular Sciences, Royal College of Surgeons in Ireland, Dublin, Ireland; [16]Center for Thrombosis and Hemostasis (CTH), Johannes Gutenberg University Medical Center, Mainz, Germany; [17]Chair of Computational Systems Biology, University of Hamburg, 22607 Hamburg, Germany; [18]Recanati Miller Transplant Institute, The Icahn School of Medicine at Mount Sinai Hospital, New York, NY, USA; [19]Liver Unit, Clinica Universidad de Navarra-IDISNA and CIBEREHD, Pamplona, Spain; [20]Center for the Study of Liver and Gastrointestinal Diseases (CIBEREHD), Carlos III National Institute of Health, Madrid, Spain; [21]Experimental Hepatology and Drug Targeting (HEVEPHARM), University of Salamanca, IBSAL, Salamanca, Spain; [22]Department of Liver and Gastrointestinal Diseases, Biogipuzkoa Health Research Institute–Donostia University Hospital, University of the Basque Country (UPV/EHU), CIBEREHD, Donostia-San Sebastian, Spain; [23]IKERBASQUE, Basque Foundation for Science, Bilbao, Spain; [24]Department of Biochemistry and Genetics, School of Sciences, University of Navarra, Pamplona, Spain; [25]Division of Hematology and Medical Oncology, Department of Medicine, Icahn School of Medicine at Mount Sinai, New York, NY, USA; [26]Institute for Clinical Chemistry and Laboratory Medicine, University Hospital and Faculty of Medicine Carl Gustav Carus, Dresden University of Technology, Dresden, Germany

Abbreviations

AA, arachidonic acid; AFP, alpha fetoprotein; BCAT1, branched-chain aminotransferases 1; BCLC, Barcelona Clinic for Liver Cancer; CE, cholesterol esters; CLD, chronic liver disease; DCP, des-γ-carboxy prothrombin; DUT, deoxyuridine triphosphatase; FDR, false discovery rate; FFPE, formalin-fixed paraffin-embedded; GMPS, guanine monophosphate synthase; HCC, hepatocellular carcinoma; iCCA, intrahepatic cholangiocarcinoma; LC-MS/MS, liquid chromatography–tandem mass spectrometry; LINEX, LipidNetworkExplorer; MoSBi, Molecular Signature identification using Biclustering; NEU1, neuraminidase 1; NME6, nucleoside diphosphate kinase 6; OR, odds ratio; PC, phosphatidylcholines; PR, precision–recall; PYCR2, pyrroline-5-carboxylate reductase 2; RNAseq, RNA sequencing; ROC, receiver operating characteristic; RRM2, ribonucleotide reductase regulatory subunit M2; TCA, tricarboxylic acid; TERT, telomerase reverse transcriptase; TG, triacylglycerols.

Financial support

TDR and JKP are funded by the Bavarian State Ministry of Science and the Arts in the framework of the Bavarian Research Institute for Digital Transformation, Germany (bidt, grant: LipiTUM). This publication is supported through state funds approved by the State Parliament of Baden-Württemberg for the Innovation Campus Health + Life Science Alliance Heidelberg Mannheim (TDR). TR is supported by the German Research Foundation, Germany (DFG, P06/KFO306 and INST 152/876-1 FUGG). JMB received funds from the European Union's Horizon 2020 Research and Innovation Program (grant number 825510, ESCALON) Instituto de Salud Carlos III (ISCIII), Spain (FORT23/00026, FIS PI18/01075, PI21/00922, and Miguel Servet Program CPII19/00008) co-funded by the European Union, "Fundación Científica de la Asociación Española Contra el Cáncer" (AECC Scientific Foundation; "Rare Cancer" grant 2017), PSC Partners US, PSC Supports UK (06119JB), and AMMF–The Cholangiocarcinoma Charity (EU/2019/AMMFt/001). JvF is supported by DFG, German Federal Ministry of Education and Research (BMBF, 01EO2106, Germany), German Cancer Aid (Deutsche Krebshilfe), Germany, and Wilhelm Sander Foundation, Germany.

Conflicts of interest

KS has received advisory board fees from Roche, AstraZeneca, and MSD. CZ has received advisory board fees from Roche, MSD, and AstraZeneca. AW has received travel grants, honoraria, and/or advisory board fees from Bayer BMS, Sanofi, Roche, AstraZeneca, MSD, Merck KGaG, and Eisai. SH has received honoraria and/or consulting fees from Janssen Cilag, Ferring, AbbVie, Falk, Galapagos, Lilly, and BMS. BS has received financial support, fees, and/or grants from Astra Zeneca, BMS, Boston Scientific, Eisai, Incyte, MSD, Roche, Sanofi, and Sirtex Medical. JMB has received financial support, fees and/or grants from Albireo, Ipsen, Cymabay, AstraZeneca, Jazz Pharmaceuticals, Servier, Ikan Biotech, OWL Metabolomics, Incyte, Intercept, Advance, and Eisai. JUM has received grants, fees, and/or honoraria from AstraZeneca, MSD, Eisai, Ipsen, BMS, Incyte, and Roche. AV has received consulting fees from FirstWorld, Pioneering Medicine, and Genentech; and advisory board fees from BMS, Roche, Astra Zeneca, Eisai, and NGM Pharmaceuticals. He has stock options from Espervita and Atzeyo. JvF has received honoraria from Roche and AstraZeneca.

Please refer to the accompanying ICMJE disclosure forms for further details.

Authors' contributions

Study concept, design, and supervision: KS, JvF. Acquisition of funding: TR, AWL, SH, JMB, HW, JUM, AV, JKP, JvF. Acquisition of data: KS, MP, FP, JG, TWF, IL, PKH, CZ, DC, AW, TGL, MW, TR, HV, MM, DO, HS, MS, BS, RIRM, LI-S, JMB, HW, JUM, AV, JvF. Analysis and interpretation of data: KS, MP, TDR, LA, JB, HV, HW, JUM, AV, JKP, JvF. Drafting of the manuscript: KS, TDR, LA, DC, HV, JKP, JvF. Critical revision of the manuscript for important intellectual content: all authors.

Data availability statement

RNAseq, proteomic, and metabolomic data are publicly available at EMBL-EBI BioStudies under accession number S-BSST1808.

Acknowledgement

We acknowledge financial support from the Open Access Publication Fund of UKE - Universitätsklinikum Hamburg-Eppendorf.

Supplementary data

Supplementary data to this article can be found online at https://doi.org/10.1016/j.jhepr.2025.101340.

References

Author names in bold designate shared co-first authorship

[1] Rumgay H, Arnold M, Ferlay J, et al. Global burden of primary liver cancer in 2020 and predictions to 2040. J Hepatol 2022;77:1598–1606.
[2] Villanueva A. Hepatocellular carcinoma. N Engl J Med 2019;380:1450–1462.
[3] Llovet JM, Kelley RK, Villanueva A, et al. Hepatocellular carcinoma. Nat Rev Dis Primers 2021;7:6.
[4] Craig AJ, von Felden J, Garcia-Lezana T, et al. Tumour evolution in hepatocellular carcinoma. Nat Rev Gastroenterol Hepatol 2020;17:139–152.
[5] **Tzartzeva K**, **Obi J**, Rich NE, et al. Surveillance imaging and alpha fetoprotein for early detection of hepatocellular carcinoma in patients with cirrhosis: a meta-analysis. Gastroenterology 2018;154:1706–17018. e1.
[6] **Singal AG**, **Sanduzzi-Zamparelli M**, Nahon P, et al. International Liver Cancer Association (ILCA) white paper on hepatocellular carcinoma risk stratification and surveillance. J Hepatol 2023;79:226–239.
[7] **Hernandez-Meza G**, **von Felden J**, Gonzalez-Kozlova EE, et al. DNA methylation profiling of human hepatocarcinogenesis. Hepatology 2021;74:183–199.
[8] Hanahan D. Hallmarks of cancer: new dimensions. Cancer Discov 2022;12:31–46.

[9] Satriano L, Lewinska M, Rodrigues PM, et al. Metabolic rearrangements in primary liver cancers: cause and consequences. Nat Rev Gastroenterol Hepatol 2019;16:748–766.
[10] Lu H, George J, Eslam M, et al. Discriminatory changes in circulating metabolites as a predictor of hepatocellular cancer in patients with metabolic (dysfunction) associated fatty liver disease. Liver Cancer 2023;12:19–31.
[11] Banales JM, Iñarrairaegui M, Arbelaiz A, et al. Serum metabolites as diagnostic biomarkers for cholangiocarcinoma, hepatocellular carcinoma, and primary sclerosing cholangitis. Hepatology 2019;70:547–562.
[12] Lewinska M, Santos-Laso A, Arretxe E, et al. The altered serum lipidome and its diagnostic potential for non-alcoholic fatty liver (NAFL)-associated hepatocellular carcinoma. EbioMedicine 2021;73:103661.
[13] Jiang Y, Sun A, Zhao Y, et al. Proteomics identifies new therapeutic targets of early-stage hepatocellular carcinoma. Nature 2019;567:257–261.
[14] European Association for the Study of the Liver. EASL Clinical Practice Guidelines: management of hepatocellular carcinoma. J Hepatol 2018;69:182–236.
[15] Singal AG, Llovet JM, Yarchoan M, et al. AASLD Practice Guidance on prevention, diagnosis, and treatment of hepatocellular carcinoma. Hepatology 2023;78:1922–1965.
[16] European Association for the Study of the Liver. EASL Clinical Practice Guidelines on non-invasive tests for evaluation of liver disease severity and prognosis—2021 update. J Hepatol 2021;75:659–689.
[17] Lee HA, Lee Y-S, Kim BK, et al. Change in the recurrence pattern and predictors over time after complete cure of hepatocellular carcinoma. Gut Liver 2021;15:420–429.
[18] Jung S-M, Kim JM, Choi G-S, et al. Characteristics of early recurrence after curative liver resection for solitary hepatocellular carcinoma. J Gastrointest Surg 2019;23:304–311.
[19] Hughes CS, Moggridge S, Müller T, et al. Single-pot, solid-phase-enhanced sample preparation for proteomics experiments. Nat Protoc 2019;14:68–85.
[20] Palomba A, Abbondio M, Fiorito G, et al. Comparative evaluation of MaxQuant and Proteome Discoverer MS1-based protein quantification tools. J Proteome Res 2021;20:3497–3507.
[21] Smyth GK. Linear models and empirical bayes methods for assessing differential expression in microarray experiments. Stat Appl Genet Mol Biol 2004;3:Article3.
[22] Rose TD, Bechtler T, Ciora O-A, et al. MoSBi: automated signature mining for molecular stratification and subtyping. Proc Natl Acad Sci U S A 2022;119:e2118210119.
[23] Rose TD, Köhler N, Falk L, et al. Lipid network and moiety analysis for revealing enzymatic dysregulation and mechanistic alterations from lipidomics data. Brief Bioinform 2023;24:bbac572.
[24] Pepe MS, Etzioni R, Feng Z, et al. Phases of biomarker development for early detection of cancer. J Natl Cancer Inst 2001;93:1054–1061.
[25] Pang Z, Chong J, Zhou G, et al. MetaboAnalyst 5.0: narrowing the gap between raw spectra and functional insights. Nucleic Acids Res 2021;49:W388–W396.
[26] Pang Z, Zhou G, Ewald J, et al. Using MetaboAnalyst 5.0 for LC–HRMS spectra processing, multi-omics integration and covariate adjustment of global metabolomics data. Nat Protoc 2022;17:1735–1761.
[27] von Felden J, Garcia-Lezana T, Schulze K, et al. Liquid biopsy in the clinical management of hepatocellular carcinoma. Gut 2020;69:2025–2034.
[28] Parikh ND, Tayob N, Singal AG. Blood-based biomarkers for hepatocellular carcinoma screening: approaching the end of the ultrasound era? J Hepatol 2023;78:207–216.
[29] Wolf E, Rich NE, Marrero JA, et al. Use of hepatocellular carcinoma surveillance in patients with cirrhosis: a systematic review and meta-analysis. Hepatology 2021;73:713–725.
[30] von Felden J, Garcia-Lezana T, Dogra N, et al. Unannotated small RNA clusters associated with circulating extracellular vesicles detect early stage liver cancer. Gut 2022;71. 2069–80.
[31] Pavlova NN, Thompson CB. The emerging hallmarks of cancer metabolism. Cell Metab 2016;23:27–47.
[32] Altman BJ, Stine ZE, Dang CV. From Krebs to clinic: glutamine metabolism to cancer therapy. Nat Rev Cancer 2016;16:619–634.
[33] Bertero T, Oldham WM, Grasset EM, et al. Tumor-stroma mechanics coordinate amino acid availability to sustain tumor growth and malignancy. Cel Metab 2019;29. 124–40.e10.
[34] Fioravanti A, Dotta F, Giordano A, et al. Crosstalk between microRNA and oxidative stress in physiology and pathology. Int J Mol Sci 2020; 21:1270.
[35] Kuraoka I. Diversity of Endonuclease V: from DNA repair to RNA editing. Biomolecules 2015;5:2194–2206.
[36] Mangerich A, Knutson CG, Parry NM, et al. Infection-induced colitis in mice causes dynamic and tissue-specific changes in stress response and DNA damage leading to colon cancer. Proc Natl Acad Sci U S A 2012;109: E1820–E1829.
[37] Hall Z, Chiarugi D, Charidemou E, et al. Lipid remodeling in hepatocyte proliferation and hepatocellular carcinoma. Hepatology 2021;73: 1028–1044.
[38] **Li Y**, **Fu W**, Geng Z, et al. A pan-cancer analysis of the oncogenic role of ribonucleotide reductase subunit M2 in human tumors. PeerJ 2022;10:e14432.
[39] Holzer K, Drucker E, Roessler S, et al. Proteomic analysis reveals GMP synthetase as p53 repression target in liver cancer. Am J Pathol 2017;187:228–235.
[40] **Wang J**, **Wang W**, Zhu F, et al. The role of branched chain amino acids metabolic disorders in tumorigenesis and progression. Biomed Pharmacother 2022;153:113390.
[41] Haxho F, Neufeld RJ, Szewczuk MR. Neuraminidase-1: a novel therapeutic target in multistage tumorigenesis. Oncotarget 2016;7:40860–40881.
[42] **Toussaint K**, **Appert-Collin A**, Morjani H, et al. Neuraminidase-1: a sialidase involved in the development of cancers and metabolic diseases. Cancers (Basel) 2022;14:4868.

Keywords: Liver cancer; Tumorigenesis; Prevention; Early detection; Surveillance; Metabolism.

Received 14 October 2024; received in revised form 16 January 2025; accepted 22 January 2025; Available online 30 January 2025

Journal of Hepatology, Volume *7*

Supplemental information

Metabolomic liquid biopsy dynamics predict early-stage HCC and actionable candidates of human hepatocarcinogenesis

Kornelius Schulze, Tim Daniel Rose, Lorenz Adlung, Manuela Peschka, Francesca Pagani, Joao Gorgulho, Thorben W. Fründt, Ismail Labgaa, Philipp K. Haber, Carolin Zimpel, Darko Castven, Arndt Weinmann, Teresa Garzia-Lezana, Moritz Waldmann, Thomas Renné, Hannah Voß, Manuela Moritz, Dorian Orlikowski, Hartmut Schlüter, Jan Baumbach, Myron Schwartz, Ansgar W. Lohse, Samuel Huber, Bruno Sangro, Rocio I.R. Macias, Laura Izquierdo-Sanchez, Jesus M. Banales, Henning Wege, Jens U. Marquardt, Augusto Villanueva, Josch Konstantin Pauling, and Johann von Felden

Metabolomic liquid biopsy dynamics predict early-stage HCC and actionable candidates of human hepatocarcinogenesis

Kornelius Schulze, Tim Daniel Rose, Lorenz Adlung, Manuela Peschka, Francesca Pagani, Joao Gorgulho, Thorben W. Fründt, Ismail Labgaa, Philipp K. Haber, Carolin Zimpel, Darko Castven, Arndt Weinmann, Teresa Garzia-Lezana, Moritz Waldmann, Thomas Renné, Hannah Voß, Manuela Moritz, Dorian Orlikowski, Hartmut Schlüter, Jan Baumbach, Myron Schwartz, Ansgar W. Lohse, Samuel Huber, Bruno Sangro, Rocio I.R. Macias, Laura Izquierdo-Sanchez, Jesus M. Banales, Henning Wege, Jens U. Marquardt, Augusto Villanueva, Josch Konstantin Pauling, Johann von Felden

Table of contents

Supplementary results……………………………………………………………………2

Supplementary methods…………………………………………………………………4

Supplementary references……………………………………………………………..10

Supplementary tables…………………………………………………………………..11

Supplementary figures…………………………………………………………………18

SUPPLEMENTARY RESULTS

Clinical parameters of the "serum metabolome identification cohort" (n=406) are displayed in **Table 1**. As expected, HCC patients were slightly older with a higher male to female ratio compared to controls ($p<0.001$). Viral etiology was the most prevalent risk factor for HCC in the total cohort (47%), and risk factors were distributed fairly equally across groups, except a predominance of viral etiology in the chronic liver disease group. Regarding parameters of liver function among patients with cirrhosis, distribution of Child-Pugh-Turcotte (CPT) stages was not significantly different between the groups ($p=0.3$). As expected, the serum tumor marker AFP was significantly higher in the progressed HCC group compared to the rest ($p<0.001$).

A more detailed stepwise analysis of metabolite classes across CLD, cirrhosis, initial HCC, and progressed HCC is displayed in **Suppl. Fig 2** for multiple group comparisons with ANOVA and **Fig. 2** with individual t-tests for group comparisons for metabolite classes with at least one significant comparison. A highly significant stepwise increase of metabolites from CLD towards progressed HCC was observed in amino acids ($p < 2*10^{-16}$) and amino acids-related metabolites ($p = 0.0022$). Other classes also showed significant changes but with a different pattern (e.g., bile acids ($p = 2*10^{-6}$), biogenic amines ($p = 3.1*10^{-10}$), carboxylic acids ($p = 4.1*10^{-7}$), cholesterol esters ($p = 6.3*10^{-9}$), fatty acids ($p = 6.6*10^{-6}$), hormones ($p = 9.9*10^{-7}$), nucleobases-related metabolites ($p < 2*10^{-16}$), triacylglycerols ($p = 4.7*10^{-8}$), and vitamins and cofactors ($p < 2*10^{-16}$). In general, the cirrhosis and progressed HCC groups showed higher variance in metabolomic abundances compared to the other groups, further highlighted by two subgroups within each cluster (**Suppl. Fig. 12**). However, dynamic changes across the spectrum of hepatocarcinogenesis were consistent when limiting the analysis to patients with preserved liver function (i.e. no cirrhosis or cirrhosis Child-Pugh class A) (**Suppl. Fig. 3**).

Regarding individual metabolites from the top altered metabolite classes, aspartic acid, glutamic acid, leucine, and serine showed most significantly altered abundance between the four groups with respect to amino acids (all $p < 2.2*10^{-16}$). In addition, CE:14.1, CE:20.0, CE:20.1, CE:22.5, and CE:22.6 (all $p < 2.2*10^{-16}$), xanthine and hypoxanthine (both $p < 2.2*10^{-16}$), glycocholic acid,

glycochenodeoxycholic acid, taurocholic acid, and taurochenodeoxycholic acid (all $p < 1*10^{-9}$), as well as choline ($p < 2.2*10^{-16}$) were among the most significantly altered metabolites of choline esters, nucleobases-related metabolites, bile acids, and vitamins and cofactors, respectively (**Suppl. Fig. 5**).

To obtain a more detailed understanding of which individual metabolites are mostly affected during each step of hepatocarcinogenesis, we computed the differential abundance of all metabolites between the four groups CLD, cirrhosis, initial HCC, and progressed HCC. We found that 16 metabolites were significantly altered in CLD compared to cirrhosis, while eight metabolites were significantly altered in cirrhosis compared to initial HCC, and three metabolites were significantly altered in progressed HCC compared to initial HCC (all with at least 40% differential abundance and FDR < 0.05) (**Fig. 4A, Suppl. Fig. 6A**). When comparing cirrhosis to any HCC (**Fig. 4B, Suppl. Fig. 6B**), and cirrhosis to progressed HCC (**Suppl. Fig. 6C**), 27 and 41 metabolites were significantly altered, respectively.

Our functional lipid network LINEX^2(23) visualizes metabolomic reactions between observed serum lipids of patients with HCC (n=226) and cirrhosis (n=149) (**Suppl. Fig. 11**). The network highlights the already mentioned alterations of CE, TG, and PC lipid classes and also shows that strongly connected and poly-unsaturated TGs (e.g. TG(52:6) FDR=0.0008, TG(55:6) FDR=$4.06*10^{-6}$) are highly increased in HCC (also see supplementary results). Long chain CE also show a strong increase (e.g. CE(22:6) FDR=$5.8*10^{-16}$). For membrane lipids, the strongest increase can be observed for saturated LPCs (e.g. LPC(18:0) FDR=$2.2*10^{-12}$, LPC(16:0) FDR=$1.0*10^{-10}$) and poly-unsaturated PCs (e.g. PC(38:6) FDR=$5.7*10^{-9}$, PC(40:6) FDR=$1.1*10^{-9}$). This indicates an incorporation of poly-unsaturated fatty acids, such as arachidonic acid, in the lipidome.

SUPPLEMENTARY METHODS

Metabolomic profiling from serum specimens and pathway analysis

Serum samples were processed with the MxP® Quant 500 Kit (BIOCRATES Life Sciences AG, Innsbruck, Austria) according to the manufacturer's instructions. Briefly, 10 µL of serum, calibration standards and control samples were transferred onto a filter containing internal standards for standard calibration. Filters were dried under a stream of nitrogen using a pressure manifold (Waters, Eschborn, Germany). Samples were incubated with derivatization reagent phenyl isocyanate for 60 min. After drying under nitrogen, analytes were extracted with 5 mmol/L ammonium acetate in methanol and the eluate was further diluted for the UPLC-MS/MS analysis. The targeted analysis covered 623 metabolites (**Suppl. Table 1**) detected by tandem mass spectrometry (MS/MS) after ultra-high pressure liquid chromatographic (UPLC) separation and flow injection analysis (FIA). Each measurement required two UPLC runs and three FIA runs to cover all metabolites. All analyses were performed on an ACQUITY UPLC I-Class system (Waters) coupled to a Xevo TQ-S mass spectrometer (Waters). Reversed phase chromatographic separation was accomplished using a C18 LC-column (BIOCRATES) with 0.2 % formic acid in water 0.2% formic acid in acetonitrile as an eluent system. FIA solvent was methanol with a modifier which was provided by BIOCRATES. Data analysis of the UPLC-MS/MS results was based on a seven-point curve or one-point calibration and internal standard normalization.

To address possible concerns regarding intrapersonal variability of our assay, we have conducted biological replicates of six patients across four different metabolomic profiling experiments and found that samples from the same patients clustered together (**Suppl. Fig. 13**). This confirms little intrapersonal variability and thus strong reliability of our findings.

For metabolome pathway analysis, the MetaboAnalyst 5.0 tool(25,26) was used.

The semi-quantification of the metabolic profiles in Spanish serum samples was performed, as previously described(43). In brief, UPLC-single quadrupole–MS amino acid analysis system was combined with two separate ultra-high-performance liquid chromatography (UHPLC) time-of-flight–MS based platforms analyzing methanol and chloroform/methanol serum extracts. Data was pre-processed using the TargetLynx application manager for MassLynx 4.1 software (Waters Corp.,

Milford, USA). Metabolites included in the study were identified before the analysis and set an in-house database. Thus, a set of predefined retention time, mass-to-charge ratio pairs, Rt-m/z, corresponding to metabolites included in the analysis are fed into the program. Associated extracted ion chromatograms (mass tolerance window = 0.05 Da) are then peak-detected and noise-reduced in both the LC and MS domains such that only true metabolite related features are processed by the software. A list of chromatographic peak areas is then generated for each sample injection. A total of 424 metabolites in serum samples were identified.

Intra- and inter-batch normalization was performed by inclusion of multiple internal standards and pool calibration response correction using appropriate Quality Controls (QC), following a previously described procedure by Martinez-Arranz et al.(43). This approach solves the instrumental drift in MS-driven metabolomics analysis and potential differences between batches in large cohorts of samples.

Bulk mRNA sequencing from tissue specimen

For the Hamburg cohort, fresh tumoral and adjacent non-tumoral tissue specimens were collected during liver resection surgery for HCC and immediately frozen at -80°C until further analysis. Total RNA was extracted from tissue using the Rneasy® Plus Mini Kit (Qiagen, Hilden, Germany) according to the manufacturer's instructions. Libraries for RNA sequencing were prepared as follows: mRNA was purified from total RNA using poly-T oligo-attached magnetic beads. Further, mRNA was fragmented, converted to cDNA by using random hexamer primers followed by the second strand cDNA synthesis. The library was ready after end repair, A-tailing, adapter ligation, size selection, amplification, and purification. Quality of the library was assessed by Qubit and real-time PCR for quantification and bioanalyzer for size distribution detection. Sequencing was performed using Illumina Novaseq 6000.

Proteome profiling from tissue specimen

For the Hamburg cohort, formalin-fixed paraffin-embedded (FFPE) tissue specimens were deparaffinized for 10 minutes with 100% N-Heptane and reconstituted in 70% Ethanol. Reverse

Formalin Fixation was performed for 1 hour in 0.1M triethylammonium bicarbonate (TEAB) buffer with 1% Sodium Deoxycholate (SDC) at 99°C. The protein concentration was determined by using the Pierce BCA Protein Assay Kit (Thermo Fisher Scientific, Waltham, USA) according to the manufacturer's instructions. 20µg protein per sample was used for tryptic digestion. Tryptic digestion was performed using the Single-pot, solid-phase-enhanced sample preparation (SP3) protocol, as described by Hughes et al.(19). Eluted peptides were dried in a Savant SpeedVac Vacumconcentrator (Thermo Fisher Scientific, Waltham, USA) and stored at -20°C until further use. Directly prior to measurement dried peptides were resolved in 0.1% FA to a final concentration of 1mg/ml. In total 1µg was subjected to mass spectrometric analysis.

Liquid chromatography–tandem mass spectrometer (LC–MS/MS) measurements were performed on a quadrupole-ion-trap-orbitrap MS (Orbitrap Fusion, Thermo Fisher Scientific, Waltham, MA, USA) coupled to a nano-UPLC (Dionex Ultimate 3000 UPLC system, Thermo Fisher Scientific, Waltham, MA, USA). Tryptic peptides were injected to the LC system via an autosampler, purified and desalted by using a reversed phase trapping column (Acclaim PepMap 100 C18 trap; 100 µm × 2 cm, 100 A pore size, 5 µm particle size; Thermo Fisher Scientific, Waltham, MA, USA), and thereafter separated with a reversed phase column (Acclaim PepMap 100 C18; 75 µm × 25 cm, 100 A pore size, 2 µm particle size, Thermo Fisher Scientific, Waltham, MA, USA). Trapping was performed for 5 min at a flow rate of 5 µL/min with 98% solvent A (0.1% FA) and 2% solvent B (0.1% FA in ACN). Separation and elution of peptides were achieved by a linear gradient from 2 to 30% solvent B in 65 min at a flow rate of 0.3 µL/min. Eluting peptides were ionized by using a nano-electrospray ionization source (nano-ESI) with a spray voltage of 1800 V, transferred into the MS and analyzed in data dependent acquisition (DDA) mode. For each MS1 scan, ions were accumulated for a maximum of 120 ms or until a charge density of 2×10^5 ions (AGC target) was reached. Fourier-transformation-based mass analysis of the data from the orbitrap mass analyzer was performed by covering a mass range of 400–1300 m/z with a resolution of 120,000 at m/z = 200. Peptides with charge states between 2+–5+ above an intensity threshold of 1000 were isolated within a 1.6 m/z isolation window in top-speed mode for 3 s from each precursor scan and fragmented with a normalized collision energy of 30%, using higher energy collisional dissociation (HCD). MS2 scanning was performed, using an ion trap mass

analyzer, covering a mass range of 380–1500 m/z with an orbitrap resolution of 15,000 at m/z = 200 and accumulated for 60 ms or to an AGC target of 1×10^5. Already fragmented peptides were excluded for 30 s.

LC-MS/MS raw spectra were searched with the Sequest algorithm integrated in the Proteome Discoverer software (v 2.41.15, Thermo Fisher Scientific) against a reviewed human Swissprot database, obtained in April 2020, containing 20365 entries. Carbamidomethylation was set as fixed modification for cysteine residues and the oxidation of methionine, and pyro-glutamate formation at glutamine residues at the peptide N-terminus, as well as acetylation of the protein N-terminus were allowed as variable modifications. A maximum number of 2 missing tryptic cleavages was set. Peptides between 6 and 144 amino acids were considered. A strict cutoff (false discovery rate (FDR) <0.01) was set for peptide and protein identification. Protein quantification was carried out, using the Minora Algorithm, implemented in Proteome Discoverer(20). Protein abundance values were log2 transformed and median normalized across columns to compensate for injection amount differences.

in vitro knockdown experiments

Cells isolation, maintenance and transfection: Huh-7 cell lines were provided by Wege lab. Cells were authenticated periodically by short tandem repeat DNA fingerprinting and checked monthly for mycoplasma infection by PCR. Huh7 and Huh7shRNA clones were cultured in Dulbecco’s modified Eagle’s medium (DMEM) 4.5 g/L Glucose (Capricorn, GmbH) containing 10% FBS (Capricorn, GmbH) and 1% penicillin–streptomycin. Cells were maintained at 37˚C under 5% CO_2.

One set of four different PYCR2-specific shRNA lentiviral particles packaged from pGFP-C-shLenti vector was obtained from Origene (Catalog No. TL310026V; OriGene Technologies, Inc.). Out of them, shRNA TL310026VB (i.e. clone B) and TL3310026VC (i.e. clone C) were the most efficient and hence selected for all described experiments. We used TR30021V Lenti shRNA scramble particles from the same company as a negative control in all transfection experiments.

For generation of stable cell lines, $25x10^3$ cells/cm^2 were seeded in a 24-well plate. After 24 h they were incubated with specific and control-shRNA particles at 10 MOI/cell and 8 mg/ml Polybrene Reagent (Sigma Aldrich, Merck, KGaA). After 16 h of incubation, the transfection medium was

replaced by standard growth medium. After 24 h, cells were replenished with fresh standard growth medium containing Puromycin [0.75µg/ml] (InvivoGen, Inc.) every 2 days until the majority of the uninfected control cells were eliminated (in ~ 7–10 days). After the transfection, cells were collected and analyzed as described in the following section.

Western blotting: 30 µg of proteins extracted with RIPA Buffer and Protease and Phosphatase inhibitors (Life Technologies, GmbH) were separated on SDS-PAGE and transferred to PVDF membrane. The membranes were incubated at 4˚C for 16 h with a rabbit polyclonal anti-PYCR2 (1:4000, no. 17146-1-AP; Proteintech, Inc.) and a monoclonal mouse anti-actin (clone AC-15; 1:2000, no. sc-69879; Santa Cruz Biotechnology, Inc.). Membranes were washed three times with TBS 0.05% Tween and incubated for 1 h with HRP-conjugated anti-rabbit IgG (1:2000;Cell Signaling Technology. Inc.) or anti- mouse IgG (1:2000; Cell Signaling Technology. Inc.). Immunocomplexes were detected by a chemiluminescence detection kit (Clarity Western ECL Substrate Kit; Bio-Rad Laboratories, Inc.) with iBright 750 Imaging System (Thermo Fisher Scientific, Inc.). The densitometric analysis was performed with the ImageJ program (National Institutes of Health; https://imagej.net/). Uncropped Western Blot images are provided in **Suppl. Fig 14**.

RNA extraction and reverse transcription-quantitative polymerase chain reaction (RT-qPCR): RT-qPCR experiments were performed following MIQE guidelines. RNA was purified by using TRIzol™ solution (Thermo Fisher Scientific, Inc.). 500 ng of total RNA was used to synthesize the first strand of cDNA with the High-Capacity cDNA Reverse Transcription Kit (Thermo Fisher Scientific, Inc.). 5 ng of cDNA was used as a template for the real-time amplification. RT-qPCR was performed with a TaqMan™ Gene Expression assay for each gene of interest and TaqMan™ Fast Advanced Master Mix (Thermo Fisher Scientific, Inc.) using the QuantStudio™ 5 Real Time PCR System (Thermo Fisher Scientific, Inc.). Glyceraldehyde-3-phosphate dehydrogenase (GAPDH) mRNA was used as an endogenous reference for the relative quantification. The following TaqMan™ Gene Expression assays were used: Human PYCR2 Hs01016460_gH and Human GAPDH Hs99999905_m1.

Immunocytochemistry: $20x10^3$ cells/cm^2 were seeded in each well of an 8-well chamber (Sarstedt, GmbH). After 24h, cells were fixed with 10% formalin and immunocytochemistry was performed. Briefly, endogenous peroxidase activity was blocked with 0,3% H_2O_2 in methanol for 20

min. Antigen retrieval was performed by using a microwave-oven in 1.0 mM EDTA buffer (pH 8.0). Slides were then incubated for one hour with a rabbit polyclonal anti-PYCR2 (1:3000, no. 17146-1-AP; Proteintech, Inc.) diluted in PBS 1% Bovine Serum Albumin (BSA). Signal was revealed using the Dako REAL EnVision Detection System HRP Rabbit/Mouse (AgilentTechnologies. Inc.), followed by Diaminobenzydine (DAB) as chromogen and Hematoxylin as counterstain. Images were acquired at 10X magnification with a Kern OBE 124T241 microscope equipped with a Tablet ODC 241 camera and S-EYE 1.10.7 imaging software (Kern & Sohn, GmbH).

Cell viability/proliferation: 20x 10^3 cells/cm^2 were seeded in 48-well plates in triplicates. After 24h, 48h and 72h adherent cells were detached with trypsin 0.1%/EDTA, stained with trypan blue to check for vitality and counted excluding dead cells.

Colony formation assay: A total of 2×10^3 cells were seeded in 6-well plates and grown at 37°C, 5% CO_2 for two weeks, while the medium was replaced every three days. The colonies were fixed with 10% formalin and stained for 15 minutes with 0.1% crystal violet. The number of colonies were counted with a light microscope.

Cell migration assay: For the migration assay, 8-mm pore Transwell inserts (Corning, Inc.) were seeded with 50x10^3 cells/well in DMEM 4.5 g/L Glucose without FBS. DMEM 10% FBS was used in the lower chamber as a stimulus. After 12 hours of incubation, cells were fixed with 10% formalin, permeabilized with methanol and stained with Giemsa dye for 15 minutes. Non-invasive cells on the upper surface of the filter were removed by wiping with a cotton swab. The cells that migrated to the lower surface of the membrane were manually counted in five fields under a light microscope. Data were expressed as the average number of cells migrating through the filters.

Supplementary references

19. Hughes CS, Moggridge S, Müller T, et al. Single-pot, solid-phase-enhanced sample preparation for proteomics experiments. Nat. Protoc. 2019;14:68–85.

25. Pang Z, Chong J, Zhou G, et al. MetaboAnalyst 5.0: narrowing the gap between raw spectra and functional insights. Nucleic Acids Res. 2021;49:W388–W396.

26. Pang Z, Zhou G, Ewald J, et al. Using MetaboAnalyst 5.0 for LC–HRMS spectra processing, multi-omics integration and covariate adjustment of global metabolomics data. Nat. Protoc. 2022;17:1735–1761.

43. Martínez-Arranz I, Mayo R, Pérez-Cormenzana M, et al. Enhancing metabolomics research through data mining. J. Proteomics. 2015;127:275–288.

(numbering follows that of the main manuscript)

SUPPLEMENTARY TABLES

Suppl Table 2 Clinical characteristics of "serum metabolome identification cohort" for biomarker analysis: cirrhosis vs. any HCC

Characteristic	N	Overall, N = 375[1]	Cirrhosis, N = 149[1]	HCC, N = 226[1]	p-value[2]
**Sex**	334				<0.001
**f**		97 (29%)	61 (41%)	36 (19%)	
**m**		237 (71%)	88 (59%)	149 (81%)	
**Age**	334				0.007
**<60**		111 (33%)	61 (41%)	50 (27%)	
**>=60**		223 (67%)	88 (59%)	135 (73%)	
**Diabetes**	244	93 (38%)	17 (38%)	76 (38%)	>0.9
**BMI (kg/m²)**	155				0.8
**<25**		61 (39%)	22 (37%)	39 (41%)	
**25-30**		43 (28%)	18 (31%)	25 (26%)	
**>30**		51 (33%)	19 (32%)	32 (33%)	
**Etiology**	375				0.002
**Alcohol**		80 (21%)	45 (30%)	35 (15%)	
**NASH**		52 (14%)	17 (11%)	35 (15%)	
**Viral**		154 (41%)	49 (33%)	105 (46%)	
**Other**		89 (24%)	38 (26%)	51 (23%)	
**Cirrhosis**	371	290 (78%)	149 (100%)	141 (64%)	<0.001
**Child stage**	223				0.2
**A**		159 (71%)	90 (67%)	69 (78%)	
**B**		62 (28%)	42 (31%)	20 (22%)	
**C**		2 (0.9%)	2 (1.5%)	0 (0%)	
**ALBI score**	282	-2.34 (-2.73, -1.74)	-2.26 (-2.76, -1.54)	-2.40 (-2.70, -1.89)	0.091
**BCLC**	226				>0.9
**0**		27 (12%)	0 (NA%)	27 (12%)	
**A**		154 (68%)	0 (NA%)	154 (68%)	
**B**		19 (8.4%)	0 (NA%)	19 (8.4%)	
**C**		25 (11%)	0 (NA%)	25 (11%)	
**D**		1 (0.4%)	0 (NA%)	1 (0.4%)	
**AFP (ng/mL)**	254	6 (4, 33)	4 (2, 5)	12 (4, 104)	<0.001

1 Statistics presented: n (%); median (IQR); 2 Statistical tests performed: chi-square test of independence; Kruskal-Wallis test; Fisher's exact test; AFP, alpha fetoprotein, BCLC, Barcelona Clinic for Liver Cancer classification, NASH, non-alcoholic steatohepatitis

Suppl Table 3 Clinical characteristics of "serum metabolome identification cohort" for biomarker analysis: cirrhosis vs early-stage BCLC0/A-HCC

Characteristic	N	Overall, N = 330[1]	Cirrhosis, N = 149[1]	HCC, N = 181[1]	p-value[2]
**Sex**	316				<0.001
**f**		93 (29%)	61 (41%)	32 (19%)	
**m**		223 (71%)	88 (59%)	135 (81%)	
**Age**	316				0.009
**<60**		106 (34%)	61 (41%)	45 (27%)	
**>=60**		210 (66%)	88 (59%)	122 (73%)	
**Diabetes**	204	74 (36%)	17 (38%)	57 (36%)	0.8
**BMI (kg/m²)**	114				0.6
**<25**		38 (33%)	22 (37%)	16 (29%)	
**25-30**		38 (33%)	18 (31%)	20 (36%)	
**>30**		38 (33%)	19 (32%)	19 (35%)	
**Etiology**	330				<0.001
**Alcohol**		69 (21%)	45 (30%)	24 (13%)	
**NASH**		48 (15%)	17 (11%)	31 (17%)	
**Viral**		132 (40%)	49 (33%)	83 (46%)	
**Other**		81 (25%)	38 (26%)	43 (24%)	
**Cirrhosis**	326	261 (80%)	149 (100%)	112 (63%)	<0.001
**Child stage**	218				0.3
**A**		154 (71%)	90 (67%)	64 (76%)	
**B**		62 (28%)	42 (31%)	20 (24%)	
**C**		2 (0.9%)	2 (1.5%)	0 (0%)	
**ALBI score**	271	-2.34 (-2.73, -1.72)	-2.26 (-2.76, -1.54)	-2.40 (-2.70, -1.93)	0.089
**BCLC**	181				
**0**		27 (15%)	0 (NA%)	27 (15%)	
**A**		154 (85%)	0 (NA%)	154 (85%)	
**Treatment-naive**	167	160 (96%)	0 (NA%)	160 (96%)	
**AFP (ng/mL)**	236	6 (3, 29)	4 (2, 5)	10 (4, 102)	<0.001

1 Statistics presented: n (%); median (IQR); 2 Statistical tests performed: chi-square test of independence; Kruskal-Wallis test; Fisher's exact test; AFP, alpha fetoprotein, BCLC, Barcelona Clinic for Liver Cancer classification, NASH, non-alcoholic steatohepatitis

Suppl Table 4 Performance of individual metabolite candidates to discriminate early-stage HCC (BCLC 0/A, n=181) and cirrhosis (n=149)

		Best performance		Specificity fixed at 85%		Specificity fixed at 90%	
**Metabolite**	**AUC**	**Sens.**	**Spec.**	**Sens.**	**Spec.**	**Sens.**	**Spec.**
**aspartic acid**	90.8%	88.3%	76.4%	77.3%	85%	68.9%	90%
**glutamic acid**	81.3%	71.7%	77.0%	52.2%	85%	43.9%	90%
**hypoxanthine**	80.5%	87.8%	60.1%	54.4%	85%	38.3%	90%
**taurine**	81.7%	74.4%	80.4%	65%	85%	57.2%	90%
**AFP[1]**	77.2%	57.5%	94.7%	61.9%	85%	60.6%	90%

1 AFP values only available for n=236 (BCLC 0/A n=160; cirrhosis n=76).

Suppl Table 5 Performance of 10-metabolite signature +/- AFP to discriminate early-stage HCC (BCLC 0/A) and cirrhotic controls by subgroup (average metrics reported for testing data set after 1,000 internal cross-validations splitting the cohort into (70% training and 30% testing sets)

Subgroup	10-metabolite			10-metabolite + AFP		
	AUC	Sens.	Spec	AUC	Sens.	Spec
**Overall**	92%	83%	82%	94%	86%	84%
**Sex**						
Male	92%	83%	80%	94%	84%	83%
Female	92%	84%	81%	94%	83%	84%
**Etiology**						
Alcohol	99%	97%	92%	99%	95%	90%
Viral	83%	72%	73%	87%	73%	77%
NASH	93%	82%	86%	94%	84%	84%
Other	94%	87%	83%	96%	88%	84%

For subgroup analysis regarding sex and etiology cirrhotic controls and HCC-BCLC 0/A were subsetted according to subgroup (male, female, alcohol, viral, etc). Subgroup analysis for cirrhotic included only cirrhotic controls and cirrhotic HCC, while non-cirrhotic background included non-cirrhotic controls and non-cirrhotic HCC. AUC, Area-under-the-receiver-operating-curve, Sens, sensitivity, Spec., specificity.

Suppl Table 6 Performance of 10-metabolite signature +/- AFP to discriminate early-stage HCC (BCLC 0/A) and controls stratified by presence of cirrhosis and for patients with AFP≤20ng/mL.
(average metrics reported for testing data set after 1,000 internal cross-validations splitting the cohort into (70% training and 30% testing sets)

Subgroup	10-metabolite			10-metabolite + AFP		
	AUC	Sens.	Spec	AUC	Sens.	Spec
**Background**						
cirrhotic	89%	79%	81%	94%	84%	84%
non-cirrhotic	87%	75%	79%	89%	77%	84%
**AFP ≤20ng/mL**	87%	77%	81%	88%	77%	83%

Subgroup analysis for cirrhotic included only cirrhotic controls and cirrhotic HCC, while non-cirrhotic background included non-cirrhotic controls and non-cirrhotic HCC. Subgroup analysis for AFP≤20ng/mL included all patients with AFP ≤20ng/mL irrespective of etiology or presence of cirrhosis. AUC, Area-under-the-receiver-operating-curve, Sens, sensitivity, Spec., specificity.

Suppl Table 7. Clinical characteristics external validation cohort (Spain)

Characteristic	N	Overall, N = 102[1]	control, N = 35[1]	HCC, N = 32[1]	iCCA, N = 35[1]
**Sex**	102				
**f**		37 (36%)	20 (57%)	2 (6.2%)	15 (43%)
**m**		65 (64%)	15 (43%)	30 (94%)	20 (57%)
**Age**	102	63 (54, 69)	56 (47, 64)	58 (54, 67)	70 (65, 75)
**Diabetes**	102	20 (20%)	3 (8.6%)	10 (31%)	7 (20%)
**BMI**	75	26.2 (23.8, 29.1)	25.6 (23.0, 28.0)	26.2 (24.9, 29.1)	27.0 (23.3, 30.4)
**Etiology**	32				
**Alcohol**		12 (38%)	0 (NA%)	12 (38%)	0 (NA%)
**NASH**		1 (3.1%)	0 (NA%)	1 (3.1%)	0 (NA%)
**Viral**		11 (34%)	0 (NA%)	11 (34%)	0 (NA%)
**Other**		8 (25%)	0 (NA%)	8 (25%)	0 (NA%)
**Cirrhosis**	66	35 (53%)	0 (NA%)	28 (88%)	7 (21%)
**Child stage**	31				
**A**		24 (69%)	0 (NA%)	15 (79%)	9 (56%)
**B**		7 (20%)	0 (NA%)	4 (21%)	3 (19%)
**BCLC**	32				
**A**		27 (84%)	0 (NA%)	27 (84%)	0 (NA%)
**B**		2 (6.2%)	0 (NA%)	2 (6.2%)	0 (NA%)
**C**		3 (9.4%)	0 (NA%)	3 (9.4%)	0 (NA%)
**TNM stage**	35				
**I**		15 (43%)	0 (NA%)	0 (NA%)	15 (43%)
**II**		5 (14%)	0 (NA%)	0 (NA%)	5 (14%)
**III**		5 (14%)	0 (NA%)	0 (NA%)	5 (14%)
**IV**		10 (29%)	0 (NA%)	0 (NA%)	10 (29%)
**AFP (ng/mL)**	73	3 (2, 6)	2 (2, 3)	7 (3, 71)	3 (2, 3)

1 Statistics presented: n (%); median (IQR); AFP, alpha fetoprotein, BCLC, Barcelona Clinic for Liver Cancer classification, NASH, non-alcoholic steatohepatitis

Suppl Table 8. Clinical characteristics for German biological validation cohort (N=81)

Characteristic	N	Germany
**Sex**	78	
**f**		21 (27%)
**m**		57 (73)
**Age**	54	67
**Etiology**	61	
**Alcohol**		13 (21%)
**NASH**		7 (11%)
**Viral**		14 (23%)
**Other**		19 (31%)
**None**		8 (13%)
**Cirrhosis**	60	28 (47%)
**BCLC**	59	
**0/A**		47 (80%)
**B**		10 (17%)
**C**		2 (3%)

Statistics presented: n (%); median; BCLC, Barcelona Clinic for Liver Cancer classification, NASH, non-alcoholic steatohepatitis.

SUPPLEMENTARY FIGURES

Suppl. Figure 1

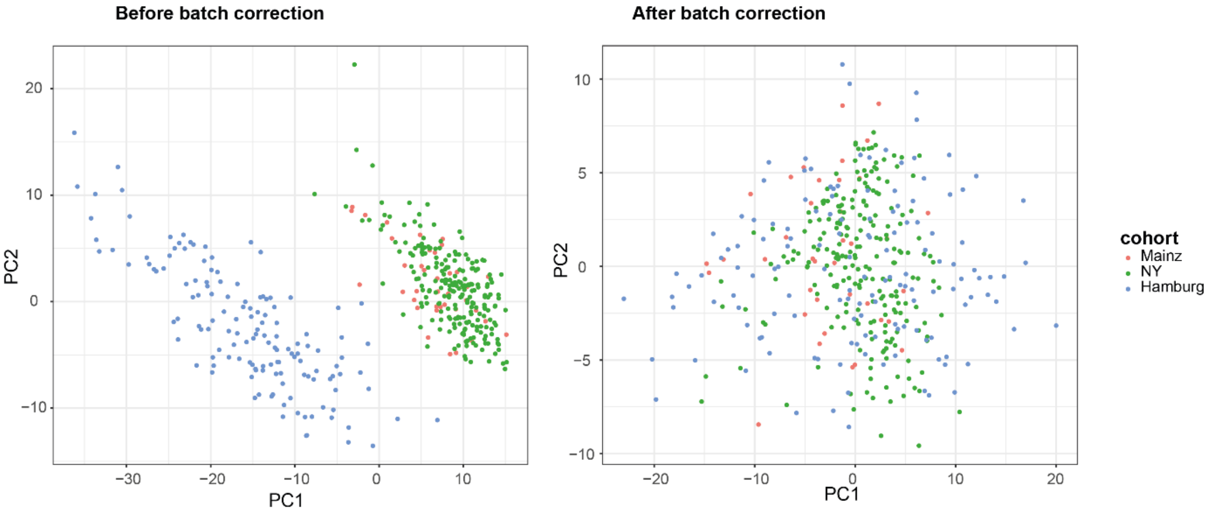

Suppl. Figure 1. Principal component analysis for the serum metabolomics dataset (n=406) for all cohorts before and after batch correction.

Suppl. Figure 2

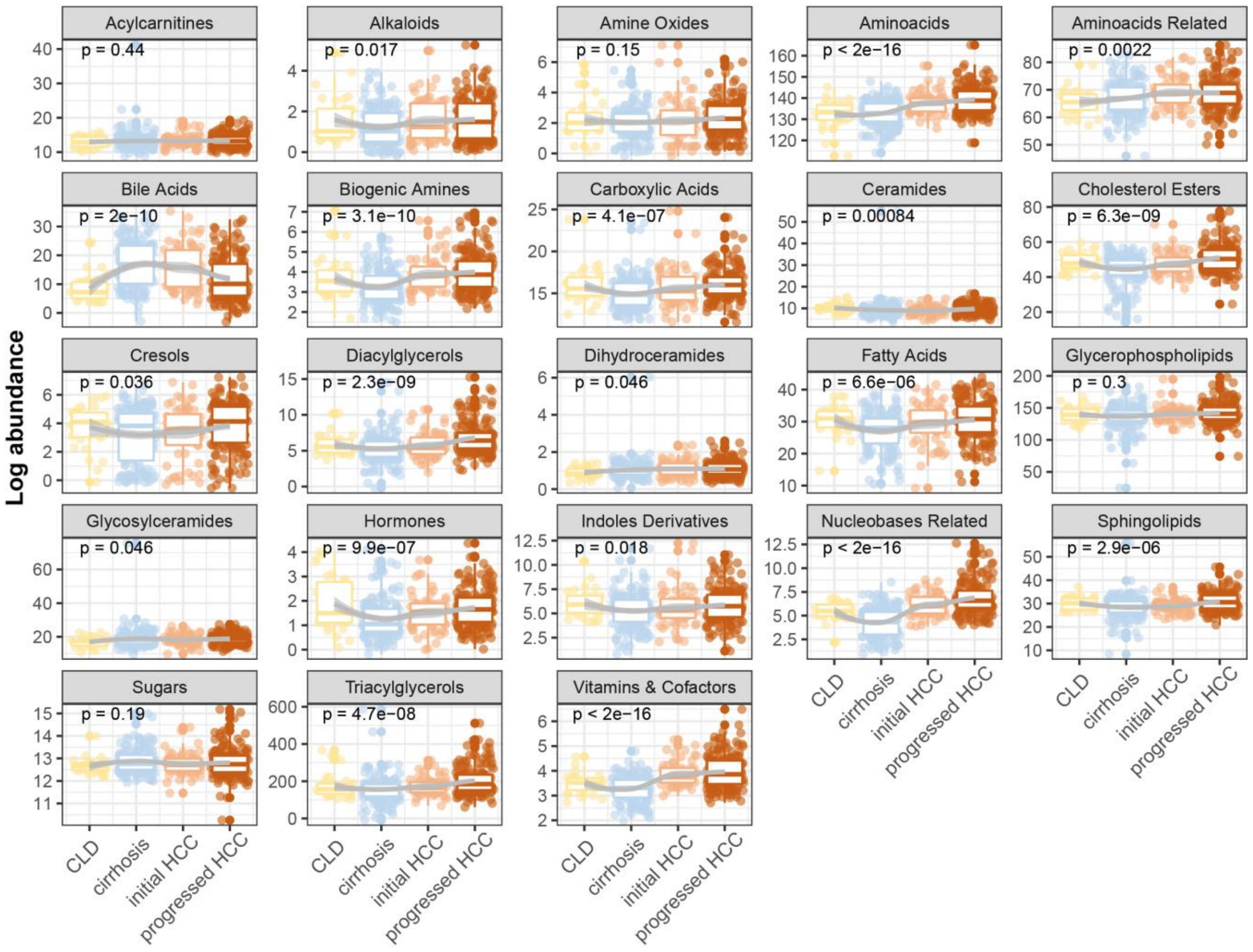

Suppl. Figure 2. Full plot of abundance of altered metabolite classes in sera across patients with chronic liver disease (CLD), cirrhosis, initial HCC, and progressed HCC (ANOVA).

Suppl. Figure 3

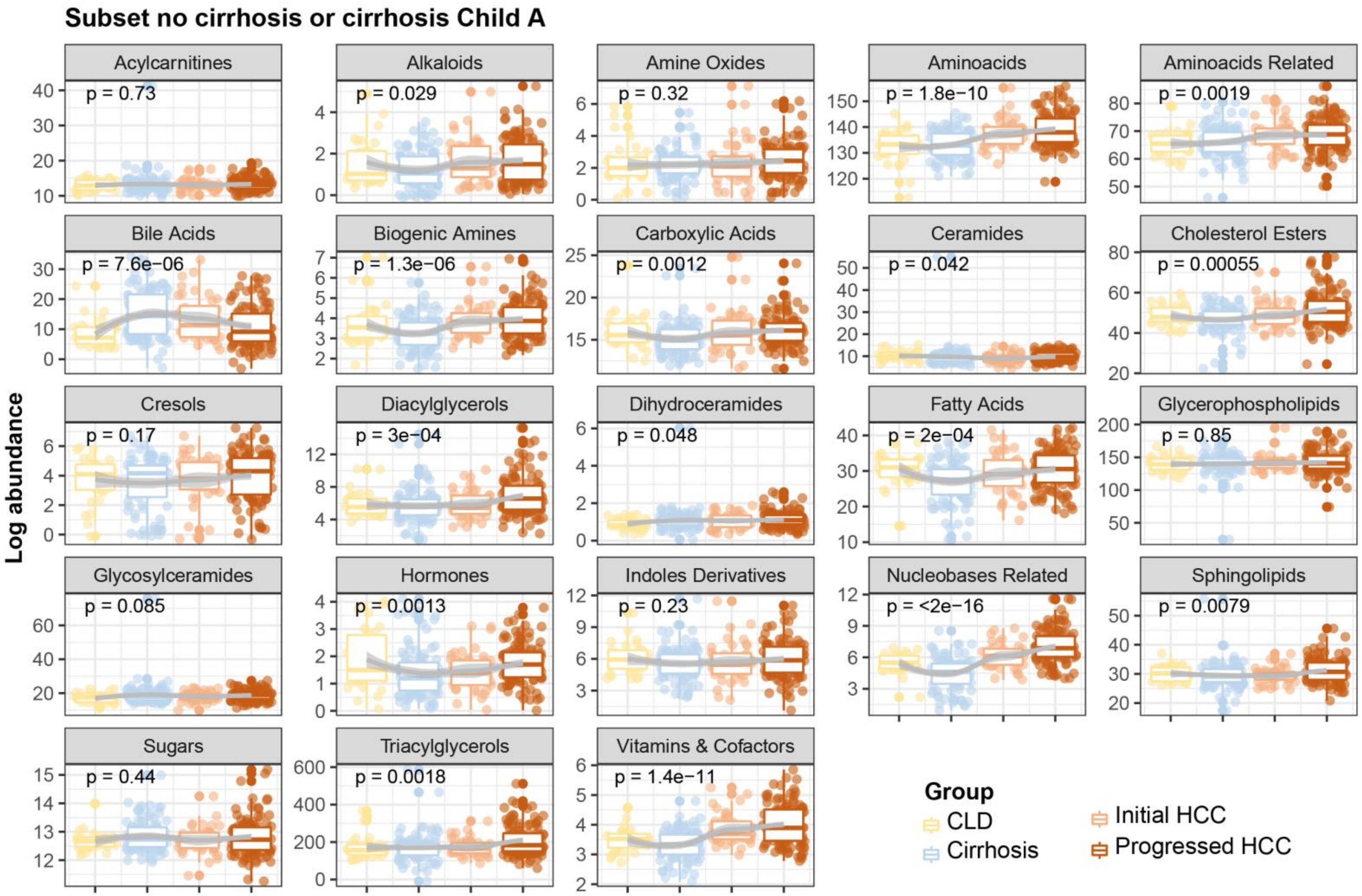

Suppl. Figure 3. Abundance of metabolite classes in sera across patients with chronic liver disease (CLD), cirrhosis, initial HCC, and progressed HCC subsetted to patients without cirrhosis or with cirrhosis Child A.

Suppl. Figure 4

A.

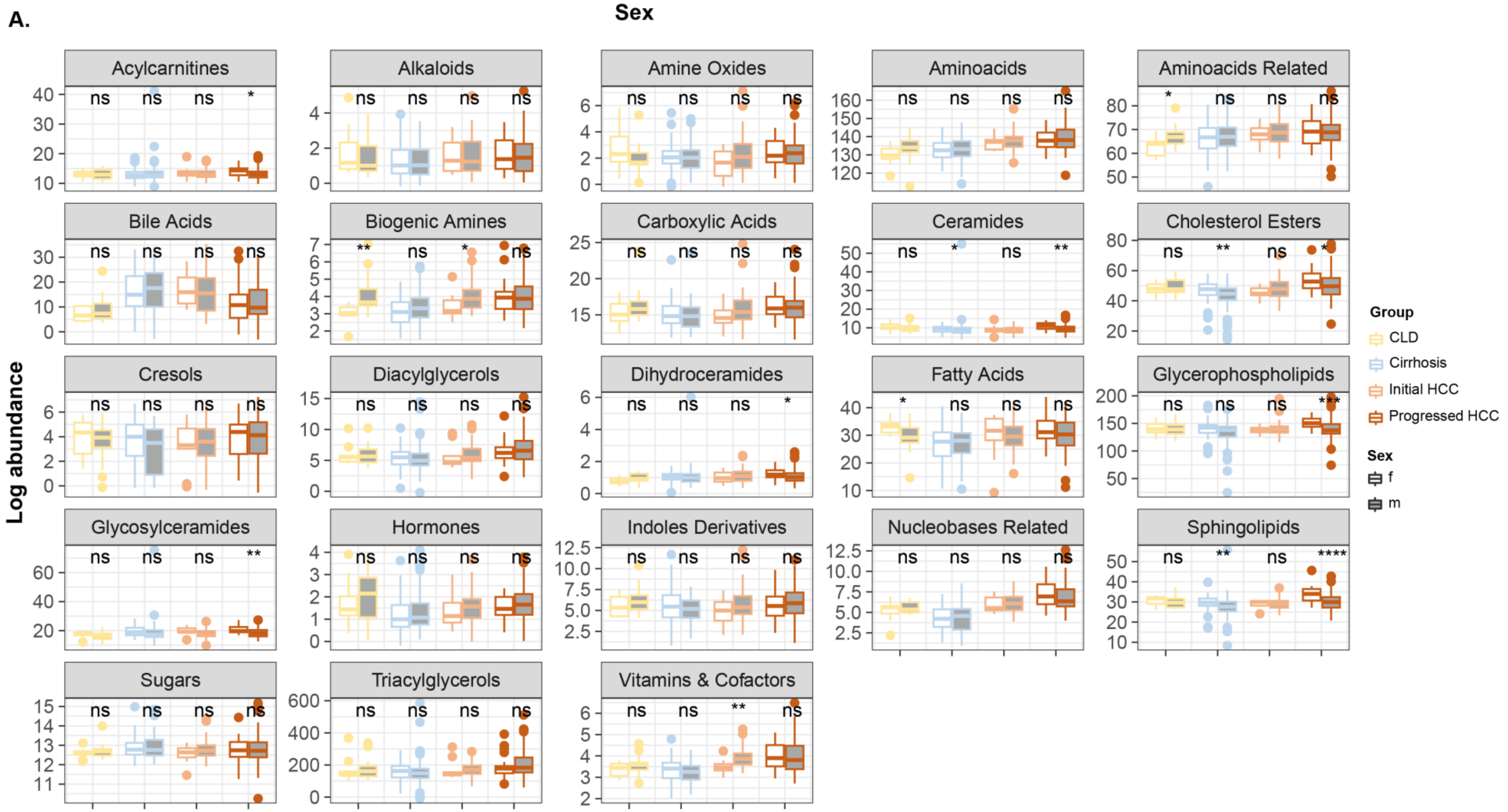

Suppl. Figure 4

B.

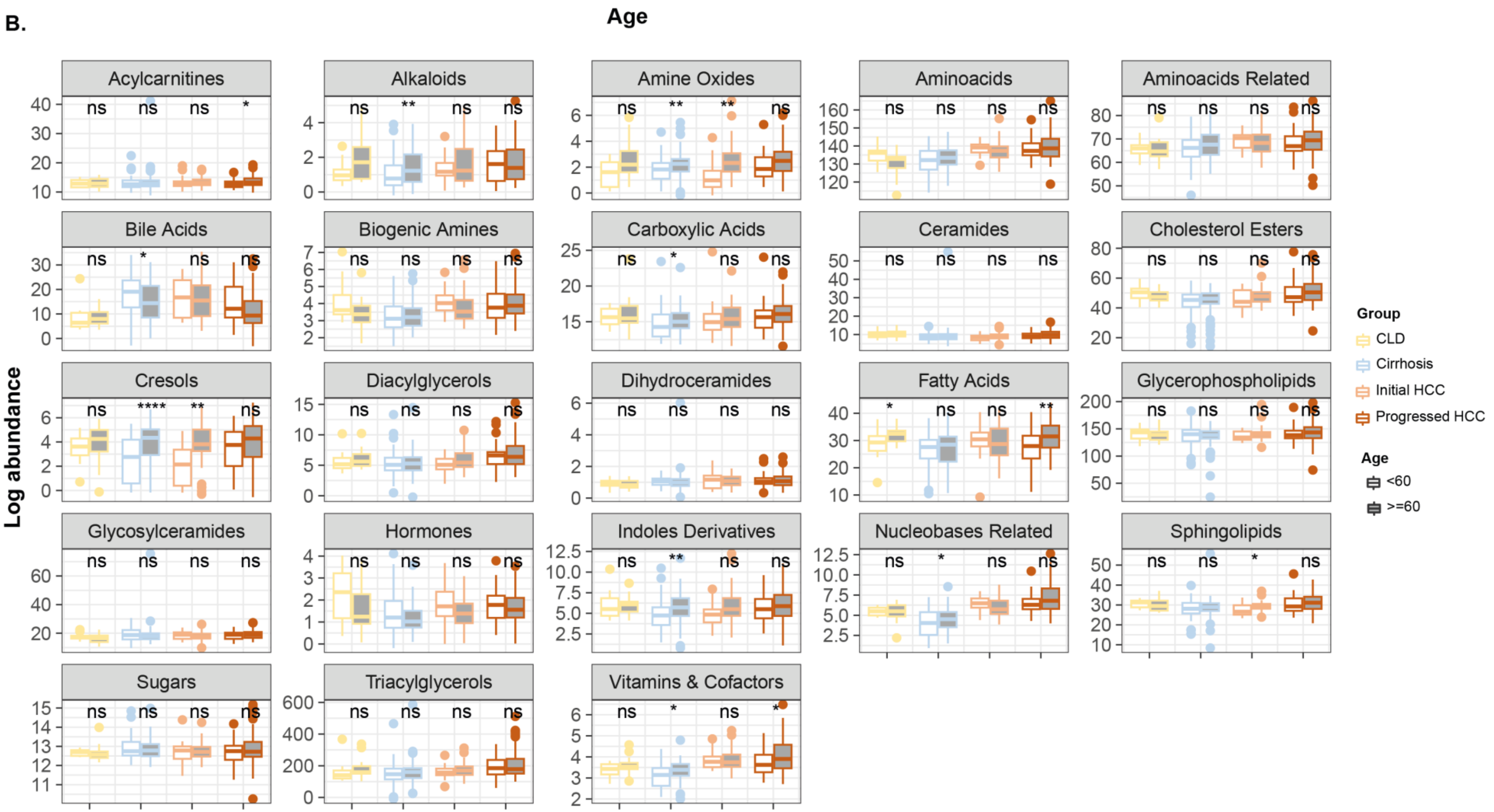

Suppl. Figure 4

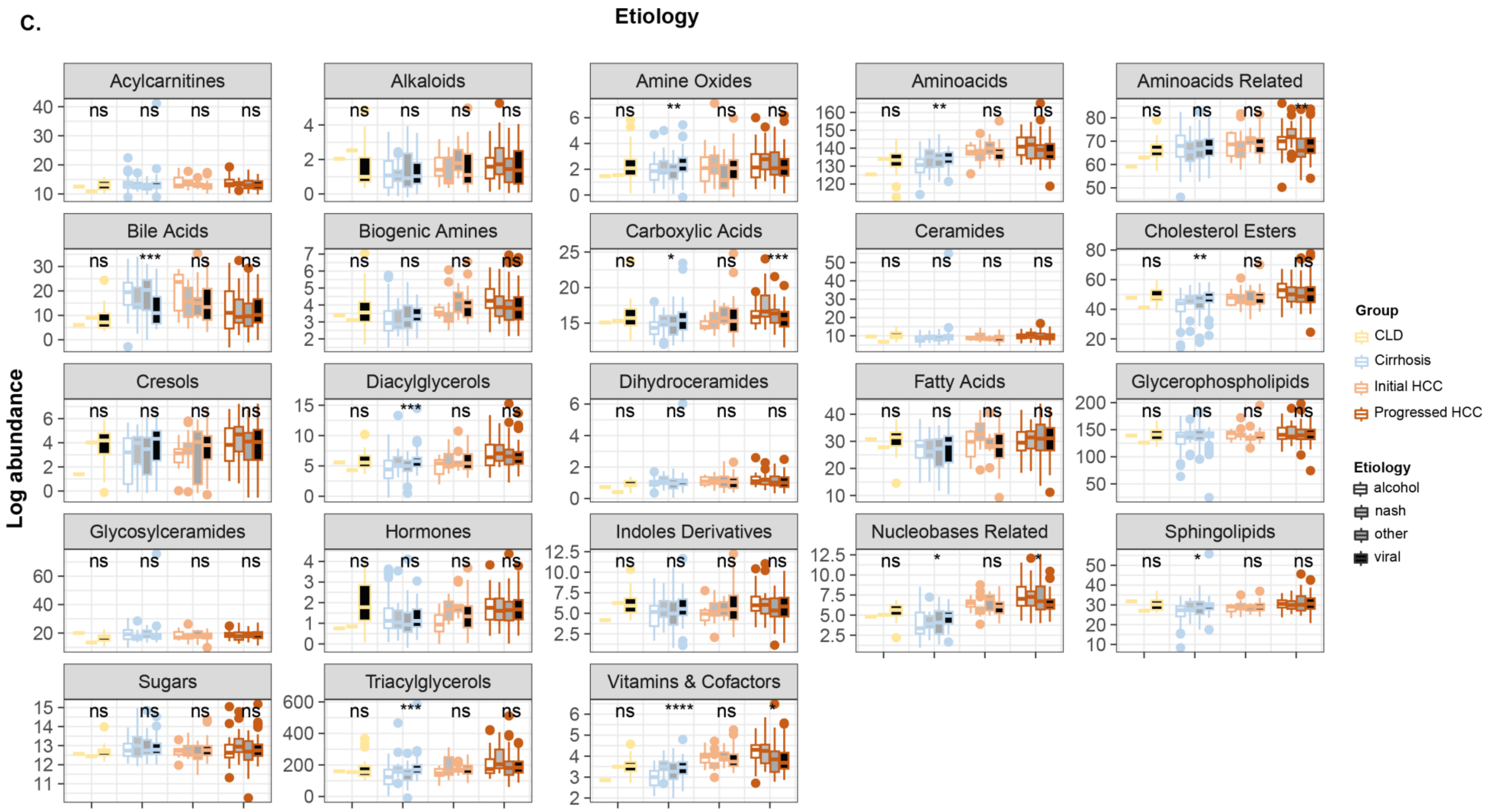

Suppl. Figure 4. Abundance of metabolite classes in sera across patients with chronic liver disease (CLD), cirrhosis, initial HCC, and progressed HCC by sex (A), age dichotomized into <60 and >= 60 years (B), and etiology (C).

Suppl. Figure 5

Aminoacids, Nucleobases Related, Bile Acids, Cholesterol Esters, Vitamins & Cofactors

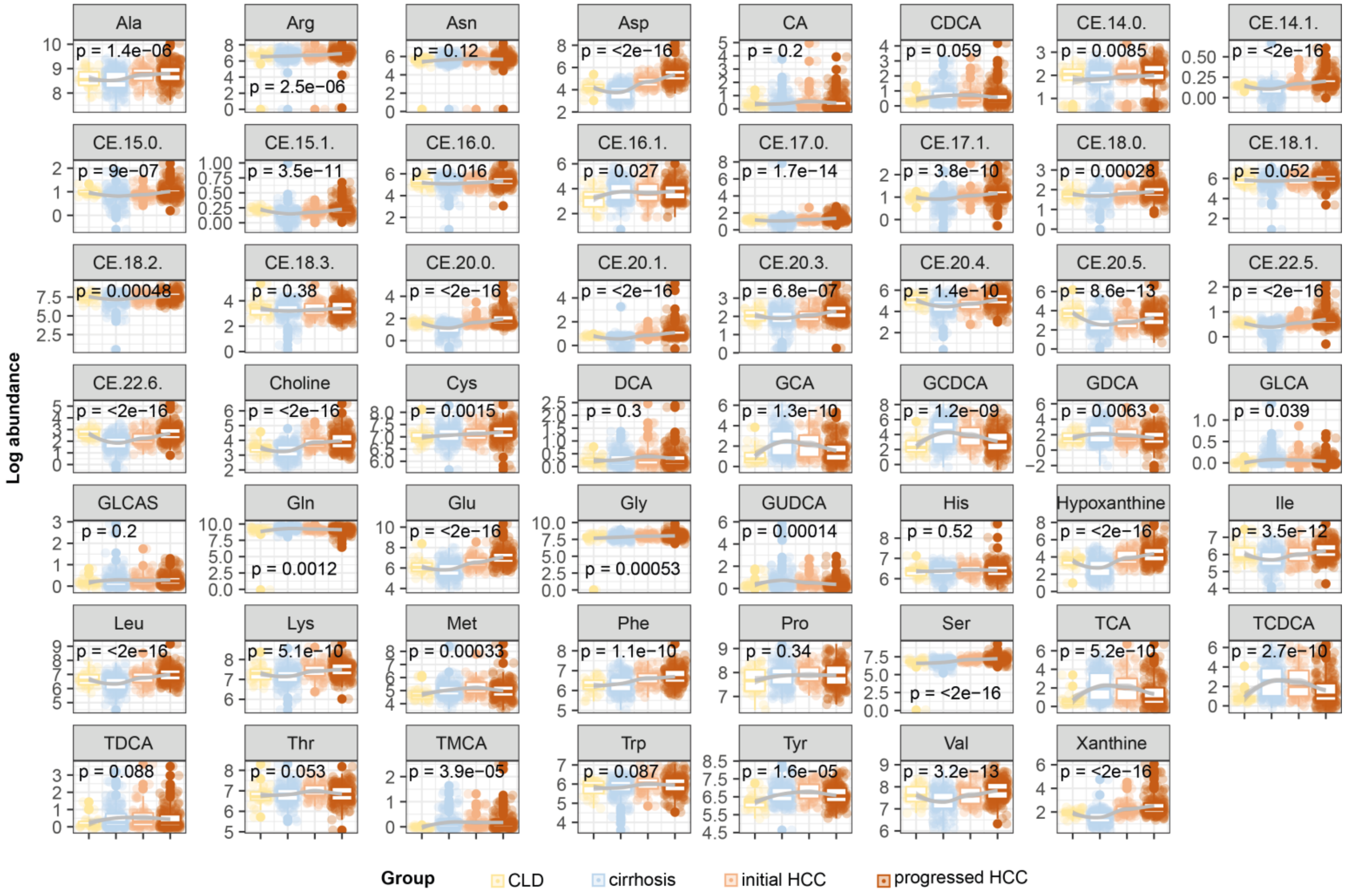

Suppl. Figure 5. Abundance of individual metabolites from selected classes (i.e. amino acids, nucleobases related, bile acids, cholesterol acids, and vitamins & cofactors) in sera across patients with chronic liver disease (CLD), cirrhosis, initial HCC, and progressed HCC.

Suppl. Figure 6

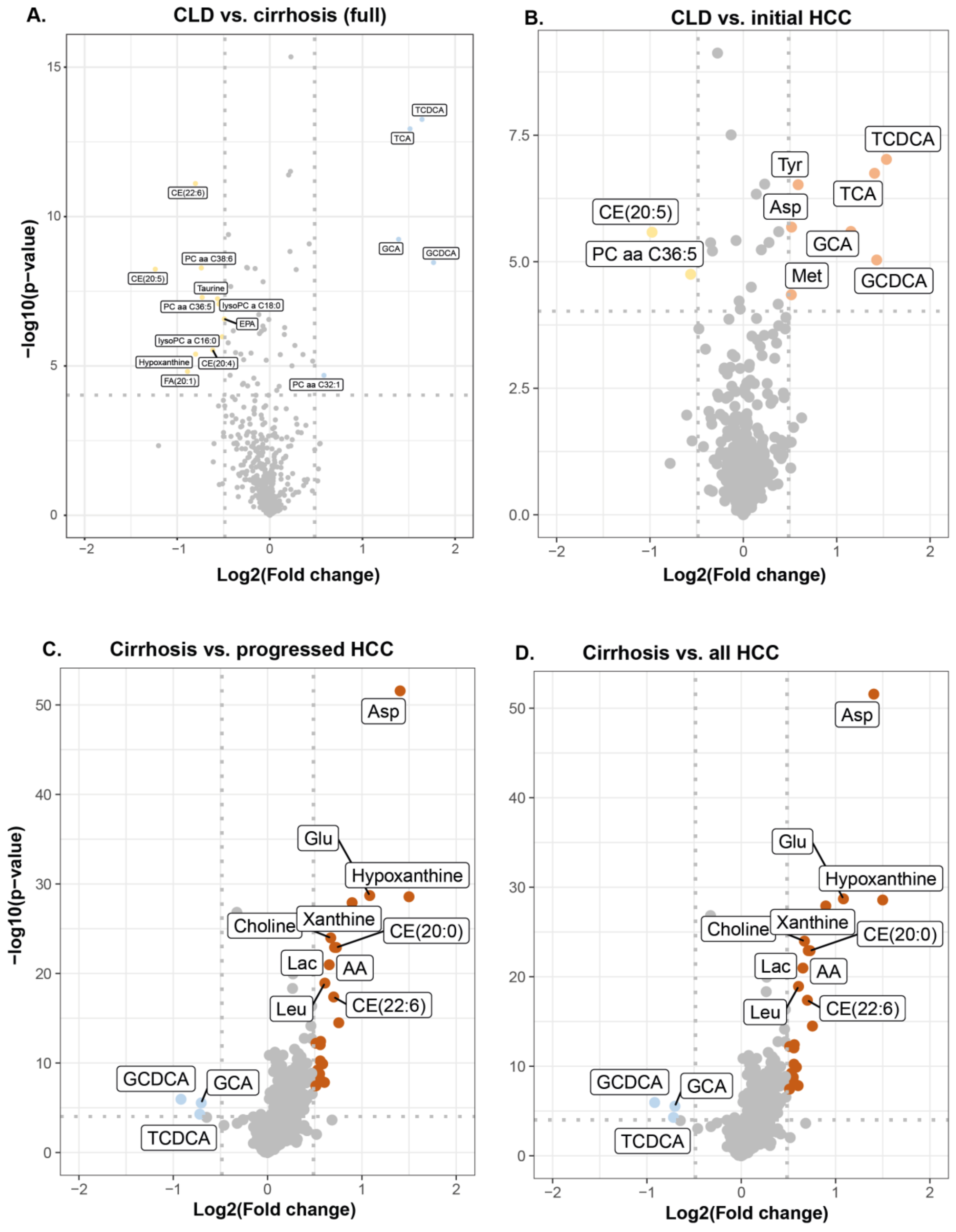

Suppl. Figure 6. Volcano plot displaying differential abundant metabolites between (A) CLD (yellow) and cirrhosis (blue) (with all labels), (B) CLD (yellow) and initial HCC (orange), (C) cirrhosis (blue) and progressed HCC (dark red), and (D) cirrhosis (blue) and all HCC (dark red).

Suppl. Figure 7

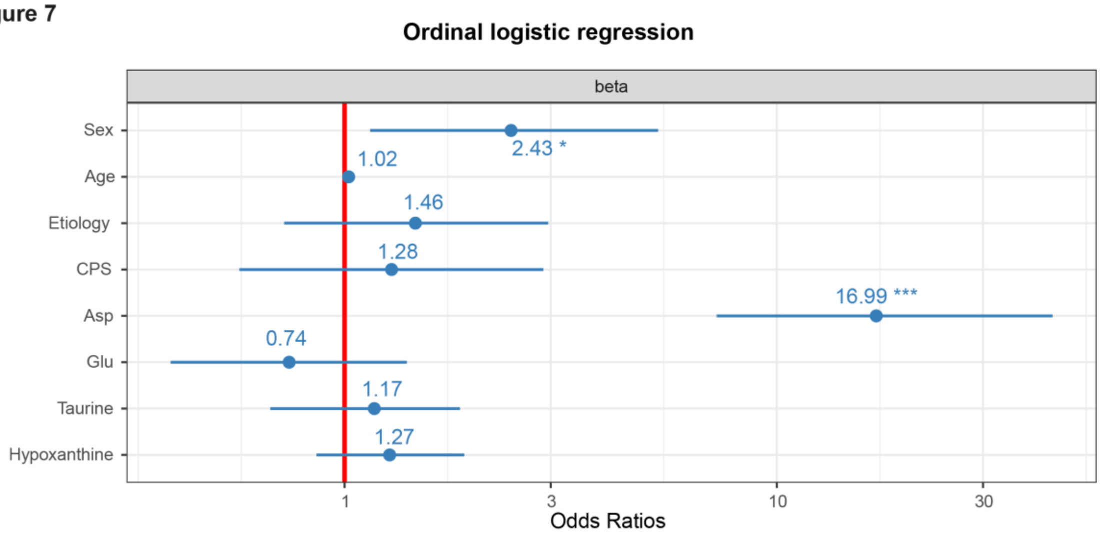

Suppl. Figure 7. Ordinal logistic regression model incl. clinical variables sex, age, etiology, and all four candidate metabolites.

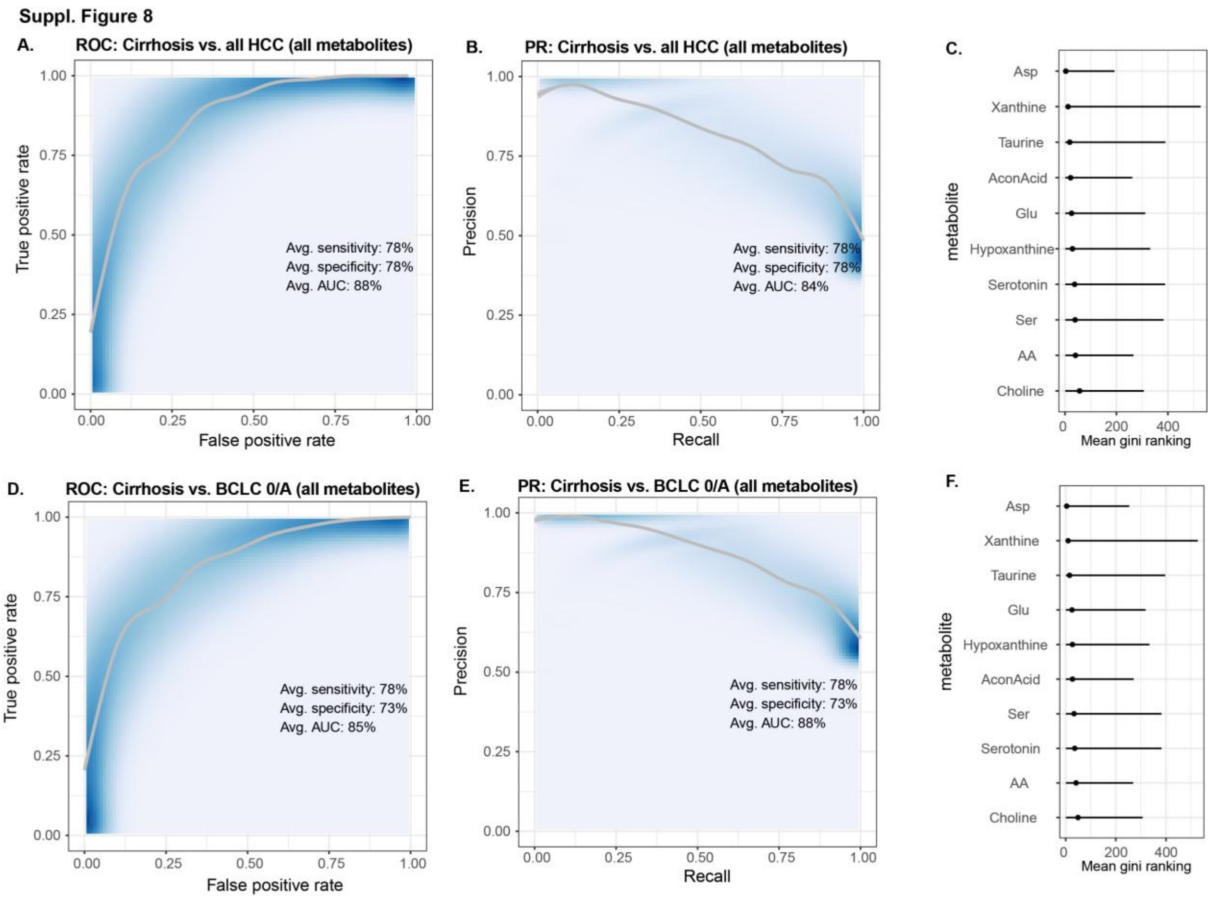

Suppl. Figure 8. Average Area-under-the ROC-curve (panel A+D) and precision recall curve (PR, panel B+E) with indicated AUC, sensitivity and specificity for a random forest classification model (internal cross validation with 1,000 iterations) including all metabolites for cirrhosis (n=149) vs. all HCC (n= 226) (A+B), and cirrhosis (n=149) vs. early-stage HCC (BCLC 0/A, n=181) (D+E) with top 10 candidates based on mean gini ranking (C+F), respectively.

Suppl. Figure 9

A.

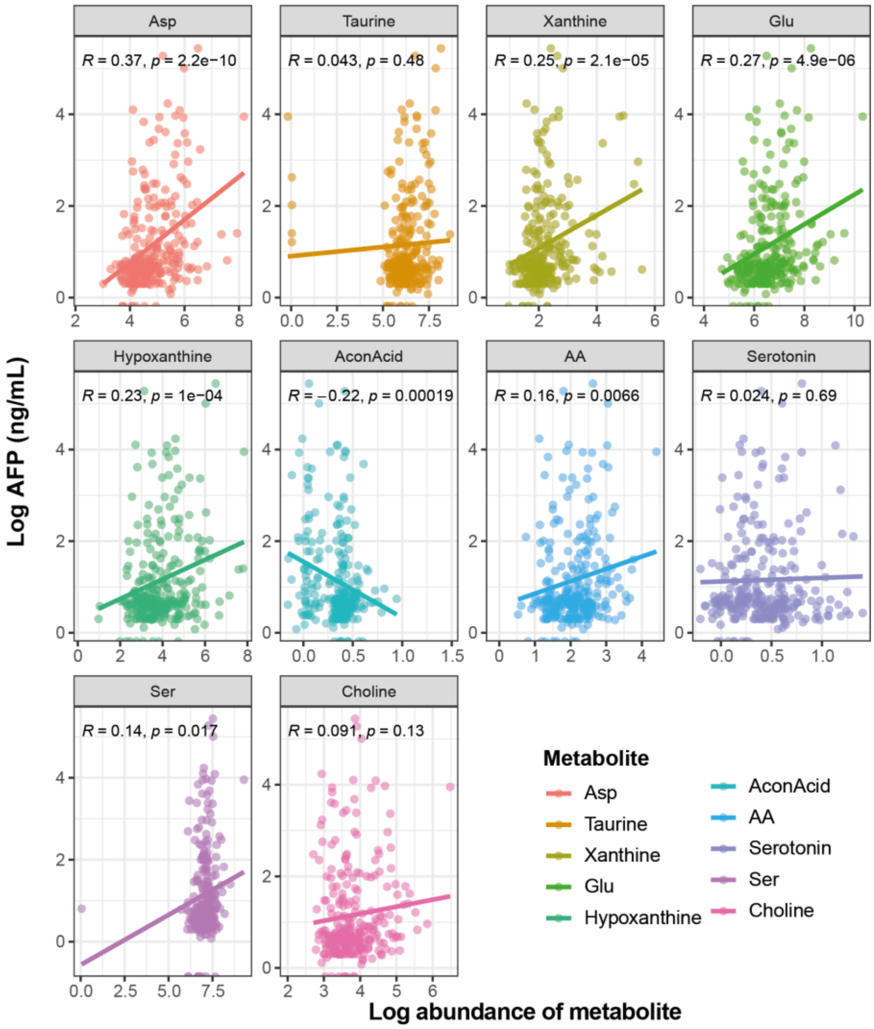

Suppl. Figure 9. (A) correlation of log10 serum AFP levels and log abundance for each metabolite of the 10-metabolite early HCC detection signature.

Suppl. Fig. 10

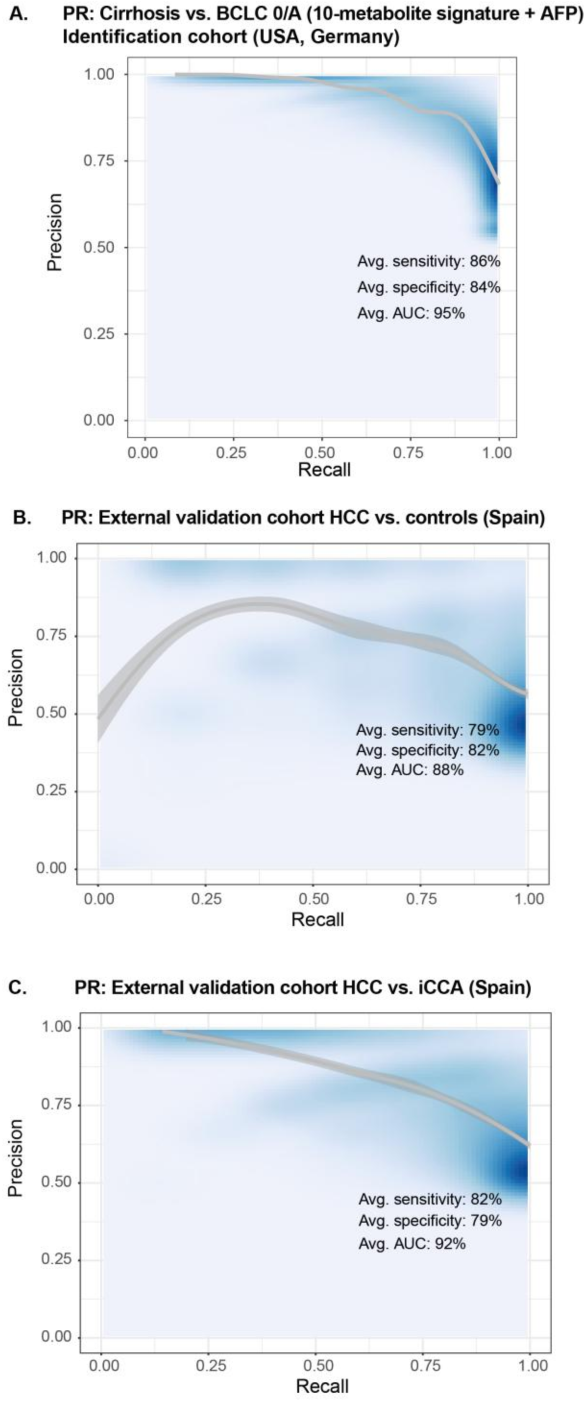

Suppl. Figure 10. Average precision recall curve (PR) with indicated AUC, sensitivity and specificity for a random forest classification model (internal cross validation with 1,000 iterations) including top 10 metabolites plus AFP. (A) “serum metabolome identification cohort”: cirrhosis (n=149) vs. early-stage HCC (BCLC 0/A, n=181). (B) Spanish external validation cohort: controls (n=35) vs. HCC (n=32). (C) Spanish external validation cohort: HCC (n=32) vs. iCCA (n=35). (B+C: signature limited to 5 out of 10 available metabolites in Spanish dataset plus AFP).

Suppl. Figure 11

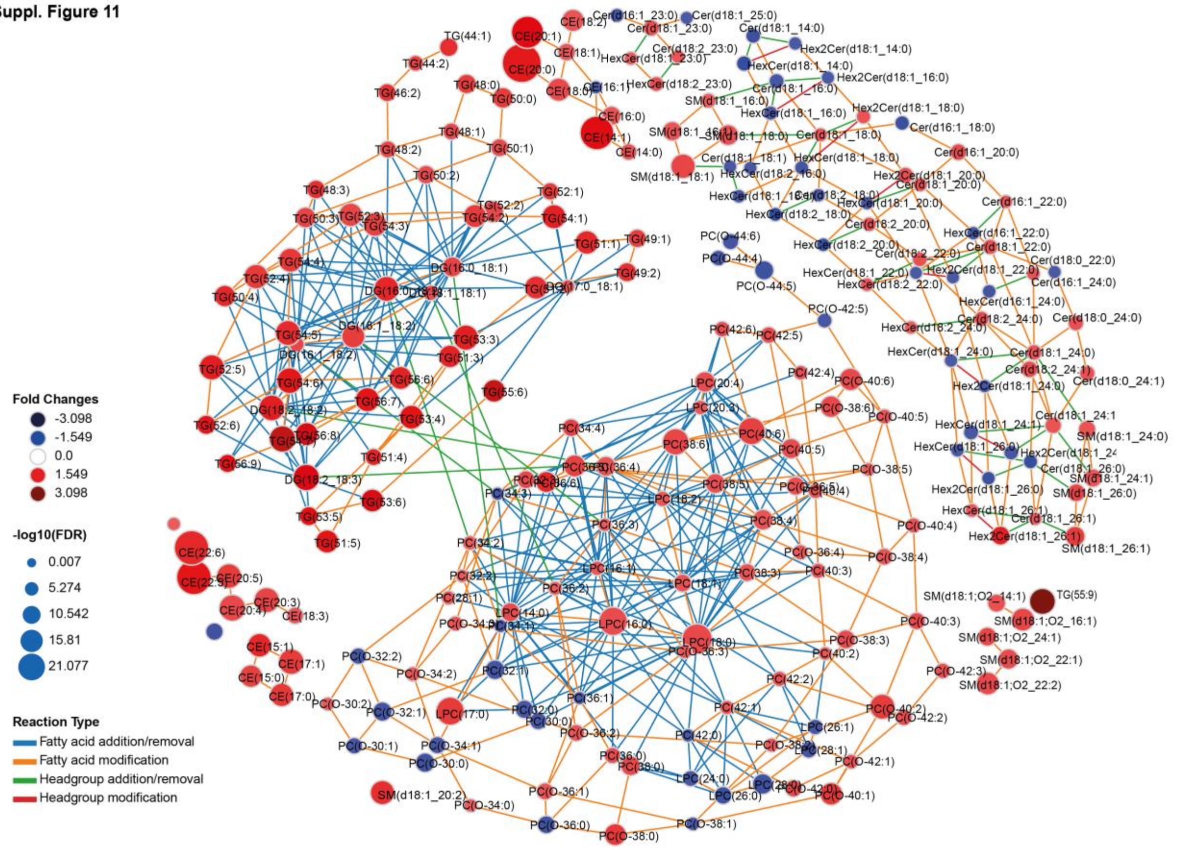

Suppl. Figure 11. Lipid network generated by the LINEX[2] method [18] showing inferred metabolomic reactions between measured serum lipids across patients with HCC (n=226) and cirrhosis without HCC (n=149). Lipids are colored by log2 fold change between HCC and cirrhosis without HCC. Red indicates higher abundance in HCC patients, blue indicates higher abundance in cirrhosis patients. Node size represents the -log10(FDR) value of t-tests between the conditions. Color of lines indicates reaction type.

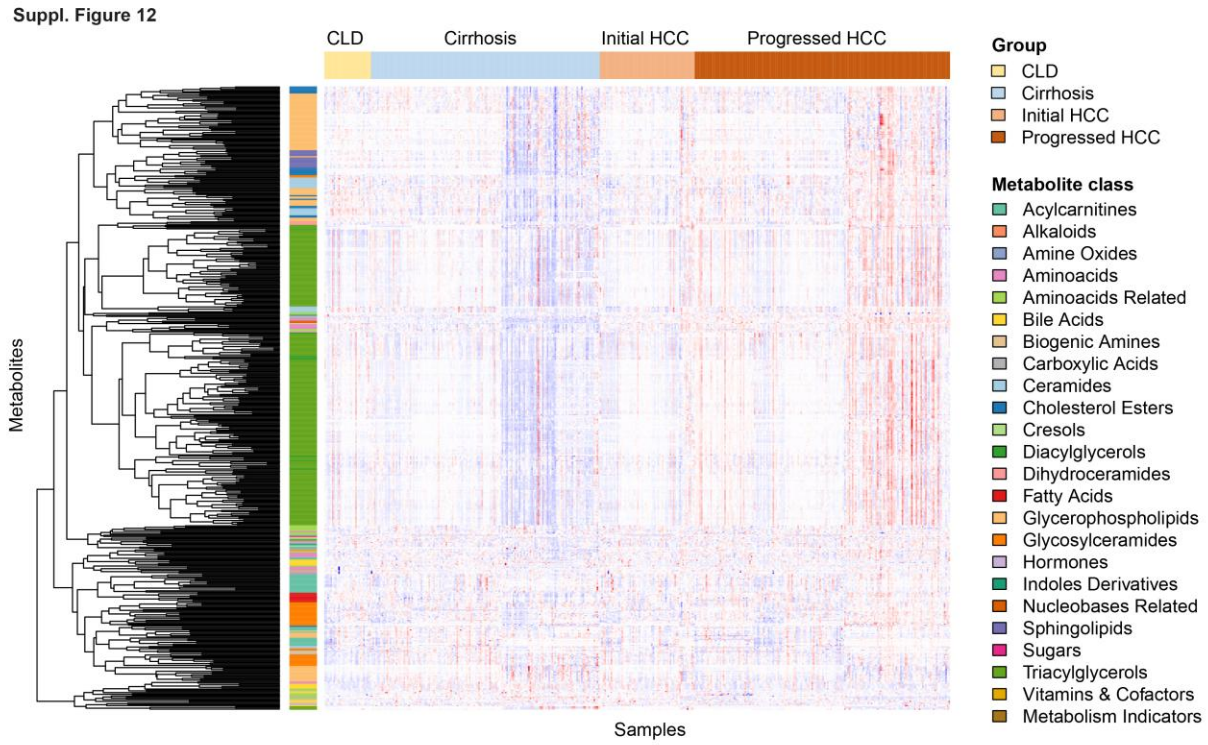

Suppl. Figure 12. Heatmap for all serum metabolites across chronic liver disease (CLD), cirrhosis, initial HCC, and progressed HCC. Metabolites are color-labeled by metabolite class.

Suppl. Figure 13

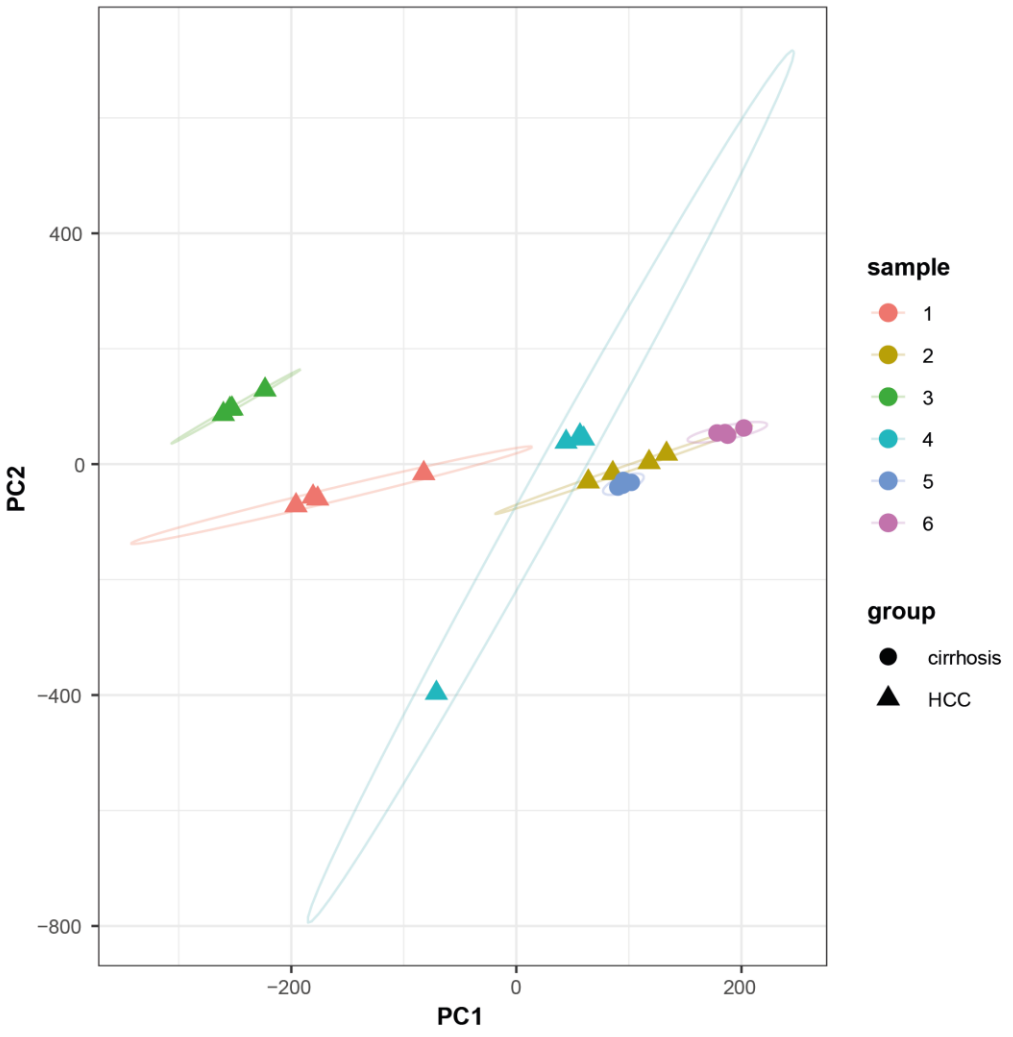

Suppl. Figure 13. Principal component analysis for biological replicates of six patients across four different metabolomic profiling experiments.

Suppl. Figure 14

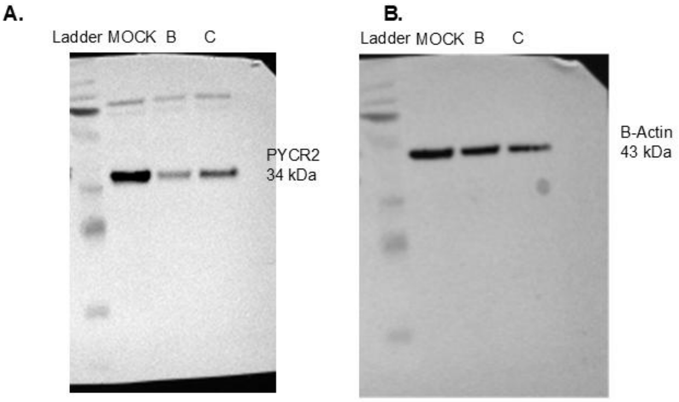

Suppl. Figure 14. Uncropped Western Blot images from Fig 7B. Displayed are representative images, quantification in Fig. 7B is done for 3 experiments.
