# Supplementary material for: Metabolomic liquid biopsy dynamics predict early-stage HCC and actionable candidates of human hepatocarcinogenesis
Source: JHEP Rep. 2025 Jan 30;7(5):101340. doi: 10.1016/j.jhepr.2025.101340 (PMC12023797; doi:10.1016/j.jhepr.2025.101340)
Supplement: Multimedia component 2 [file mmc2.docx]

**JHEP Reports**

**CTAT methods**

Tables for a “Complete, Transparent, Accurate and Timely account” (CTAT) are now mandatory for all revised submissions. The aim is to enhance the reproducibility of methods.

- Only include the parts relevant to your study
- Refer to the CTAT in the main text as ‘Supplementary CTAT Table’
- Do not add subheadings
- Add as many rows as needed to include all information
- Only include one item per row

**If the CTAT form is not relevant to your study, please outline the reasons why:**

|  |
| --- |

- 1. **Antibodies**

| **Name** | **Citation** | **Supplier** | **Cat no.** | **Clone no.** |
| --- | --- | --- | --- | --- |
| rabbit polyclonal anti‑PYCR2 |  | Proteintech, Inc | 17146-1-AP |  |
| monoclonal mouse anti-actin |  | Santa Cruz Biotechnology, Inc | sc-69879 |  |
| HRP-conjugated anti-rabbit IgG |  | Cell Signaling Technology. Inc. | #7074 |  |
| HRP-conjugated anti- mouse IgG |  | Cell Signaling Technology. Inc. | #7076 |  |

- 1. **Cell lines**

| **Name** | **Citation** | **Supplier** | **Cat no.** | **Passage no.** | **Authentication test method** |
| --- | --- | --- | --- | --- | --- |
| HuH7 |  | Wege lab |  |  | short tandem repeat DNA fingerprinting |

- 1. **Organisms**

| **Name** | **Citation** | **Supplier** | **Strain** | **Sex** | **Age** | **Overall n number** |
| --- | --- | --- | --- | --- | --- | --- |
|  |  |  |  |  |  |  |

- 1. **Sequence based reagents**

| **Name** | **Sequence** | **Supplier** |
| --- | --- | --- |
| Human PYCR2 Hs01016460_gH |  | Thermo Fisher |
| Human GAPDH Hs99999905_m1 |  | Thermo Fisher |

- 1. **Biological samples**

| **Description** | **Source** | **Identifier** |
| --- | --- | --- |
|  |  |  |

- 1. **Deposited data**

| **Name of repository** | **Identifier** | **Link** |
| --- | --- | --- |
|  |  |  |

- 1. **Software**

| **Software name** | **Manufacturer** | **Version** |
| --- | --- | --- |
| R studio | R | 4.2 |

- 1. **Other (*e.g*. drugs, proteins, vectors etc.)**

| PYCR2-specific shRNA lentiviral particles packaged from pGFP-C-shLenti vector, clone A | Origene | Catalog No. TL310026VA |
| --- | --- | --- |
| PYCR2-specific shRNA lentiviral particles packaged from pGFP-C-shLenti vector, clone B | Origene | Catalog No. TL310026VB |
| PYCR2-specific shRNA lentiviral particles packaged from pGFP-C-shLenti vector, clone C | Origene | Catalog No. TL310026VC |
| PYCR2-specific shRNA lentiviral particles packaged from pGFP-C-shLenti vector, clone D | Origene | Catalog No. TL310026VD |
| Lenti shRNA scramble particles | Origene | Catalog No. TR30021V |

- 1. **Please provide the details of the corresponding methods author for the manuscript:**

| Johann von Felden, MD  I. Department of Medicine, University Medical Center Hamburg-Eppendorf  Martinistr. 52, 20246 Hamburg, Germany; [j.von-felden@uke.de](mailto:j.von-felden@uke.de) |
| --- |

**2.0 Please confirm for randomised controlled trials all versions of the clinical protocol are included in the submission. These will be published online as supplementary information.**

| NA |
| --- |
